# The Copper Balance of Cities: Exploratory Insights into a European and an Asian City

**DOI:** 10.1111/jiec.12088

**Published:** 2014-06-12

**Authors:** Ulrich Kral, Chih‐Yi Lin, Katharina Kellner, Hwong‐wen Ma, Paul H. Brunner

**Affiliations:** Vienna University of Technology, Institute for Water Quality, Karlsplatz 13/226, Resource and Waste Management, Vienna A-1040 Austria

**Keywords:** cities, environmental protection, industrial ecology, resource efficiency, substance flow analysis (SFA), urban metabolism

## Abstract

Material management faces a dual challenge: on the one hand satisfying large and increasing demands for goods and on the other hand accommodating wastes and emissions in sinks. Hence, the characterization of material flows and stocks is relevant for both improving resource efficiency and environmental protection. This article focuses on the urban scale, a dimension rarely investigated in past metal flow studies. We compare the copper (Cu) metabolism of two cities in different economic states, namely, Vienna (Europe) and Taipei (Asia). Substance flow analysis is used to calculate urban Cu balances in a comprehensive and transparent form. The main difference between Cu in the two cities appears to be the stock: Vienna seems close to saturation with 180 kilograms per capita (kg/cap) and a growth rate of 2% per year. In contrast, the Taipei stock of 30 kg/cap grows rapidly by 26% per year. Even though most Cu is recycled in both cities, bottom ash from municipal solid waste incineration represents an unused Cu potential accounting for 1% to 5% of annual demand. Nonpoint emissions are predominant; up to 50% of the loadings into the sewer system are from nonpoint sources. The results of this research are instrumental for the design of the Cu metabolism in each city. The outcomes serve as a base for identification and recovery of recyclables as well as for directing nonrecyclables to appropriate sinks, avoiding sensitive environmental pathways. The methodology applied is well suited for city benchmarking if sufficient data are available.

**Supplementary Information:**

The online version of this article (doi:10.1111/jiec.12088) contains supplementary material, which is available to authorized users.

## Introduction

One of the main tasks of managing human settlements during the past 10,000 years of urban history was the sufficient supply and disposal of materials. Driven by technology and socioeconomic factors, the interaction of humans with natural resources and the environment has been in continuous change over the years (Agudelo‐Vera et al. 2011). Key developments of the past were the rise of cities and empires after 3000 B.C. and the start of the industrial revolution in the eighteenth century. Global population has been increasing and resource‐intensive lifestyles became predominant in the modern world. One of the pioneers who recognized the consequences of ongoing urbanization at an early stage was Patrick Geddes (Geddes 2010). At the transition from the nineteenth to the twentieth century, he created awareness for the massive flows of resources in cities. By pointing out material losses in the production chain through input‐output (I/O) balancing of material budgets, he was a forerunner of today’s material flow modeler and accountants.

Since that time, several research frameworks have been developed and put forward. Approaches such as material flow analysis (MFA) (Brunner and Rechberger 2004; Baccini and Bader 1996), physical I/O analysis (e.g., Nakamura and Kondo 2009; Nakamura et al. 2007; Hoekstra and Van den Bergh 2006), and environmental‐extended I/O analysis (e.g., Hertwich and Peters 2010; Leontief and Ford 1972) are utilized to investigate the anthropogenic metabolism (Baccini and Brunner 2012). In particular, substance flow analysis (SFA) has been proven to be a practical tool for analyzing urban metal pathways (e.g., Månsson et al. 2009; Henseler et al. 1992; Lindqvist and von Malmborg 2004). SFA tracks the pathway of selected substances through systems such as households, enterprises, cities, or regions. Concerning metal flows in urban areas, a literature review yields two characteristics: First, urban metal flow studies are rare. Recently, Chen and Graedel (2012) reviewed more than 350 SFA articles in a comprehensive manner. They found five cities with insights into metal metabolism. Stockholm is the only city that takes copper (Cu) into account (Bergbäck et al. 2001; Sörme et al. 2001a, 2001b). Additional cities are unexploited even though Cu is a subject of interest because of its relevance from both a resource and environmental point of view. Second, individual city studies are hardly comparable to each other. We extended the scope of literature research and compiled 15 exemplary studies for Cu on an urban scale (see section 1 of the supporting information available on the Journal’s website). A common feature is that they are selective in their scope and that they vary in methodology, such as in terms of system boundaries, modeling approaches, and data acquisition and allocation.

To fill the gap of rare urban Cu studies on the one hand, and comparative city assessments on the other hand, we give exploratory insights into urban Cu balances of different cities. The aims of this study are to (1) develop a methodology to analyze and evaluate the Cu flows and stocks on an urban scale, (2) present and compare the results of a Cu flow and stock analysis for two cities, (3) discuss the differences between the two cities on the basis of selected indicators, and, finally, (4) test the hypothesis that comparing metabolic differences between cities is instrumental for improving decision making regarding resource management and environmental protection.

To reach these objectives, a case‐study approach is applied. Two cities in Europe and Asia, namely, Vienna and Taipei, are chosen as study objects because of their distinct differences, such as population densities and trends, economic developments, culture and lifestyles, and geographical and environmental settings. Explorative data analysis and MFA are used to summarize the characteristics of urban Cu balances in rigid, transparent, and comprehensible form. The procedure chosen reduces complexity of Cu flows and stocks and facilitates the comparison of different cities.

The work contributes to the field of industrial ecology. It gives a substance‐specific understanding of urban resource flows and stocks for city planners and researchers, pointing out the total flows from import into stocks as well as to export out of a city. The individual process descriptions and indicators might appear insufficient in content and level of detail. But, exploring full‐substance balances facilitates the interpretation of dynamic substance turnover in a comprehensive manner. The results serve well the improvement of resource efficiency and environmental performance from an urban systems point of view.

## Materials and Methods

**Figure 1:**
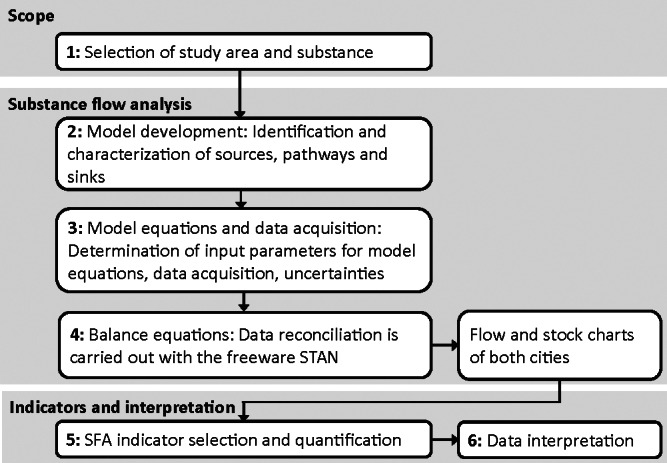
Research framework.

The framework of the study is summarized in figure 1. First, we give reasons for city and substance selection. Second, a generic substance flow model is set up. Third, we use an accounting scheme in order to define model equations on an individual city base. Input parameters are processed with Monte Carlo simulation. Uncertainty ranges of input parameters are considered. Fourth, static modeling is applied for balancing flows and stocks. This yields reconciled data visualized with stock and flow charts. Fifth, substance flow indicators are selected and calculated based on the balanced stock and flow charts. Sixth and last, the differences in the score of the indicators are used as a starting point for interpretation and discussion of distinct urban patterns in each city.

### Scope

#### Choice of Cities and City Characteristics

We have chosen Vienna and Taipei as case‐study regions because of their characteristics in the following areas:

1. Population density in Taipei is more than twice that of Vienna (96 vs. 40 inhabitants per hectare [ha]). Vienna hosts 36% less citizens (1.7 vs. 2.6 million capita) on an area that is 53% larger than in Taipei (41,487 vs. 27,180 ha). The population outlook for the next 50 years expects a 19% decrease for Taipei and a 27% increase for Vienna (Statistik Austria 2010; CEPD 2012).
2. From a cultural and lifestyle point of view, Vienna, as a traditional European city, differs from Taipei, as an Asian city with rather little Western influence. Nevertheless, anthropogenic activities in both cities are service oriented and modern, lacking of heavy industry.
3. The economic power of Vienna and Taipei show similarities such as the magnitude of gross regional product (Vienna, 43,900 Euros per capita; Taipei, 34,800 Euros per capita). This affects the turnover of consumer and investment goods as well as the need for facilities for waste and wastewater treatment and disposal.
4. The geographical and environmental settings of the two cities are different: Vienna is a landlocked city dewatering by the river Danube to the far‐away Black Sea; Taipei is situated on a Pacific island close to the South China Sea. Thus, the so‐called disposal hinterland for the two cities is quite diverse, with an abundant dilution potential for liquid emissions in Taipei and limited capacity for effluents in Vienna.

The two cities are representative of many other cities in the world: If global population is grouped according to the surrounding ecosystems, 65% of people in coastal ecosystems live in urban areas like Taipei. Approximately 45% of people in cultivated ecosystems live in urban areas like Vienna (Marcotullio et al. 2008).

#### Choice of Substance

Resource and environmental aspects are in focus when selecting Cu: This metal is a relevant resource for modern humans. The lifestyle in both cities depends essentially on this technological metal. It is used in many consumer goods such as electric appliances, private and public transport systems, and in infrastructure systems for supply and disposal of water, energy, and information. Because of the high costs of producing Cu from ores, the recovery and recycling of Cu is attractive and is a widely used practice.

Concerning the environment, Cu acts as a tracer for urban emissions. For instance, Cu concentrations in Viennese sewage sludge are significantly larger than in rural areas (Kroiss et al. 2008), and Cu concentrations in Vienna soils are higher than in surrounding rural areas (Pfleiderer 2010). Sörme (2010) describes how modern cities are faced with nonpoint metal emissions. They play an increasing role, compared to emissions from point sources. Because of their high number of sources, they are more difficult to control by regulation than point sources.

### Substance Flow Analysis

We apply a static mass‐balance approach based on materials accounting for two reasons. First, accounting requires reported or measured data sets. In general, quantity and quality of data are better available for past years. So, we selected the years 2008 (Vienna) and 2009 (Taipei) for the ex‐post assessment of material flows. Second, a descriptive framework is most appropriate because of a lack of knowledge regarding deterministic linkages between inflows, stocks, and outflows. From a methodological viewpoint, our approach combines SFA and exploratory data analysis.

#### Model Development

**Figure 2:**
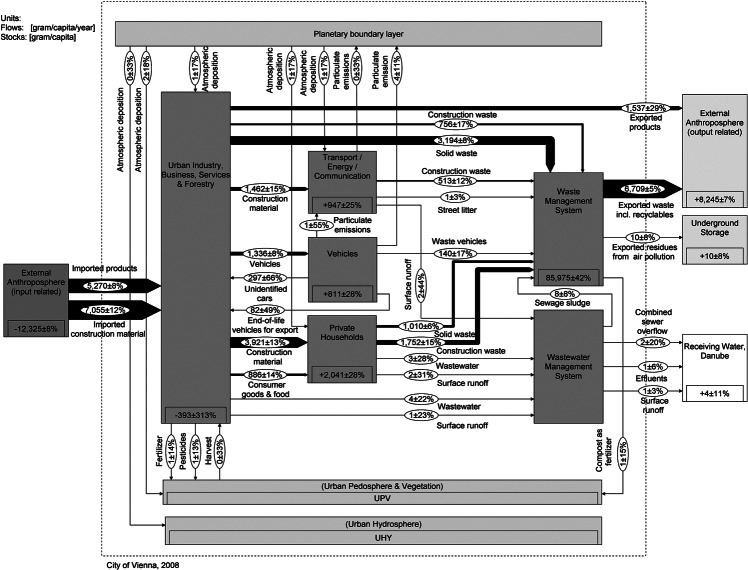
System “copper flows and stocks in Vienna” for the year 2008. Values for flows and changes in stocks are given in grams per capita per year (g/cap/yr) and for stocks in grams per capita (g/cap). The flows are represented as Sankey arrows proportional to the flow rate; figures for stocks are given within the “process” boxes. Numbers have been rounded.

**Figure 3:**
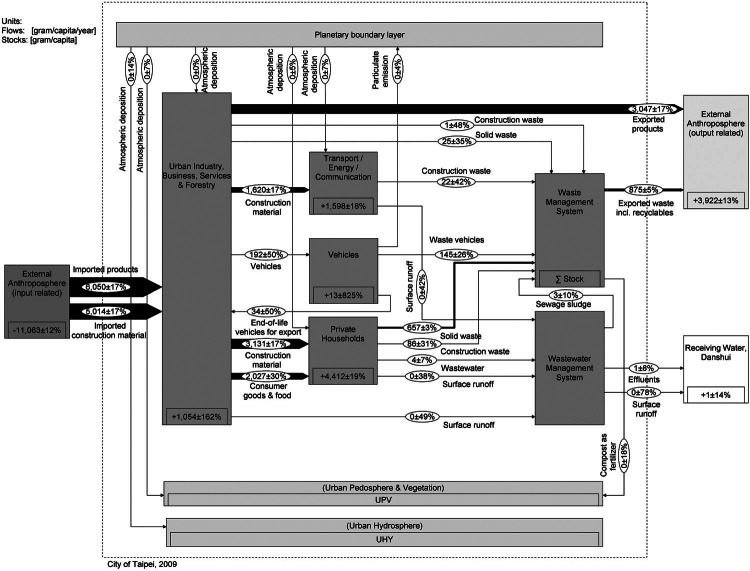
System “copper flows and stocks in Taipei” for the year 2009. Values for flows and changes in stocks are given in grams per capita per year (g/cap/yr) and for stocks in grams per capita (g/cap). The flows are represented as Sankey arrows proportional to the flow rate; figures for stocks are given within the “process” boxes. Numbers have been rounded.

The aim is to present a generic stock and flow chart that allows for comparing the two cities. This requires a common understanding of the key flows and stocks of Cu in a city. Therefore, we set up a model that meets individual urban characteristics of Vienna and Taipei without losing sight of the need to finally compare the data of the two cities. The spatial boundary is set by the administrative city limits, and the data are compiled on an annual basis. The urban systems comprising processes and flows are defined based on previous studies, literature investigations, reports by local municipalities, and expert interviews. All processes and flows are roughly characterized in table 1. The Cu flow models are represented in figures 2 and 3. The

Supporting Information on the Web (see section 2.2.2.1) provides additional, comprehensive descriptions for all flows and stocks.

**Table 1:** Process characterization

*Process name*	*Characterization*
External anthroposphere	Stands for the anthropogenic hinterland of the city; it delivers products and construction material to the city and receives exported products, waste, and recyclables.
Industry, business, services, and forestry	Covers economic activities as well as related buildings; economic activities refer to the trade of goods, material processing, and distribution for final consumption. The buildings are addressed for stock calculation, including construction material and installations.
Transport, energy, and communication infrastructure	Covers immobile infrastructure and corresponding copper stock in transport networks, power grids, and telecommunication networks.
Vehicles	Covers the mobile copper stocks, such as in cars, trucks, bikes, buses, trams, and trains.
Private households	Covers anthropogenic activities of daily life and related buildings; anthropogenic activities refer to residing, nourishing, cleaning, and communication. Related buildings, such as flats and houses, are used for stock calculation of construction material and installations.
Waste management system	Covers the collection, treatment, and disposal of solid waste; the process is disaggregated, which gives further insights into fluxes in view of incineration, composting, and landfilling.
Wastewater management system	Covers the collection and treatment of wastewater; material stocks are not taken into account.
Underground storage	External salt mines out of use act as final storage for hazardous residues from incineration.
Planetary boundary layer	Stands for the lowest part of the atmosphere that is influenced by its contact with the earth’s surface, usually several hundred meters high
Urban pedosphere and vegetation	Consisting of urban soil and vegetation in parks, green areas, and agricultural fields
Urban hydrosphere	Urban water bodies, mainly rivers, groundwater, ponds, and small lakes
Receiving waters	The hydrosphere that takes up both wastewater treatment effluents and combined sewer overflow from the city, such as the river Danube (Vienna) and the Taiwan Straits (Taipei)

#### Model Equations and Data Acquisition

The following model equations are used to calculate each flow and stock:

1 $$ {\dot{m}}_{flow}=f\left({p}_1,{p}_2,{p}_3,\dots, {p}_i\right) $$

2 $$ {m}_{stock}=f\left({q}_1,{q}_2,{q}_3,\dots, {q}_i\right) $$

where ${\dot{m}}_{flow}$ is the Cu flux rate [mass/time], ${m}_{stock}$ is the Cu stock [mass], ${p}_i$ is the input parameter for ${\dot{m}}_{flow}$, and ${q}_i$ is the input parameter for ${m}_{stock}$. The input parameters ${p}_i$ and ${q}_i$ are assumed to be normally distributed with $N\left({m}_{p_i},{s}_{p_i}\right)$ and $N\left({m}_{q_i},{s}_{q_i}\right).$ The mean values *m* and the standard deviation *s* are determined as follows:

- *m* is determined by data mining according to Månsson (2010). The data acquisition procedure prioritizes a bottom‐up approach for both cities. Data availability, quantity, and quality vary between each city. They are even manifold within each city depending on the type of flow and stock. As a common denominator, input parameters representing city characteristics are documented in official statistics. Import data are established through downscaling national import and export statistics. The allocation to city internal processes is based on the global sector share of Cu products and estimations based on local waste statistics. Cu content in products is compiled from literature data, local consumption, and waste statistics. Waste flows are documented in statistics provided by public and private waste companies. Cu fluxes entering and leaving waste management plants, such as incinerators and waste water treatment plants, are documented in scientific reports conducted by the city authority. Emission flows are estimated by the compilation of literature data and inventory databases, such as Ecoinvent. Cu stocks in technical infrastructures are estimated with network lengths and corresponding specific masses. In Vienna, stocks in buildings are based on Swiss per capita data and those in Taipei are based on proxy data from other Taiwanese cities.
- *s* is derived from the uncertainty factor $uf$ according to data vagueness concept from Hedbrant and Sörme (2001): ${s}_p={m_p}^{\ast}\frac{uf-1}{2}\kern0.64em {s}_q={m_q}^{\ast}\frac{uf-1}{2}$ with $uf=1+{0.0036}^{\ast }{e}^{1.105\ast l}$. The uncertainty level *l* ranges from “1” to “5” and depends on the classification of the data source. For example, official statistics on local level are assumed to have low uncertainties ($l=1$). Another example is official statistics on the national level downscaled to the local level with a higher level of uncertainty ($l=3$).

The mean value and standard deviation of each flow (${m}_{{\dot{m}}_{flow}},{s}_{{\dot{m}}_{flow}}\Big)$ and stock (${m}_{m_{stock}},{s}_{m_{stock}}\Big)$ is computed with Monte Carlo simulation by taking into account the model equations and the distribution functions of the input parameters${p}_i$ and ${q}_i$.

The documentation of the SFA model is given in the Supporting Information on the Web. It includes section 2.2.2.1 with a comprehensive description of flows and stocks for both cities. Sections 2.2.2.2 and 2.2.2.3 address the city of Vienna, including two tables: one for the model equations and one for the input parameters. Section 2.2.2.4 provides the background data for the city of Taipei.

#### Balance Equations

We use static model architecture and apply the mass balance principle on each process:

3 $$ 0={\dot{m}}_{input}-{\dot{m}}_{output}+{\dot{m}}_{stock} $$

where ${\dot{m}}_{input}$ is the annual input flow, ${\dot{m}}_{output}$ is the annual output flow, and ${\dot{m}}_{stock}$ is the alteration of stock. Because multiple data sources are used, data quality and quantity are heterogeneous. Consequently, contradictions in fulfilling the mass balance criteria occur. To overcome this gap, we applied the freeware, STAN (Cencic 2010). It uses data reconciliation with an algorithm based on the error propagation law.

#### Stock and Flow Charts of Vienna and Taipei

Figures 2 and 3 show the annual Cu SFA charts. A full list of unbalanced flows and balanced results is provided in the Supporting Information on the Web (see section 2.3).

### Data Analysis and Indicator Selection

Exploratory data analysis stands for analyzing data sets to summarize their main characteristics in an easy‐to‐understand form. We use this tool in combination with indicators for comparative assessment of individual Cu flow data. An indicator is defined to be one or several “observed variables that are used to report a non observable reality” (Loiseau et al. 2012, 214). Our set of indicators represents the interaction of substances within and between the anthroposphere and the environment and form, in part, a base for policy support and decision making for substance management, recycling, and waste management. Table 2 compiles eight indicator groups, including 13 indicators in total. Seven relate to resource efficiency (RE), six relate to environmental protection (EP). The calculation routine is based on the final Cu balances in each city, which comprise 42 flows and four stocks each (figures 2 and 3).

**Table 2:** Indicators and their values for Vienna and Taipei

			*Absolute values*					*Normalized values on a per capita base*				
				*Vienna*		*Taipei*			*Vienna*		*Taipei*	
*No*.	*Indicator*	*Scope*	*Unit*	*Mean*	*Dev (%)*	*Mean*	*Dev (%)*	*Unit*	*Mean*	*Dev*	*Mean*	*Dev*
I	Imports into the cities	RE	t/yr	20,644	8	28,847	12	kg/cap/yr	12.3	0.9	11.1	1.4
II	Stocks and changes in stocks	–	–	–	–	–	–	–	–	–	–	–
	Present urban stock	RE	t	298,000	13	72,051	21	kg/cap	178	24	28	6
	Absolute change in stock	RE	t/yr	5,535	43	18,453	27	kg/cap/yr	3.3	1.4	7.1	1.9
	Relative change in stock	RE	%	2	n.q.	26	n.q.	–	–	–	–	–
III	Wastes and emissions		t/yr	12,370	5	2,450	6	kg/cap/yr	7.4	0.4	0.9	0.1
	Solid waste	RE	t/yr	12,337	5	2,437	6	kg/cap/yr	7.4	0.4	0.9	0.1
	Unintentional emissions	EP	t/yr	28	10	13	6	kg/cap/yr	0.016	2 × 10^−3^	0.005	3 × 10^−4^
	Intentional emissions	EP	t/yr	5	9	0	0	kg/cap/yr	0.003	3 × 10^−4^	0	0
IV	Ratio nonpoint emissions to total emissions	EP	%	51	n.q.	12	n.q.	–	–	–	–	–
V	Flows to sinks		t/yr	14,935	6	10,402	13	kg/cap/yr	8.9	0.5	4.0	0.5
	Anthropogenic	RE	t/yr	14,924	6	10,390	13	kg/cap/yr	8.9	0.5	4.0	0.5
	Environmental	EP	t/yr	12	8	4	13	kg/cap/yr	7.0 × 10^−3^	0.6 × 10^−3^	1 × 10^−3^	0.2 × 10^−3^
VI	Accumulation in urban soil	EP	%	0.07	n.q.	0.03	n.q.	–	–	–	–	–
VII	Removal efficiency by wastewater management	EP	%	65%	n.q.	74%	n.q.	–	–	–	–	–
VIII	Copper content in bottom ash	RE	t/yr	1,097	7	165	34	kg/cap/yr	0.655	0.043	0.063	0.021

Notes: RE = resource efficiency; EP = environmental protection; mean = mean value; dev = standard deviation; n.q. = not quantified; — = not relevant; t = metric tons; t/yr = metric tons per year; % = percent; kg/cap/yr = kilograms per capita per year.

## Results and Discussion

### Overview

Table 2 presents the computed indicator results, including mean value and standard deviations as well as normalized indices on a per capita basis.

### Comparative Assessment and Interpretation of Copper Balances

In the following sections, indicators and their relevance are explained in detail, and results as well as conclusions are presented.

#### Imports into the Cities (I)

Net imports represent the demand and consumption patterns and relate to resource supply as well as to the city’s economic situation. In both consumption‐oriented cities with a comparatively high gross domestic product (GDP), Cu is mainly used in infrastructure and consumer goods. Similar amounts of Cu are imported for Vienna (12 ± 1 kilograms per capita per year [kg/cap/yr]) and Taipei (11 ± 1 kg/cap/yr); both cities rely heavily on Cu import from outside regions. The observed net import rates are six times higher than the average global per capita consumption (Graedel et al. 2004; United Nations 2010).

#### Stocks and Changes in Stocks (II)

Three indicators focus on stocks: (1) the present urban stock and (2) the absolute and (3) relative change in stock.

**Figure 4:**
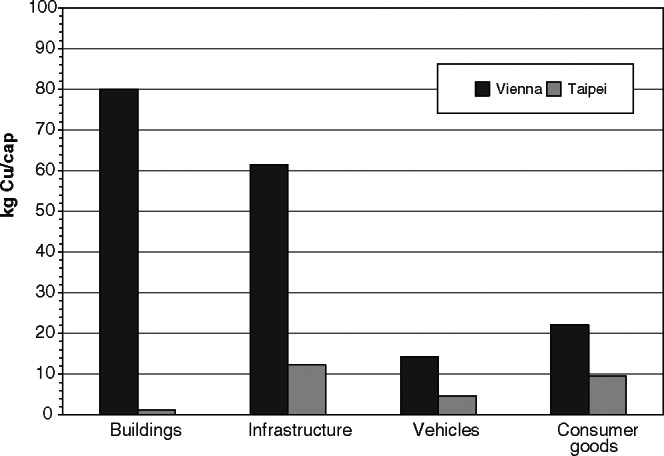
Comparison of copper stocks in Vienna (total stock: 178 kilograms per capita [kg/cap]) and Taipei (total stock: 28 kg/cap). Whereas the residence time of copper in buildings and infrastructure is long (10 to 100 years), it is shorter in vehicles and consumer goods (less than 10 years).

A large part of Cu imports turns into stocks. There are Cu stocks of 178 ± 23 kg/cap in Vienna and of 28 ± 6 kg/cap in Taipei. Similar stocks have been found for other cities in Europe (Sörme et al. 2001a) and in Asia (Zhang et al. 2012). The two cities show different shares of individual Cu stocks (figure 4). Vienna hosts 80% of Cu in long‐term assets. Heating systems as well as networks for electricity and telecommunication are the main Cu carriers. In contrast, Taipei (1) has a much shorter history in urban development and (2) buildings lack heating systems. Forty‐eight percent of the Cu is stored in long‐term goods, such as infrastructures and buildings, and 52% are related to consumer goods, such as air conditioners, cars, and scooters. As a summary, the two cities show marked differences in quantity and relative share in individual sector: Vienna is rich in Cu, and urban mining hotspots are identified in long‐term assets, such as infrastructure and building components. In Taipei, consumer goods have more importance for recovering secondary Cu.

Stock changes denote the annual accumulation or depletion of Cu in various stocks, such as buildings, infrastructure, vehicles, and consumer goods. The indicator represents the economic and technological pattern of a city: As long as a city grows, the input will always be bigger than the output, resulting in a stock increase. An exception is given for a material that is substituted by another, or that is phased out, such as cadmium. For such substances, the input can be smaller than the output, resulting in stock depletion.

The total annual growth rate of the Cu stock is 7 ± 2 kg/cap in Taipei and 3 ± 1 kg/cap in Vienna. In both cities, consumer stocks increase. In Taipei, more Cu is accumulated in private households than infrastructure and vehicles, with sales in the household electronic sector and in electronic appliances in vehicles as the main drivers.

The relative change in stock puts the absolute change in stock (in kg/cap/yr) in relation to present stock (in kg/cap). The relative accumulation of Cu in the stock of Taipei (26%) is more than 10 times higher than in Vienna (2%). In other words, the Cu stock in Vienna is already on a high level and grows only moderately. In Taipei, there is a backlog of demand in the city, resulting in a much faster stock increase, which is mainly a result of private households. For Taipei, the Cu consumption in private households is five times higher than in Vienna, where Cu turnover is determined by maintaining relatively large stocks.

#### Wastes and Emissions (III)

This category comprises three indicators: (1) the amount of Cu in solid wastes, including scrap for recycling, and (2) intentional and (3) unintentional emissions. “Intentional emissions” denominate Cu flows resulting from applications that transport, on purpose, Cu into the environment, such as Cu use in agriculture as a fungicide. “Unintentional emissions” are by‐products resulting from other processes, such as wastewater from households and industry, the release of Cu from catenary wires, or brake pads during the operation of vehicles. These emissions pose a potential threat to the environment.

In Vienna, the total flow of Cu resulting from wastes and emissions is eight times higher than in Taipei (7.4 ± 0.4 vs. 0.9 ± 0.1 kg/cap/yr). In both cities, solid‐waste–borne Cu dominates emissions by more than 99%. Unintentional emissions follow next with less than 1%. These findings are in line with results from a study on dissipative emissions in the United States (Lifset et al. 2012). Lifset and colleagues point out that the recycling rate would increase only by 0.5% if dissipative losses would be recovered and included in recycling, too.

The household waste fractions of Vienna and Taipei are similar (1.0 ± 0.06 vs. 0.7 ± 0.02 kg/cap/yr). In contrast, the fractions from the industrial and construction sector and waste vehicles differ significantly between Vienna (6.4 ± 0.4 kg/cap/yr) and Taipei (0.2 ± 0.1 kg/cap/yr). These findings may be the result of the following three reasons. First, the large and comparatively old stock of Cu in infrastructure and buildings of Vienna must be continuously replaced and acts a source of Cu waste. Second, Taipei has installed a zero waste policy attempting to reduce Cu waste from production. As a third source of uncertainty, statistical data from the two cities about waste flows from the industrial sector have been collected by differing methodologies.

Unintentional Cu emissions in Vienna are three times larger than in Taipei (16 ± 2 grams per capita per year [g/cap/yr] vs. 5 ± 0.3 g/cap/yr). Both airborne emissions and surface runoff in Vienna are approximately 12 times higher than in Taipei. This is because of a higher amount of car mileage and corresponding break wear, catenary wear which is inexistent in Taipei, and the utilization of Cu as a roof and gutter material in Vienna.

#### Ratio of Nonpoint Emissions to Total Emissions (IV)

**Figure 5:**
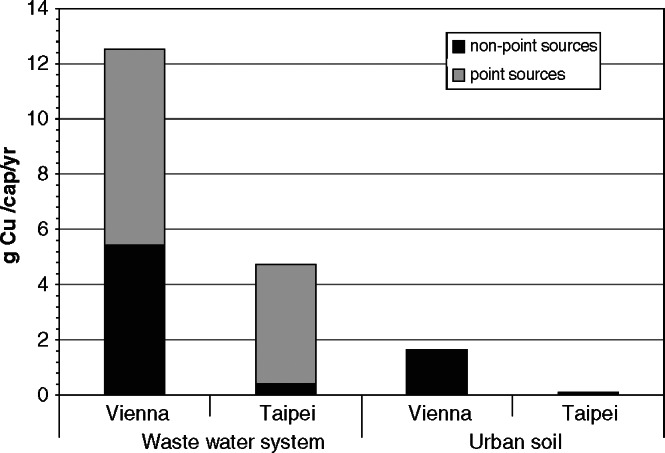
Comparison of copper flows into sinks in Vienna (14 grams per capita per year [g/cap/yr]) and Taipei (5 g/cap/yr) divided into flows entering the wastewater system and soil. Nonpoint sources address waterborne copper in surface runoff and include roof runoff, brake and tire wear from cars, catenary wear, and atmospheric deposition. Point sources relate to waterborne emissions from industry and household, such as urine, feces, consumer products, and pipe corrosion.

Bergbäck (2010) and Sörme and colleagues (2001b) refer to the increasing relevance of nonpoint emissions from the use phase of goods when compared to industrial point source emissions. They report a significant amount of nonpoint emissions for Stockholm and Sweden. The investigators state the difficulties when attempting to regulate nonpoint emissions, such as abrasion from brake pads or corrosion from roofs and gutters. Therefore, we choose as an indicator the proportion of nonpoint emissions to total emissions. Results show that the ratio of nonpoint emissions to total emissions differs between 51% in Vienna and 12% in Taipei (figure 5).

In both cities, the wastewater system receives (1) most of the Cu emissions in town (Vienna, 87%; Taipei, 97%) and (2) more Cu from point sources than from nonpoint sources (Vienna, 57%; Taipei, 91%). Point sources include Cu inputs into the wastewater from private and commercial facilities. In Vienna, approximately 20% of point emissions originate from feces, urine, consumer products, and kitchen waste, 20% from corroding water pipes, and the remaining 60% are related to industrial activities. In Taipei, the relevance of individual point sources is not fully determined because of a lack of local information.

Nonpoint sources cover brake and tire wear, catenary wear, and roof runoff. In Vienna, the annual Cu flow from nonpoint sources is 13 times larger than in Taipei (7.3 vs. 0.6 g/cap/yr). The higher rate is the result of the presence of an extensive tram network, the popularity of Cu roofs and gutters, and higher emissions from low‐duty vehicles. On a per capita basis, the Viennese Cu flow into soil is approximately 15 larger than that in Taipei (1.65 vs. 0.11 g/cap/yr).

Two conclusions can be drawn: First, nonpoint emission patterns play an important role in the case of Vienna, confirming the result of the Swedish studies (Sörme et al. 2001b; Hjortenkrans et al. 2007). This requires that city authorities develop specific long‐term strategies for protecting the environment from nonpoint emissions. Second, because of high ratios of sealed and drained area, the majority of Cu flows is waterborne and collected by the sewer system. Effective end‐of‐pipe technologies, such as appropriate sewage treatment plants, are needed for separating heavy metals in order to control the impact on receiving waters.

#### Flows to Sinks (V)

**Figure 6:**
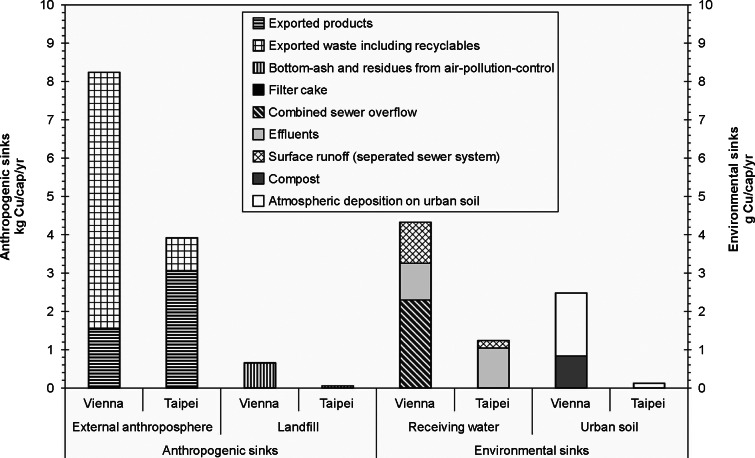
Comparison of copper flows to anthropogenic and environmental sinks in Vienna. The bars for the “anthropogenic sinks” are given in kilograms per capita per year (kg/cap/yr) and those for the “environmental sinks” in grams per capita per year (g/cap/yr). The “External anthroposphere” consists of the hinterland and includes exterior consumption, recycling processes, and underground storage sites outside of Vienna and Taipei. “Receiving water” stands for the river Danube and the Danshui River, respectively.

For this study, anthropogenic sinks and environmental sinks have been taken into account as indicators. In both cities, the main Cu flows to sinks stay within the anthroposphere (more than 99.9%), and less than 0.1% of the total processed output accumulates in the environment (Vienna, 0.08%; Taipei, 0.03%). Figure 6 summarizes the flows to the main sinks.

From the city point of view, anthropogenic sinks consist of (1) landfills within the city limits and of (2) goods, wastes, and recyclables that are processed outside of Vienna and Taipei, respectively. Landfills containing Cu in municipal solid waste (MSW) and bottom ash from MSW incineration (see discussion below on “*Copper content in bottom ash*”) belong to the internal anthroposphere. Cu from recyclables is recovered in the external anthroposphere because there are no Cu recycling facilities within the two investigated cities. The largest amount of Cu leaves the city as a product or solid waste. Taipei exports 78% of Cu in a product form and Vienna 81% of Cu as waste, including recyclables.

Environmental sinks consist of receiving waters and the urban soil within the city limits. The efficiency of the total wastewater system, in terms of separating and directing Cu to appropriate sinks and its relevance for city planning, is further examined when the indicator “*Technical efficiency of the wastewater system*” is discussed below. The soil accumulating Cu by deliberate, as well as unintentional, emissions is included in the following discussion of the indicator “*Accumulation in urban soil*.” The atmosphere as a sink has been neglected as a result of the short residence time of Cu in the air.

#### Accumulation in Urban Soil (VI)

The disparity between the levels of Cu in urban and rural areas has been known for many decades. See, for instance Purves (1966, p. 1077) who is referring to “evidence of slow poisoning of the soil environment in built‐up areas.” The need for observing urban soil conditions for sustainable, long‐term land management is well established (e.g., Wong et al. 2006; Johnson et al. 2011), mostly because analysis of soil samples occasionally showed elevated concentrations compared to rural areas (Pfleiderer 2010; Jien et al. 2011). Because of the lack of such systematic monitoring in Vienna and Taipei, we used a simplified indicator by relating the annual Cu load to the existing Cu stock in the top 30 centimeters of soil of greenspace areas within the city boundaries. Results indicate slow Cu accumulation for the two city soils. The higher accumulation rate in Vienna (0.07% per year) versus Taipei (0.03% per year) may well resemble the bigger emission rate into the air by the Austrian city. Both countries and cities fall short of legal threshold values regarding substance concentrations in urban soil.

Based on the SFA data and the fact that both cities are situated in service‐based and not industrial regions, it can be concluded that the accumulation of Cu in the soil of the two cities is small. Little effects on the pedosphere are expected in the next few centuries. Nevertheless, because there are no appropriate surveying programs in place, it may be that hotspots in the soil exist either by local emissions, geological anomalies, or former anthropogenic inputs. Because the Cu metabolism of Vienna is distinctly different, with more Cu on the surface of buildings and more incorporated in traffic systems, an effective monitoring program should be established first in Vienna in order to ensure that reference values are not surpassed. Site‐specific reference values, based on geogenic background concentrations, are actually being developed in a project called “urban geochemistry of Vienna” (Pfleiderer 2010).

#### Removal Efficiency by Wastewater Management (VII)

In Germany, approximately 35% of Cu input into rivers originates from urban areas (Blondzik et al. 2004). The type of sewer system, its leakage rates, and the treatment technology determine the heavy metal discharge into the hydrosphere. To estimate the efficiency of the wastewater system regarding Cu removal, we relate Cu removed by the wastewater treatment (wwt) system and contained in sewage sludge to the total amount of Cu that has been introduced into the sewer system. SFA calculations yield Cu removal efficiencies of 74% for Taipei and 65% for Vienna. The remaining 26% and, respectively, 35% enter receiving waters by combined sewer overflow, wwt effluent, and surface runoff. Both surface runoff collected by separate sewer systems and combined sewer overflow reach receiving waters directly without treatment. The lower removal efficiency in Vienna is likely the result of larger nonpoint emissions and additional Cu loads from the combined sewer overflow. Based on these results, the following conclusions can be drawn:

- *Wwt systems design*: In order to decrease Cu loadings into the hydrosphere, reduction as well as collection of nonpoint Cu emissions and high removal rates by wwt is important. The comprehensive design of the entire wastewater collection and treatment system becomes crucial for service‐oriented cities where nonpoint sources are the dominant cause of emissions.
- *Monitoring*: There is a lack of accurate data with known uncertainties regarding surface runoff, combined sewer overflow, and stormwater overflow. Few cities establish complete water balances, including collected and uncollected as well as treated and untreated waters and wastewaters. We recommend establishing water balances for cities and using them as a base for decisions regarding environmental management. The city of Berlin serves as an example for a sophisticated precipitation runoff model based on land use and resulting in estimations of location‐based surface runoff (City of Berlin 2010, 2010).
- *Environmental risk assessment*: Cities in general depend on dilution potentials in their hinterland. Vienna uses the river Danube and Taipei the Danshui River for dissipation of Cu contained in purified sewage. Despite that one third to one fourth of sewage‐borne Cu is released to the receiving waters, existing environmental quality standards are observed in both cases. For sediments of the river and sea, there is a lack of both legal standards as well as consistent monitoring data about Cu concentrations (e.g., for Vienna, Kavka et al. 2000). In order to prevent future overloading, environmental fate and impact models could be used for predicting the evolution of heavy metal concentrations over time.

#### Copper Content in Bottom Ash (VIII)

In both cities, municipal solid waste is incinerated in waste to energy plants. During incineration, most of the Cu contained in MSW is transferred to bottom ash. To recover Cu from bottom ash offers two advantages: First, it contributes to resource conservation. Second, it is instrumental for minimizing Cu flows to the environment in case bottom ash is landfilled or reused as a construction material.

In Vienna, bottom ash is stabilized with cement and disposed of in the municipal landfill. In Taipei, bottom ash is used as a base material for road construction as well as a fine aggregate in asphalt (Taipei County Government 2010). The per capita Cu flux in Vienna is approximately 10 times larger than in Taipei (655 ± 43 vs. 63 ± 21 g/cap/yr), corresponding to approximately 5% (Vienna) and 1% (Taipei) of annual Cu consumption. The market value of Cu contained in bottom ash equals roughly US$8.8 million per year in Vienna and US$1.4 million per year in Taipei.

As a result of the value of Cu and other metals, economic incentives exist to recover valuable elements, such as Cu, aluminum, gold, and silver, from bottom ash. Substance concentrations are several times higher than in natural ores (Simon 2010; Jordi 2004) and can be considerably increased by bottom ash treatment (e.g., Muchova et al. 2009; Morf et al. 2013; Shen and Forssberg 2003). Recent experiences favor dry discharge of bottom ash for efficient recovery of nonferrous metals (Morf et al. 2013). This technology is not yet implemented in Vienna and Taipei.

Another means to recover valuable metals from MSW is landfill mining, that is, the excavation of materials from old MSW or bottom ash landfills. From an economic point of view, landfill mining appears to be attractive only if additional values are created. This could be driven by gaining new land for building sites or reduced costs for long‐term landfill after care. Thus, recovery projects that aim exclusively at recovering resources are rare (Hölzle 2010). Assuming a period of 20 years and a waste generation rate of one kilogram of MSW per person and day, 24,000 tonnes^1^ of Cu with an economic value of US$200 million have been accumulated in Viennese landfills.

In addition to recovery of Cu from bottom ash and landfills, Cu can also be recovered by separate collection of Cu containing waste fractions, such as waste electrical and electronic equipment. In fact, this practice is widely applied and is the most favored by European waste policy. For best effectiveness of a comprehensive Cu recovery system, the main carriers of Cu (Morf and Taverna 2006), as well as the efficiencies and costs of recovery technologies, must be known. It remains to be determined which of the recovery pathways reaches the goals of waste management “resource recovery” and “environmental protection” at the least costs.

## Conclusions

This article presents the results of investigations into urban Cu flows and stocks in two different cities. It provides a starting point for comparing additional cities and metals. The mass balance approach, focusing on the main processes, Cu flows, and stocks, goes beyond the city as a black‐box model. To our knowledge, this study is the most comprehensive Cu balance on the urban level, encompassing Cu flows from imports to stocks in use and exports. Also, it is the first comparison of the Cu metabolism of two cities. Based on the results, we discuss reasons for differences in the Cu balances of Vienna and Taipei and give recommendations for the management of Cu as a resource and potential environmental pollutant.

First, we find typical characteristics in the dynamics of Cu stock changes. Rapid growth in a young city such as Taipei is characterized by low amounts of Cu stocks and relatively high annual stock increases. In contrast, Cu stocks in older Vienna are relatively high, and thus the relative stock change is smaller than in Taipei. Cu demand and disposal in Vienna is mainly the result of maintenance and replacement. The relative importance of Cu in consumer goods decreases.

Second, much Cu is recycled, but there are still recovery potentials available. Bottom ash from waste incineration is an example for both cities. In order to reach sustainability goals such as resource conservation and long‐term environmental protection, recovering more Cu from MSW and other wastes is mandatory. On the one hand, especially if combined with the recovery of additional metals such as aluminum, gold, and silver, this may result in economic benefits. On the other hand, separation of Cu together with other metals, such as chromium, lead, and cadmium reduces the concentrations of heavy metals in bottom ash, making this material more suitable for utilization as a construction material.

Third, some Cu is emitted diffusively by wear, corrosion, and weathering of Cu built into infrastructure, transport systems, and others. These emissions partly enter the wastewater system and partly accumulate in the urban soil. Nonpoint emissions are not yet in the focus of urban governance, even though, for example, the European Union urges member states to take nonpoint emission into account. Based on the experiences of the studies in Stockholm, Vienna, and Taipei, it is recommended to monitor the concentrations of Cu and other heavy metals in urban soils and sediments by a combination of SFA and direct measurements.

Fourth, designing urban wastewater systems in a comprehensive way is crucial for minimizing the loading of receiving waters with Cu and other heavy metals. The combination of wastewater collection systems and treatment technologies determine the total efficiency in terms of removing pollutants. For effective control of heavy metal flows to receiving waters, the entire systems performance is more important than the removal efficiency of wastewater treatment alone.

Fifth, this study has shown that transnational collaboration yields new insights into the substance balances of cities. Authorities and researchers profit from each other through a common research framework for comparing urban metabolism data. We recommend sharing experiences and discussing methodology for urban metabolism studies and developing common generic models as well as data acquisition procedures.

## Supplementary Information

**Supporting Information S1:** This supporting information provides a large quantity of background material that complements the methods and findings included in the main research article, including a compilation of 15 exemplary studies for copper (Cu) on an urban scale, comprehensive descriptions of all flows and stocks for the study, documentation on the substance flow analysis (SFA) model used, and a full list of unbalanced flows and balanced results for this study.

## Footnotes

1 The term tonne refers to metric ton. One tonne (t) = 10^3^ kilograms (kg, SI) ≈ 1.1 short tons.

## References

[CR1] Agudelo‐Vera, C. M., A. R. Mels, K. J. Keesman, and H. H. M. Rijnaarts. 2011. Resource management as a key factor for sustainable urban planning. *Journal of Environmental Management* 92(10): 2295–2303. 21641714 10.1016/j.jenvman.2011.05.016

[CR2] Baccini, P. and H. P. Bader. 1996. *Regionaler Stoffhaushalt*. [Regional metabolism.] Heidelberg, Berlin, Oxford: Spektrum, Akad, Verlag.

[CR3] Baccini, P. and P. H. Brunner. 2012. *Metabolism of the anthroposphere: Analysis, evaluation, design*, 2nd ed. Cambridge, MA, USA: MIT Press.

[CR4] Bergbäck, B. 1992. Industrial metabolism: The emerging landscape of heavy metal emissions in Sweden. Ph.D. thesis, Linköping University, Linköping, Sweden.

[CR5] Bergbäck, B., K. Johansson, and U. Mohlander. 2001. Urban metal flows—A case study of Stockholm. Review and conclusions. *Water, Air, and Soil Pollution: Focus* 1(3): 3–24.

[CR6] Blondzik, K., U. Claussen, C. Füll, and J. Heidemeier. 2004. *Umweltpolitik: Die Wasserrahmenrichtlinie—Neues Fundament für den Gewässerschutz in Europa*. [Environmental policy: The water framework directive—A new baseline for water protection in Europe.] Berlin: Bundesministerium für Umwelt, Naturschutz und Reaktorsicherheit.

[CR7] Brunner, P. H. and H. Rechberger. 2004. *Practical handbook of material flow analysis*. Boca Raton, FL, USA: CRC Press.

[CR8] CEPD (Council for Economic Planning and Development). 2012. *Estimation of population in Taiwan from 2012 to 2060 (in Chinese)*. Taipei: CEPD, Council for Economic Planning and Development, Executive Yuan.

[CR9] Cencic, O. 2012. Software platform STAN (short for subSTance flow ANalysis). www.stan2web.net. Accessed 28 September 2012.

[CR10] Chen, W.‐Q. and T. E. Graedel. 2012. Anthropogenic cycles of the elements: A critical review. *Environmental Science & Technology* 46(16): 8574–8586. 22803614 10.1021/es3010333

[CR11] City of Berlin. 2012a. Flow chart of the ABIMO model. www.stadtentwicklung.berlin.de/umwelt/umweltatlas/extra/213eb02.htm. Accessed 11 January 2013.

[CR12] City of Berlin. 2012b. Berlin environmental atlas—Surface runoff, percolation, total runoff and evaporation from precipitation. www.stadtentwicklung.berlin.de/umwelt/umweltatlas/edb213_05.htm. Accessed 11 January 2013.

[CR13] Geddes, P. 1885. An analysis of the principles of economics. *Proceedings of the Royal Society of Edinburgh Vol. XII*, edited by Royal Society of Edinburgh, Edinburgh, UK.

[CR14] Graedel, T., D. Van Beers, M. Bertram, K. Fuse, R. Gordon, A. Gritsinin, A. Kapur, R. Klee, R. Lifset, and L. Memon. 2004. Multilevel cycle of anthropogenic copper. *Environmental Science & Technology* 38(4): 1242–1252. 14998044 10.1021/es030433c

[CR15] Hedbrant, J. and L. Sörme. 2001. Data vagueness and uncertainties in urban heavy‐metal data collection. *Water, Air, & Soil Pollution: Focus* 1(3): 43–53.

[CR16] Henseler, G., R. Scheidegger, and P. H. Brunner. 1992. Die bestimmung von Stoffflüssen im Wasserhaushalt einer Region. [Determination of material flux through the hydrosphere of a region.] *Vom Wasser* 78: 91–116.

[CR17] Hertwich, E. and G. P. Peters. 2010. Multiregional Input‐Output Database (OPEN:EU technical document). www.oneplaneteconomynetwork.org/resources/programme‐documents/WP1_MRIO_Technical_Document.pdf. Accessed 2 December 2012. Trondheim, Norway: One Planet Economy Network.

[CR18] Hjortenkrans, D. S. T., B. G. Bergbäck, and A. V. Häggerud. 2007. Metal emissions from brake linings and tires: Case studies of Stockholm, Sweden 1995/1998 and 2005. *Environmental Science & Technology* 41(15): 5224–5230. 17822083 10.1021/es070198o

[CR19] Hoekstra, R. and J. C. J. M. van den Bergh. 2006. Constructing physical input‐output tables for environmental modeling and accounting: Framework and illustrations. *Ecological Economics* 59(3): 375–393.

[CR20] Hölzle, I. 2010. Vom Deponierückbau bis zum landfill mining—eine Synthese internationaler Untersuchungen. [Landfill dismantling and landfill mining—A synthesis of international investigations.] *Österreichische Wasser‐ und Abfallwirtschaft* 62(7–8): 155–161.

[CR21] Jien, S. H., C. C. Tsai, Z. Y. Hseu, and Z. S. Chen. 2011. Baseline concentrations of toxic elements in metropolitan park soils of Taiwan. *Terrestrial and Aquatic Environmental Toxicology* 5(1): 1–7.

[CR22] Johnson, C. C., A. Demetriades, J. Locutura, and R. T. Ottesen (eds.). 2011. *Mapping the chemical environment of urban areas*. Chichester, UK: John Wiley & Sons, Ltd.

[CR23] Jordi, B. 2004. Recycling—die Rohstoffquelle der Zukunft. [Recycling—a future source of resources.] *Magazin Umwelt 1/2004: Produkte und Konsum*. Bern, Schweiz: Bundesamt für Umwelt (BAFU).

[CR24] Kavka, G., D. Krämer, P. Kreitner, R. Mauthner‐Weber, G. Ofenböeck, and A. Rauchbüchl. 2000. *Wasserbeschaffenheit und Güte der Österreichischen Donau unter besonderer Berücksichtigung der langzeitlichen Entwicklung*. [Water quality of the Danube under long term considerations.] Wien: Bundesamt für Wasserwirtschaft.

[CR25] Kroiss, H., L. S. Morf, C. Lampert, and M. Zessner. 2008. *Optimiertes Stoffflussmonitoring für die Abwasserentsorgung Wiens*. [Optimized substance flow monitoring of the waste water treatment plant in Vienna.] Vienna: Technische Universität Wien.

[CR26] Leontief, W. and D. Ford. 1972. Air pollution and the economic structure: Empirical results of input‐output computations. *Fifth International Conference on Input‐Output Techniques*, January 1971, Geneva. Amsterdam: North Holland Publishing Company.

[CR27] Lifset, R. J., M. J. Eckelman, E. M. Harper, Z. Hausfather, and G. Urbina. 2012. Metal lost and found: Dissipative uses and releases of copper in the United States 1975–2000. *Science of The Total Environment* 417–418: 138–147. 10.1016/j.scitotenv.2011.09.075 22248854

[CR28] Lindqvist, A. and F. von Malmborg. 2004. What can we learn from local substance flow analyses? The review of cadmium flows in Swedish municipalities. *Journal of Cleaner Production* 12(8–10): 909–918.

[CR29] Loiseau, E., G. Junqua, P. Roux, and V. Bellon‐Maurel. 2012. Environmental assessment of a territory: An overview of existing tools and methods. *Journal of Environmental Management* 112: 213–225. 22929644 10.1016/j.jenvman.2012.07.024

[CR30] Månsson, N. 2009. Substance flow analysis of metals and organic compounds in an urban area. Ph.D. thesis, School of Pure and Applied Natural Sciences, University of Kalmar, Kalmar, Sweden.

[CR31] Månsson, N., B. Bergbäck, D. Hjortenkrans, A. Jamtrot, and L. Sörme. 2009. Utility of substance stock and flow studies. *Journal of Industrial Ecology* 13(5): 674–686.

[CR32] Marcotullio, P. J., A. K. Braimoh, and T. Onishi. 2008. The impact of urbanization on soils. In *Land use and soil resources*, edited by A. K. Braimoh and P. L. G. Vlek. Dordrecht, the Netherlands: Springer Science+Business Media B.V.

[CR33] Morf, L. S. and R. Taverna. 2006. *Monitoringkonzept zur Ermittlung von Ursachen für Veränderungen der Schwermetallgehalte im Wiener Restmüll*. [Monitoring concept to investigate the change in heavy metal concentrations in residual waste of Vienna.] Zürich, Schweiz: GEO Partner AG Umweltmanagement.

[CR34] Morf, L. S., R. Gloor, O. Haag, M. Haupt, S. Skutan, F. D. Lorenzo, and D. Böni. 2013. Precious metals and rare earth elements in municipal solid waste—Sources and fate in a Swiss incineration plant. *Waste Management* 33(3): 634–644. 23085306 10.1016/j.wasman.2012.09.010

[CR35] Muchova, L., E. Bakker, and P. Rem. 2009. Precious metals in municipal solid waste incineration bottom ash. *Water, Air, & Soil Pollution: Focus* 9(1–2): 107–116.

[CR36] Nakamura, S. and Y. Kondo. 2009. Waste input‐output analysis: Concepts and application to industrial ecology. In *Eco‐efficiency in industry and science*, Vol. 26, edited by A. Tukker. Dordrecht, the Netherlands: Springer Science+Business Media B.V.

[CR37] Nakamura, S., K. Nakajima, Y. Kondo, and T. Nagasaka. 2007. The waste input‐output approach to materials flow analysis. *Journal of Industrial Ecology* 11(4): 50–63.

[CR38] Pfleiderer, S. 2011. Umweltgeochemie Stadtgebiet Wien. [Environmental geochemistry of the city of Vienna, restricted for public access.] *Seminar Ressourcenmanagement und Abfallwirtschaft* [Seminar Resources and Waste Management], 4 June 2011, Vienna.

[CR39] Purves, D. 1966. Contamination of urban garden soils with copper and boron. *Nature* 210(5040): 1077–1077.5914918

[CR40] Shen, H. and E. Forssberg. 2003. An overview of recovery of metals from slags. *Waste Management* 23(10): 933–949. 14614927 10.1016/S0956-053X(02)00164-2

[CR41] Simon, F. G. 1996. Geld in der Kehrichtschlacke. [Money in bottom‐ash.] *Neue Zürcher Zeitung, Beilage Forschung und Technik*, *134* [Zürich], 6 December 1996.

[CR42] Sörme, L. 2003. Urban heavy metals: Stocks and flows. Ph.D. thesis, The Tema Institute, Department of Water and Environmental Studies, Linköping University, Linköping, Sweden.

[CR43] Sörme, L., B. Bergbäck, and U. Lohm. 2001a. Century perspective of heavy metal use in urban areas. A case study in Stockholm. *Water, Air and Soil Pollution: Focus* 1(3–4): 197–211.

[CR44] Sörme, L., B. Bergbäck, and U. Lohm. 2001b. Goods in the anthroposphere as a metal emission source: A case study of Stockholm, Sweden. *Water, Air, and Soil Pollution: Focus* 1(3): 213–227.

[CR45] Statistik Austria. 2013. Vorausberechnete Bevölkerungsstruktur für Wien 2011–2075 laut Hauptszenario. [Population outlook for Vienna 2011–2075 according to the main scencario.] www.statistik.at/web_de/statistiken/bevoelkerung/demographische_prognosen/bevoelkerungsprognosen/index.html. Accessed 31 January 2013.

[CR46] Taipei County Government 2010. *Decision analysis of bottom‐ash Reuse strategic planning [in Chinese]*. Taipei: Environmental Protection Department of Taipei County Government (New Taipei City Government).

[CR47] United Nations. 2010. World population prospects, the 2010 revision. http://esa.un.org. Accessed 16 January 2013.

[CR48] Wong, C. S. C., X. Li, and I. Thornton. 2006. Urban environmental geochemistry of trace metals. *Environmental Pollution* 142(1): 1–16. 16297517 10.1016/j.envpol.2005.09.004

[CR49] Zhang, L., Z. Yuan, and J. Bi. 2012. Estimation of copper in‐use stocks in Nanjing, China. *Journal of Industrial Ecology* 16(2): 191–202.

