# Supplementary material for: The Copper Balance of Cities: Exploratory Insights into a European and an Asian City
Source: J Ind Ecol. 2014 Jun 12;18(3):432–44. doi: 10.1111/jiec.12088 (PMC4386478; doi:10.1111/jiec.12088)
Supplement: Supplementary file 1 — Supporting Information S1: This supporting information provides a large quantity of background material that complements the methods and findings included in the main research article, including a compilation of 15 exemplary studies for copper (Cu) on an urban scale, comprehensive descriptions of all flows and stocks for the study, documentation on the substance flow analysis (SFA) model used, and a full list of unbalanced flows and balanced results for this study. [file 44498_2014_1803011_MOESM1_ESM.pdf]

SUPPORTING INFORMATION FOR:

Kral, U., C.-Y. Lin, K. Kellner, H.-W. Ma, P.H. Brunner. 2013. The copper balance of cities: Exploratory insights into a European and an Asian city. *Journal of Industrial Ecology.*

Summary

This supporting information provides a large quantity of background material that complements the methods and findings included in the main research article, including: a compilation of 15 exemplary studies for copper (Cu) on an urban scale; comprehensive descriptions of all flows and stocks for the study; documentation on the substance flow analysis (SFA) model used; and a full list of unbalanced flows and balanced results for this study.

Table of Contents

1. Introduction 4
2. Material and methods 5
2.1 City selection and city characteristics 5
2.2 Substance flow modeling 5
2.2.1 Model development 5
2.2.2 Model equations and data acquisition 6
2.2.2.1 Flows & stock description for Vienna and Taipei 6
2.2.2.1.1 Atmospheric Deposition (ADE PBLUHY, ADE PBLUPV, ADE PBLECO, ADE PBLPHH, ADE PBLTEC) 6
2.2.2.1.2 Products (PRO EXAECO, PRO ECOPHH) 6
2.2.2.1.3 Construction material (CON EXAECO, CON ECOTEC, CON ECOPHH, CWA ECOCTS, CWA TECCTS, CWA PHHCTS, CWA CTSLDF) 7
2.2.2.1.4 Vehicles (VEH ECOVEH, WAS VEHCTS, VEH UID, VEH VEHECO) 8
2.2.2.1.5 Particulate emissions (PAE VEHTEC, PAE VEHPBL, PAE TECPBL) 8
2.2.2.1.6 Waste Water flows (SRO ECOCTS, SRO PHHWWS, SRO TECWWS, SRO WMIHYD WWS ECOWWS, WWS PHHWWS, SSL WWSCTS, SPW WWSHYD, EFF WWSHYD) 9
2.2.2.1.7 Solid waste (HHW PHHCTS, WAS ECOCTS) 10
2.2.2.1.8 Exported products (PRO ECOEXA) 11
2.2.2.1.9 Exported waste including recyclables (WAS CTSEXA) 11
2.2.2.1.10 Incineration: Mixed waste, exported residues for underground storage, residues, disposable waste (WAS CTSINC, TTR INCEXA, RES INCLDF) 11
2.2.2.1.11 Composting: Compostable waste, residues, compost as fertilizer (WAS CTSCOM, RES COMCTS, COM COMUPV) 12
2.2.2.1.12 Pesticides (PES ECOUPV) 12
2.2.2.1.13 Fertilizer (FER ECOUPV) 13
2.2.2.1.14 Technical Infrastructure (TECstock) 13
2.2.2.1.15 Economy and private households (ECOstock, PHHstock) 13
2.2.2.1.16 Vehicles (VEHstock) 14
2.2.2.1.17 Urban Soil (UPVstock) 14
2.2.2.2 Vienna – List of mathematical model equations and input parameters 15
2.2.2.3 Vienna - Background data 20
2.2.2.3.1 Products (PRO EXAECO) 20
2.2.2.3.2 Construction material (CON EXAECO, CON ECOPHH, CON ECOTEC) 24
2.2.2.3.3 Construction waste (CWA ECOCTS, CWA PHHCTS, CWA TECCTS) 25
2.2.2.3.4 Vehicles 26
2.2.2.3.5 Particulate Emissions 27
2.2.2.3.6 Overview 27
2.2.2.3.7 Brake wear 28
2.2.2.3.8 Catenary wear 29
2.2.2.3.9 Roof runoff 30
2.2.2.3.10 Waste water flows 31
2.2.2.3.11 Solid Waste 32
2.2.2.3.12 WEEE 34
2.2.2.3.13 Exported products (PRO ANTEXA) 34
2.2.2.3.14 Exported waste including recyclables (REC CTSECO) 34
2.2.2.3.15 Harvest 35
2.2.2.3.16 Pesticides 36
2.2.2.3.17 Fertilizer 36
2.2.2.3.18 Cu stock in technical infrastructure (TECstock) 36
2.2.2.3.19 Cu stock in Vehicles (VEHstock) 37
2.2.2.3.20 Cu stock in Landfills (LDFstock) 37
2.2.2.3.21 Cu stock in urban soil (UPVstock) 39

2.2.2.4 Taipei – Background data 40
2.2.2.4.1 Atmospheric Deposition (ADE PBLUHY, ADE PBLUPV, ADE PBLECO, ADE PBLPHH, ADE PBLTEC) 40
2.2.2.4.2 Products (PRO EXAECO, PRO ECOPHH) 40
2.2.2.4.3 Construction Material (CON EXAECO, CON ECOTEC, CONECOPHH, CWA ECOCTS, CWA TECCTS, CWA PHHCTS) 42
2.2.2.4.4 Vehicles (VEH ECOVEH, WAS VEHCTS, VEH UID, VEH VEHECO) 42
2.2.2.4.5 Solid waste (HWW PHHCTS, WAS ECOCTS) 43
2.2.2.4.6 Incineration: Mixed waste, exported residues for underground storage, residues Disposable waste (WAS CTSINC, TTR INCEXA, RES INCLDF) 43
2.2.2.4.7 Composting: Compostable waste, residues, compost as fertilizer (WAS CTSCOM, RES COMCTS, COM COMUPV) 44
2.2.2.4.8 Stocks in ECO, PHH 44
2.2.2.4.9 Stocks in VEH 44
2.3 Stock and flow results 46
3. References 50

1. INTRODUCTION

Table S1: Examples of Cu flow studies on urban scale. They vary in terms of scope, modeling and data acquisition framework.

Analytical entities	City	Focus	Reference
stocks	Nanjing (CN)	Supply-restrictions of Cu in China lead to the identification of potential reservoirs of secondary Cu resources in urban infrastructure.	(Zhang et al. 2012)
	Stockholm (SE)	Pathways and stock are investigated comprehensively.	(Sörme et al. 2001a; Bergbäck et al. 2001; Sörme et al. 2001b)
	Linkoping (SE)	Stocks-in-use and hibernating stocks in the telecommunication network are estimated in view of the recovering potential.	(Krook et al. 2011)
	Cape Town (ZA)	Stocks-in-use of major appliances are linked with product lifetimes in order to predict future waste flows and recovering potentials.	(van Beers and Graedel 2003)
emissions and/or waste flows	Nanjing (CN)	Cu impacts on road sediments are analyzed.	(Zuo et al. 2012)
	Stockholm (SE)	The pathway analysis of diffuse emissions is driven by increased concentrations in Stockholm's receiving sediments.	(Sörme and Lagerkvist 2002; Sörme et al. 2001b)
	Urban catchment (UK)	Water quality is assessed by diffuse emissions entering waste water systems.	(Rule et al. 2006)
	Sofia (BG), New Heaven (USA)	Cu discard, reuse and recovery fluxes and rates are compared between two cities.	(Dimitrova et al. 2007)
	Villach (AUT)	The fate of diffuse emissions is determined in order to assess environmental risks.	(Rebernig 2007)
End-of-Pipe plants	Vienna (AUT)	- Waste water treatment plant: Heavy metal flow ratios on plant level demonstrate the separation efficiency. High separation rates improve environmental performance.<br>- Incinerator: Monitoring of heavy metal in residues reveals the temporal evolution of concentrations in household waste.	(Kroiss et al. 2008; Morf and Taverna 2006)
	Hinwil (CH)	Incinerator: Residues were analyzed in order to estimate recovering potential of precious metals and rare earth elements.	(Morf et al. 2013)
Urban Soil	Edinburgh, Dundee (UK)	Historical early warning of slow-poisoning of urban soils.	(Purves 1966)
	Oslo (SE)	Urban transactions are used to demonstrate the influence of urbanization of chemical soil quality.	(Reimann et al. 2011)
	Vienna (AUT)	Estimation of geogenic background values (relevant for legislation) based on geochemical patterns in urban soil.	(Pfleiderer 2011)
	Taipei (TW)	Identification of toxic contaminants in urban top soil layers.	(Jien et al. 2011)

2. MATERIAL AND METHODS

2.1 City selection and city characteristics

Table S2: City parameters

Parameter	Unit	Vienna 2008	Taipei 2009
Inhabitants	capita	1,674,909	2,607,428
City area	hectare	41,487	27,180
Population Density	capita/hectare	40	96
Gross Regional Product (GDP)	Euro/capita	43,900	34,800

2.2 Substance flow modeling

2.2.1 Model development

Figure S1 displays the generic stock and flow chart.

Figure S1: Generic Cu flow model on 1st level. It covers 9 city internal processes of which 6 represent mainly anthropogenic activities (dark grey boxes) and 3 stand for environmental media (light grey boxes). Exterior processes are splitted in the supply and export as well as receiving waters in the hinterland. Regarding nomenclature, the flow acronyms refer to the type of flow (first three letters), to the source process (second three letters and to the sink process (last three letters). The stock acronyms refer to the type of stock only.

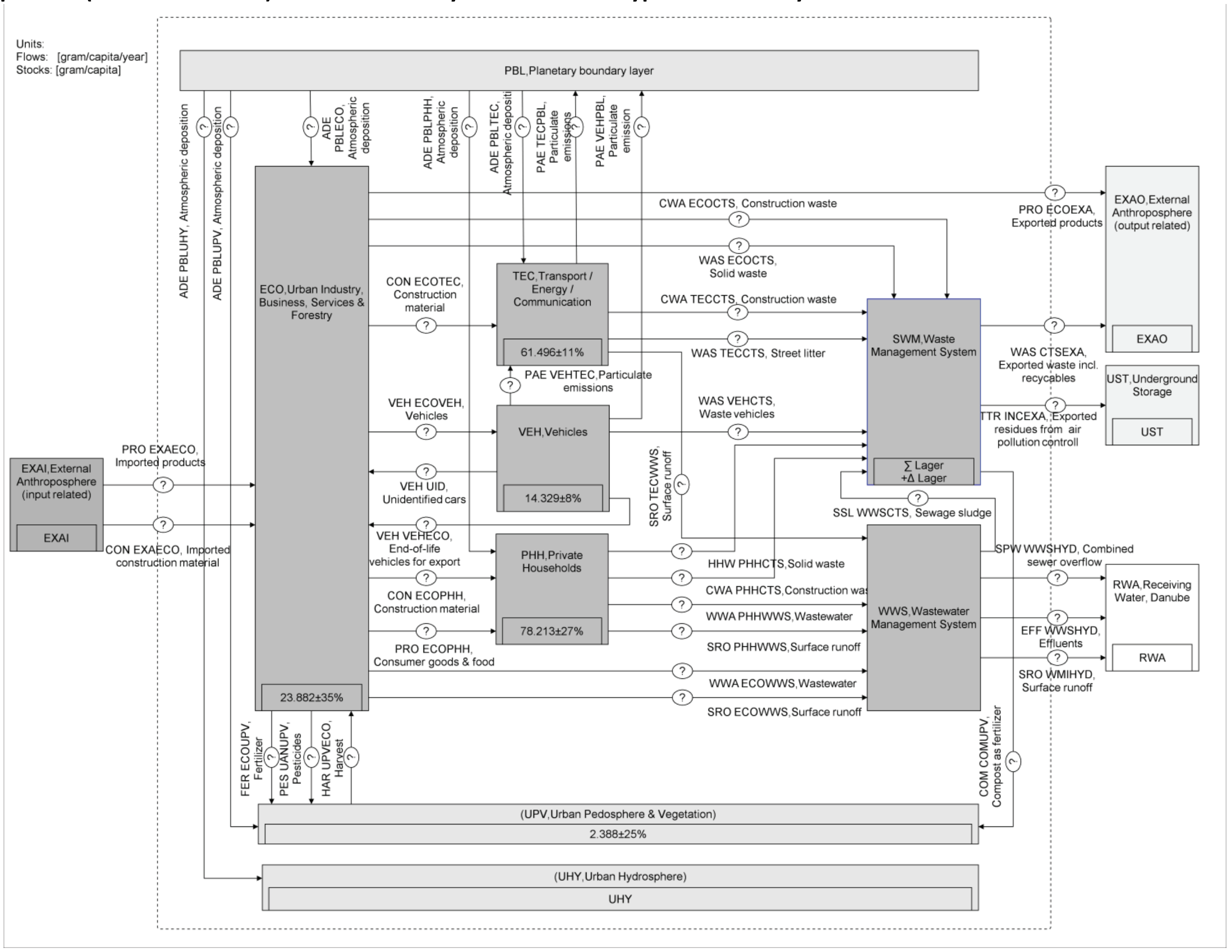

Figure S2: Generic Cu flow model on 2nd level. It disaggregates the "Waste Management System".

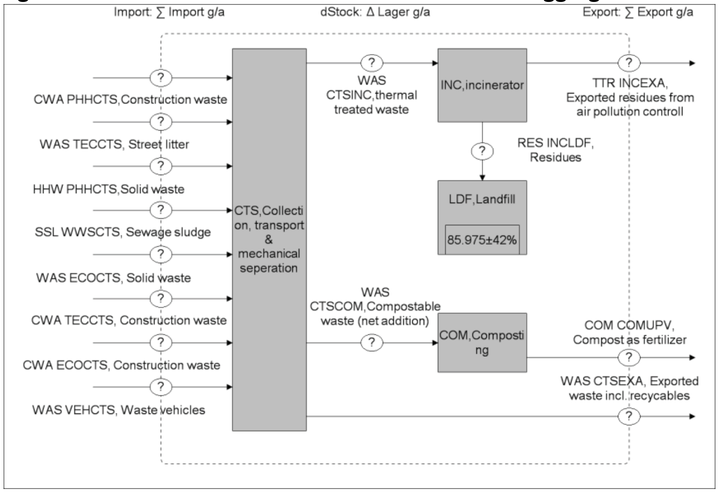

2.2.2 Model equations and data acquisition

This section provides a compilation of flow and stock calculations as well as insights into data acquisition. Additionally, we documented the mathematical model equations, input parameters, uncertainty classification and references for Vienna p. 15, ff. and background data for Taipei on p. 19, ff.

2.2.2.1 Flows & stock description for Vienna and Taipei

2.2.2.1.1 Atmospheric Deposition (ADE PBLUHY, ADE PBLUPV, ADE PBLECO, ADE PBLPHH, ADE PBLTEC)
Urban areas are affected by depositions caused by fireworks, fossil fuel burning, break and catenary wear.

In Vienna, total deposition rate was measured with an rate of 148,7 g Cu/ha/yr on arable land close to Vienna (Spiegel 2003), the dry deposition was measured in town with an average rate of 7,7 g Cu/ha/yr (Kalina et al. 2000). City wide extrapolation on an area base results about 6.6 t Cu/yr, from which 3.5 tons are allocated to building area, 2.8 t Cu/yr to green space (ADE PBLUPV), 0.3 t Cu/yr to open water bodies (ADE PBLUHY) and 0.9 t Cu/yr to traffic surfaces (ADE PBLTEC).

In Taipei, one research was conducted that sampled and analysed the content, source, and transportation mechanism of metals in atmospheric deposition. Dry deposition rate was 3.8 mg/m^2/yr). It was related to public facilities surface (ADE PBLTEC) as 0.3 t Cu/yr, private households (ADE PBLPHH) 0.2 t Cu/yr, open water bodies (ADE PBLUHY) 0.1 t Cu/yr, and 0.3 t Cu/yr to green space (ADE PBLUPV).

2.2.2.1.2 Products (PRO EXAECO, PRO ECOPHH)
The import of products stands for consumer goods and pre-products. Both are finally consumed or processed by trade and skilled labor.

In Vienna, we have chosen four main products groups that ended up in the urban production sector or final consumption. (1) Cu containing goods like sheet metals, wires, cable pies and pre-products were taken from a national Cu flow study (Daxbeck et al. 2006). To allocate the flows on different sectors (buildings, infrastructure, production industries), we used global market shares of specific Cu products (ICF 2001). The number of employers in the construction and production sector (Hauptverband der österreichischen Sozialversicherungsträger SV 2011; Statistik Austria 2009c) were used as proxy to downscale from the national to the urban level. Therefore, 4,600 t Cu/yr are allocated to the production industry and is part of the product flow (PRO EXAECO). 11,800 t Cu/yr were allocated to the construction sector (follow sec. 2.2.2.1.3). (2) Electrical and electronic appliances (EEA) are monitored when they placed on the market and when they turn to waste. To estimate the flow, we used the difference of imports and exports on EEA from the regional, mass based trade statistic (Magistratsabteilung 05 2011) and a mean Cu content based on a

basket of 11 representative goods (Truttmann et al. 2005; Oguchi et al. 2011; Hausmann 2005; Wittmer 2006). To estimate the allocation, we used the national sale statistics for household consumption, the population ratio as proxy for downscaling to the regional level (EAK 2009). This resulted about 1,500 t Cu/yr for PHH and 500 t Cu/yr for ECO, respectively. (3) Vehicles were calculated in sec. 2.2.2.1.4 and resulted 2,200 t Cu/yr (VEH ECOVEH), and (4) Food contains essential Cu for human dietary and varies with the type of food. To estimate the flow, we used sixteen food categories (grain, oilseeds, and so on) from the Viennese trade statistic (Magistratsabteilung 05 2011) and given Cu concentration from the National Nutrient Database (USDA 2011). Therefore, about 2 t Cu/yr were imported with food and fully allocated to PHH.
Totally, 8,800 t Cu/yr are imported via products (PRO EXAECO) of which 1,500 kg Cu/yr (PRO ECOPHH) enters the PHH as EEA and food.

In Taipei, top-down and bottom-up approaches were adopted to estimate of Cu in products. Since “import” generally happens at a national level, there was no urban statistics of imported goods into the city. Hence, the proxy of total retail sales of consumer goods and areas of factories were used to downscale to the urban level for the imported products (PRO EXAECO), as 16,000 t/yr of Cu contained.
Because there is lacking statistics of products consumption within the city, we used data of recycling to estimate backward. In Taiwan, people can recycle household products with certain recycling fee as feedback. Therefore, bottom-up method was adopted to estimate the quantity of consumer goods, with the assumption that Taiwanese purchase a new product when they discard the old one. In terms of Cu-based products, two categories were selected: Waste Electrical and Electronic Equipment (WEEE) and Waste Computer Appliances (WCA). We took statistics from Recycling Fund Management Board, which is an official organization in charge of executing the recycling fund system. Estimation showed that around 5,000 t Cu/yr was consumed in household commodities (PRO ECOPHH).

2.2.2.1.3 Construction material (CON EXAECO, CON ECOTEC, CON ECOPHH, CWA ECOCTS, CWA TECCTS, CWA PHHCTS, CWA CTSLDF)

The construction sector heavily uses Cu for coatings, plumbing equipments and telecommunication and electricity grids in buildings.

In Vienna, the imported Cu as construction material including pipes for plumbing and heating, sheets for roof and outdoor applications as well as cables and wires like building wires for electric currents and telecommunications. As calculated in sec. 2.2.2.1.2, 11.800 t Cu/yr (CON EXAECO) enter the construction sector, of which 9,400 t Cu/yr accumulates in buildings and 2,400 t Cu/yr infrastructure (CON ECOTEC). Specific land use categories were used to add 6,600 t Cu/yr to the PHH (CON ECOPHH) and 2,800 t Cu/yr to industrial and business buildings.
If it comes to construction waste, the total flow was categorized in three groups: (1) Demolition Material amounted 1.8 Mio t/yr of which the Cu fractions are mostly unknown. So, we picked out the top 5 construction waste flows from the regional waste statistic (Wiener Umweltschutzabteilung MA22 2011) and multiplied them with corresponding Cu contents from literature (Brunner and Stampfli 1993; Schnöller et al. 2010; Arx 2006). So, 800 t Cu/yr were estimated in demolition material. Allocation of flows to the waste generators PHH, ECO and TEC was done by a sector based consumption ratio and the land use ratio. Direct disposals were neglected after the economic benefit of recycling omits landfilling. (2) Cu Scrap was collected with an rate of 3,200 t Cu/yr (Wiener Umweltschutzabteilung MA22 2011) and is estimated to be fully recycled. Allocation followed the same routine as for the demolition material. (3) Collected cables were explicit recorded in the local waste statistics. Hence, we used measured Cu contents in cables (Skutan 2008) and the same allocation proxy as for the demolition material. The total construction waste flows were 3,000 t Cu/yr from private households (CWA PHHCTS), 1,300 t Cu/yr from industry and business (CWA ECOCTS) and 700 t Cu/yr from technical infrastructure (CWA TECCTS).

In Taipei, we determined the imported construction material from infrastructure ad buildings import in Taiwan. The economy and transportation budget for infrastructure and the newly built floor area for

buildings were included to downscale statistics from Taiwan to Taipei. The two categories contributed to 45.3% of the total Cu consumed in Taipei which was 13,000 t/yr (CON EXAECO).

We allocated the imported construction material to construction material consumption in economic sectors, private households and infrastructure by the proportion of land-use area. Cu consumed in households and infrastructure was then estimated to be 8,000 t/yr (CON ECOPHH) and 4,000 t/yr (CON ECOTEC), respectively.

For construction waste, construction and demolition waste (C&D waste) and total demolished floor area of the year were considered. Allocation for households, infrastructure an economy sectors were determined by the corresponding area of land-use in Taipei. Results show that Cu in construction waste are 150 t/yr from private households (CWA PHHCTS), 50 t/yr from infrastructure (CWA TECCTS) and 3 t/yr from economy sectors (CWA ECOCTS). The relatively low amount of construction waste produced from infrastructure and economy sectors may be due to the longer lifespan of the infrastructure and buildings.

2.2.2.1.4 Vehicles (VEH ECOVEH, WAS VEHCTS, VEH UID, VEH VEHECO)

Cu is extensively used in vehicles and rolling stocks. It is part of electrical components, heat transfer devices, and bronze sleeve bearings. Hence, countless fittings, fasteners, and screws are made from brass (European Copper Institute (ECI) 2011).

In Vienna, the modal split in the traffic sectors allocates 1/3 of all trips to public transport, 1/3 to private cars and 1/3 to non motorized traffic like cycling and walking. So, private and public transport fleets are of equal importance for Cu calculations. To estimate the inflow, we used official registration statistics for vehicles like cars, busses, lorries and motor bikes (Statistik Austria 2008), the rolling stock alteration of the public transport provider (Lebhart 2010), and the and the average Cu content per vehicle found in the literature (European Copper Institute (ECI) 2011; Bertram et al. 2002; Struckl 2007). This results an inflow of 2,200 t Cu/yr (VEH ECOVEH). The total outflow has not been statistically reported. So, the whereabouts of EOL cars can't be determined at full scale. We carried out a national car balance in order to estimate the number of unidentified cars. Hence, the Viennese car ownership of 15.3% acts as proxy for regional scaling (Statistik Austria 2008) and an average Cu content per car was used to estimate Cu flows. This results an outflow of 880 t Cu/yr of which 240 t Cu/yr enter a shredder (WAS VEHCTS), 140 t Cu/yr t are legally exported to foreign countries (VEH VEHECO) and 500 t Cu/yr remain unidentified in terms of their whereabouts (VEH UID).

In Taipei, private vehicles dominate transportation, especially the sedans and scooters. Since statistics of newly sold vehicles were absent, Cu estimation was top-down from the national data. Vehicles which are ready to be in use were assumed to be the sales volume of cars and scooters/motorcycles of the study year. By the Taipei-Taiwan ratio of newly registered vehicles and Cu content 1.4% (Bertram et al. 2002), sales volumes in Taipei was estimated to be 500 t/yr of Cu (VEH ECOVEH).

We assumed that all end-of-life vehicles (ELV) were treated as recyclable waste. Since data of recycled ELV in Taipei was lacking, we used statistics of recycled ELV in Taiwan and Taipei-Taiwan ratio of newly registered vehicles to top down the estimation, as a result of 200 t/yr of Cu in waste vehicles (WAS VEHCTS).

Amount of registered vehicles difference between 2008 and 2009 was assumed to be the exported ELV, since the change of ELV was unknown and unclear. Hence, 90 t Cu/yr in ELV (VEH VEHECO) was estimated to leave the city boundary.

2.2.2.1.5 Particulate emissions (PAE VEHTEC, PAE VEHPBL, PAE TECPBL)

Particulate emissions are driven by wearing, weathering and corrosion processes. Diffuse emission patterns are characteristic for urban areas. They end up in urban soil and surface runoff.

In Vienna, we considered three main emission sources. First, brake wears from low duty vehicles was estimated according to the fleet mileage (Holzapfel and Riedel 2011), average total wear rates (Winther and Slento 2010) and Cu concentrations in brake linings representing the Austrian fleet (Figi et al. 2010). Finally we used transfer coefficients from the Netherlands National Emission Inventory (Hulskotte et al. 2006; Oonk et al. 2005) in order to examine the flows towards ambient air, road surfaces and vehicle depositions.

Second, brakes and wheels from rolling stock (trams, trains) emit Cu too. To estimate the flows, we processed the individual network lengths (ÖBB-Holding AG 2008; Wiener Linien 2008), normalized Cu abrasion rates from railways (Burkhardt et al. 2005) and the transfer coefficients from individual sources to ambient air, railroad, surrounding and vehicle deposits (Müller et al. 2008). Third, catenary wear contains 99.5-99.9% Cu on a mass basis. To estimate the wear entering urban sinks, we used the track lengths, corresponding cross sections at the time of installation and replacement, an average lifetime of 40 years and transfer coefficients were derived from a SFA study that balanced Cu flows on 1 kilometer railroad (Müller et al. 2008). Fourth, flows from roofs were estimated according to (Odnevall Wallinder et al. 2007) with a rate of 1.5 g $Cu/m^2/yr$. The Cu roof area was gathered from local tin smiths (Wocilka and Höfner 2011), the total roof area and average roof slope was delivered by city authorities who once plotted the inclinations for estimating solar energy potentials (Kubu 2011), the regional precipitation rate (Lebhart 2010), the SO2 concentration in precipitation was equally set with monitoring data in ambient air (Augustyn et al. 2010), and the pH value with 5.
Allocating the single results that a) the vehicles release 5 t Cu/yr to the ambient air (PAE VEHPBL), 2.2 t Cu/yr occur as road debris (PAE VEHTEC), and b) the catenaries release 0.6 t Cu/yr to ambient air (PAE TECPBL), 2.0 t Cu/yr to surrounding urban surfaces like roads and facades. Cu deposits on vehicles were not taken into further account.

Taipei City is a rather young Asian urban system and materials used in roof are different from those in Europe. Cu flows of trains, trams and roofs were not taken into account. Airborne particulates from light and high duty vehicles were considered in break wear estimation, with mileage (Department of Statistics 2007) and the wear rate which was the same as in Vienna. Mass of Cu in tire wear were estimated through the top-down data of registered vehicles in Taipei, the tire wear factor, and Cu content in the tire wear emission. Around 6 t Cu/yr were emitted due to break wear and tire wears (PAE VEHPBL).

2.2.2.1.6 Waste Water flows (SRO ECOCTS, SRO PHHWWS, SRO TECWWS, SRO WMIHYD WWS ECOWWS, WWS PHHWWS, SSL WWSCTS, SPW WWSHYD, EFF WWSHYD)

Waterborne Cu flows receive inputs from diffuse emissions, surface runoff and grey water from private households, Cu processing industry and skilled labor.

In Vienna, the majority of collected water flows entered the mixed sewer network and the waste water treatment plant (WWTP) downstream. Those flows become part of grill material, sewage sludge or effluent. Combined sewer overflow enters the receiving water without treatment. The separated sewer network takes up parts of the surface runoff and transports it to the receiving water. We established a separate SFA model (Figure S3) in order to balance waterborne Cu flows.

- Measured flows were available for the WWTP (Kroiss et al. 2008). Therefore, 16.2 t Cu/yr enter the plant of which 13.6 t Cu/yr (SSL WWSCTS) are transferred to the sewage sludge, 1.6 t Cu/yr to the effluent (EFF WWSHYD) and 1.0 t Cu/yr to the grill material.
- Diffuse emissions enter urban surfaces via surface runoff. They calculation of particulate emissions from the transport sector (low duty cars, trams and trains, catenary), building sector (Cu roofs) and atmospheric deposition is described at sec. 2.2.2.1.5. The surface runoff from the transportation grid covers brake & tire wear and catenary with an amount of 3.3 t Cu/yr (SRO TECWWS). The surface runoff from PHH carries atmospheric deposition and roof runoff with a flux rate of 4.0 t Cu/yr (SRO PHHWWS). The surface runoff from ECO covers 2.2 t Cu/yr (SRO ECOWWS). The ratio of separated and mixed sewer network length of 19:81 (Lehmann 2011) is used as proxy to allocate the surface runoffs to the two sewer types. 1.8 t Cu/yr enter the separate sewer system which transports the Cu to the receiving water. 8.9 t Cu/yr enter the mixed sewer system which of 50% enter receiving water as spillover (Fenz 1999) with a flux rate of 3.8 t Cu/yr (SPW WWSHYD).
- Two point sources are relevant. (1) Waste water in PHH covers the final use of tap water as well as Cu from anthropogenic activities. (1.1) Viennese tap water covers the geogenic Cu content and Cu corrosion from pipes. Data on local water consumption statistics excluding the losses (Daxbeck et al. 1996; Tomenendal 2011) and measurements of Cu concentrations at the network endpoints in

households (Magistratsabteilung 31 2008) result a flux rate of 2.3 t Cu/yr. (1.2) Human off-flows like feces, urine and skin particles result about 760 mg Cu/cap/yr (I C Consultants Ltd 2001; Lampert et al. 1997). Other emission sources like residues from food preparation, washing dishes, toilet papers, washing clothes and cleaning activities count for 843 mg Cu/cap/yr (Baccini et al. 1993; I C Consultants Ltd 2001). The multiplication with Viennese population size results 2.7 t Cu/yr. In total, the flux rate is 5.0 t Cu/yr (WWS PHHWWS). (2) Waste water data from business and industries were restricted for access. As a consequence, we calculated the flow by stressing the mass balance principle with a flux rate of 7.4 t Cu/yr (WWS ECOWWS).

Figure S3: Cu in urban water flows (Diffuse emissions = yellow, Point emissions = pink, Outputs from WWS =blue)

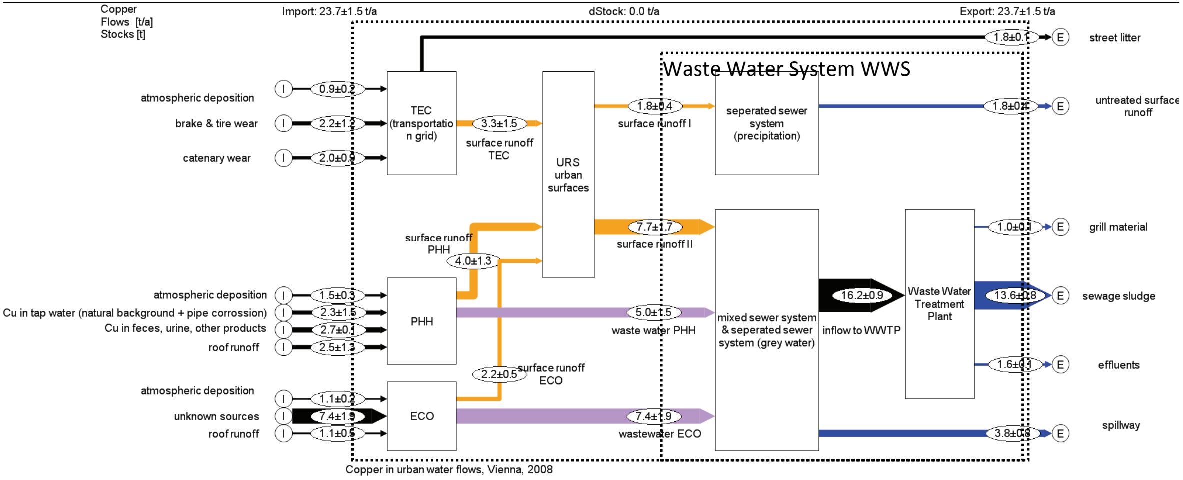

In Taipei, the percentage of houses connected to public sanitary sewers is 100%. There are three wastewater treatment plants in operation for Taipei: Dihua, Neihu, and Bali. Local statistics showed that Cu in untreated and treated wastewater from Neihu WWTP were not detectable (< 0.02 mg/L). To estimate the flow, we set the detection limit of 0.02 mg Cu/l as maximum concentration. The estimation turned out to be 9.8 t/yr (WWA PHHWWS) and 2.8t/yr (TWA WWSHYD). By the ratio of different land uses, Cu in surface runoff was 0.02 t/yr (SRO ECOWWS), 0.5 t Cu/yr (SRO PHHWWS) and 0.4 t Cu/yr (SRO TECWWS). Cu in sewage sludge was then estimated to be 60 t Cu/yr (SSL WWSCTS). To estimate the flow, we used the sewage sludge flow with 65,700 tons (sum of Bali, Dihua, and Neihu sewage farms) and Cu concentrations with 887 ppm from Bali Sewage Farm (Shih 2007).

2.2.2.1.7 Solid waste (HHW PHHCTS, WAS ECOCTS)

Cu in solid waste streams are much sought after by stockholders in the recycling and recovering business. Due to the economic incentivizes waste collection strategies and technologies that gain valuable metals are continuously improved.

In Vienna, waste from private households, industry and business covered four waste streams: (1) Municipal solid waste and likewise fractions from business and industry was collected by public service providers. It amounts 579,888 t/yr, while recent studies in Austria determine the concentration from 1,800 – 2,200 mg Cu/kg (Skutan and Rechberger 2007). (2) Waste from industry and business contains four industrial waste fractions like waste from punching and planking, Cu chloride, non-ferrous metal scrap and electroplating sludge. To estimate the flow, we used local waste statistics (Wiener Umweltschutzabteilung MA22 2011) and waste specific Cu contents derived from personal correspondence (Daxbeck et al. 2006). (3) Bulky waste composition varies all over Austria due to different collection schemes. In Vienna, about 70.000 t are collected as composite materials, wood and metals (Wiener Umweltschutzabteilung MA22 2011). After the Cu content is varying to a large extent, we used a broad bandwidth from 1,800-3,300 mg/kg (Skutan and Brunner 2006). (4) Waste electrical and electronic equipment (WEEE) covers large apparatuses, fridges, and freezers, monitors, small WEEE and lamps. To estimate the Cu flow from private households, we used collection data from the national coordination center for WEEE and Cu concentrations from a Swiss Cu flow

study (Wittmer 2006). Those from business were not exclusively reported for Vienna. Therefore, the national amount was downscaled by the ratio of WEEE from private households to total WEEE flow in Vienna. Summarizing, about 1,700 t Cu/yr were generated by households (HHW PHHCTS) and 5,400 t Cu/yr by industry and business (WAS ECOCTS).

In Taipei, household waste includes the trash collected by cleaning squad and by people themselves, as well as the recyclables. Result shows that 1,700 t/yr of Cu contained in household waste (HHW PHHCTS). In Taiwan, industrial waste comprises from industries and service sectors. Since there was nearly no factory in Taipei, industrial waste was presumed to come from service sectors, whose composition is similar to household waste. Because the process ECO was defined as the industrial sector, the amount of industrial waste transported by industrial or waste management institutes to incinerators and landfill sites were considered. Cu concentration in industrial waste was assumed to be as the same as bottom ash and fly ash from incinerators with a rate of 60 t Cu/yr (WAS ECOCTS).

2.2.2.1.8 Exported products (PRO ECOEXA)

Exported products stand for valuable goods which cross the city boundary.

In Vienna, exported products cover three sub-flows. First, the exported vehicles are traded as goods and were estimated to be 140 t Cu/yr (VEH VEHECO). Second, the number of unregistered EOL vehicles was assumed to be exported with 500 t Cu/yr (VEH UID). Third, fabricated goods from the production industry lack of sound data after the import/export statistics are based on the headquarter approach instead of a territorial allocation. So, we estimated 2,000 t Cu/yr with the help of import/export statistics and subtracted residues from production. In total, 2,600 t Cu/yr leave Vienna in a physical product form (PRO ANTEXA).

In Taipei, the same estimation assumption was made as in Vienna, and products exported from Taipei City were estimated to be 8,000 t Cu/yr.

2.2.2.1.9 Exported waste including recyclables (WAS CTSEXA)

Exported waste flows are directed to recycling or disposal facilities out of the city. They cover valuable Cu which is recovered as well unrecovered Cu in residues from incineration.

In Vienna, we combined the generated waste flows, individual information regarding the whereabouts in recycling facilities and specific Cu contents. The flow is summarized by individual flows like recyclables within construction waste, waste from industry and business, and recycled EOL vehicles to an extend of 11,100 t Cu/yr (WAS CTSEXA).

Taipei City incinerated household waste from Keelung City too and stored the bottom ash temporarily during the period of constructing new landfill in Keelung. Likewise, Keelung City would provide landfill service of waste from Taipei City and temporary depository fly ash monolith. The temporary repository of bottom ash and fly ash will be sent back to the original city after the new landfill is constructed. Statistics of Taipei City showed that in 2003 total weight of bottom ash conveyed to Keelung City was 96,000 tons. We also assumed that ELV, scrap, and recyclables were included in exported waste, result in total 2,700 t Cu/yr (WAS CTSEXA).

2.2.2.1.10 Incineration: Mixed waste, exported residues for underground storage, residues, disposable waste (WAS CTSINC, TTR INCEXA, RES INCLDF)

Waste incinerators are used to treat household and toxic industrial waste by temperatures up to 1,200 °C. While microorganisms like bacteria, fungi and virus as well as organic compounds are mineralized, heavy metals like Cu are transferred to bottom ash and APC residues. The whereabouts of residues depends on national polices and varies from bottom ash as construction material to underground disposal for filter APC residues.

In Vienna, four plants secure the treatment of household waste, residues from mechanical sorting, bulky waste fractions, street litter and some minor waste fractions with an amount of 600,000 t/yr. To estimate the plant load, we used Cu concentrations for six different waste flows that stem from plant specific monitoring reports and waste flow measurements (Boller 2002; Skutan and Brunner 2006; Skutan and Rechberger 2007; Umweltbundesamt 2000; Arx 2006; Hausmann 2005). Therefore, about 1,140 t Cu/yr enter the incinerators (WAS CTSINC). Public secondary waste flow data (Wiener Umweltschutzabteilung MA22 2011) and measured Cu concentrations in a representative Austrian waste to energy plants (Skutan and Rechberger 2007; Taverna et al. 2011) result 990 t Cu/yr in bottom ash (RES INCLDF) and 16 t Cu/yr in APC residues (TTR INCEXA). The bottom ash is used as aggregate to construct concrete blocks for landfill stabilization that ends up at a Viennese landfill. APC residues are heavily contaminated with salts and heavy metals. In 2008, they were exported to Heilbronn, an underground storage facility in Germany.

In Taipei, three incinerators operate for Taipei: Beitou, Neihu, and Muzha. According to local statistics, waste transported into incinerator was 640,000 ton in 2009. Cu content was estimated from the Cu concentration in fly ash and bottom ash. Cu in municipal solid waste (MSW) was 200 t/yr (WAS CTSINC). Residues from incinerators consist of fly ash and bottom ash, which contained 200 t/yr of Cu (RES INCLDF). Due to the policy “Zero Waste” of the local government, the waste amount in Taipei has decreased, and no statistics showed that residues from incinerators were exported from the city. Hence, Cu in exported residue for underground storage is zero (TTR INCEXA) in Taipei’s case.

2.2.2.1.11 Composting: Compostable waste, residues, compost as fertilizer (WAS CTSCOM, RES COMCTS, COM COMUPV)

Composting degrades biogenic waste in terms of volume and mass. Quality proven compost can be used as nutrient supplier for plants and soil improver.

In Vienna, one compost plant has been operated. The inputs stem from separated biomass collection and cover materials such as greencut and uncooked vegetables from households. To estimate the Cu flows throughout the plant, we used monitored bulk flows, and derived average dry matter contents as well as Cu concentrations from the literature. The inflow covers compostable raw materials and residues from anaerobic digestion in a biogas plant (Magistratsabteilung 48 2009). Data lacks prevented full Cu balance on plant level. So we set the output as net addition to plant. We assumed a dry matter ratio of 30% and a Cu concentration of 83 mg/kg dry matter and results 1.4 t Cu/yr (WAS CTSCOM). About 33.000 t valuable compost is used for land applications in Vienna (Republik Österreich 2001b; Weinmar 2011). Legal Cu concentrations for high quality compost A+ was set with 70 mg/kg dry substance (Republik Österreich 2001a) and resulted 1.4 t Cu/yr (COM CTSCOM = COM COMUPV).

In Taipei, incinerator plants in Taipei have auxiliary spaces to process compost, which has nothing to do with incinerators themselves. Uncooked food waste scraps are collected to incinerator plants and through the process of dehydration, fermentation, etc., they become compost as product. People or companies can take the compost if they need. Since there are few livestock industries or agricultural activities in Taipei, the compost here was assumed to be the treated uncooked food waste without residues. Due to limited data, the official statistics of uncooked food waste scraps in Taipei City and compost from Muzha Refuse Incineration Plant were adopted to estimate total compost in Taipei, which included 0.04 t Cu/yr (WAS CTSCOM, COM COMUPV).

2.2.2.1.12 Pesticides (PES ECOUPV)

The agricultural sector applies Cu as purposeful fungicides and bactericide on fruits and vegetables. The utilization of Cu dates back to beginning of the 19th century and was known as “Bordeaux-Brühe” It is a mixture of chalk and aquatic Cu-sulphate-dissolution and bans the evolution of mildew. In 2009, the European Commission decided to constrain Cu form agricultural appliances after 2016 (Berger et al. 2011). Member states are enforced to establish national risk assessments in order to regulate Cu use.

In Vienna, the flow was calculated based on data on Austrian pesticide and plant protection product use (Berger et al. 2011) for wine, fruits and vegetables & crops and Viennese agricultural land use areas. In total, about 2.4 t Cu/yr are released by pesticides (PES ECOUPV).

In Taipei, there are barely agricultural activities; the agricultural area is 5.31 km^2, which is around 2% of total area of the city. Hence, estimation of Cu in pesticides was not included for Taipei's case.

2.2.2.1.13 Fertilizer (FER ECOUPV)

Manure, mineral fertilizer and harvest residues are common fertilizer inputs into agricultural soils. Especially manure is known to have a significant Cu concentration. Concentrations vary from 40-300 mg Cu/kg manure depending on the type of animal and foodstuff (Zethner et al. 2007). As an example, Cu is given to piglets in high doses to protect them from various diseases in the first weeks. Mineral fertilizer and harvest residues cover minor Cu as trace element.

In Vienna, the agricultural sector plays a minor role in terms of land use and production rates. About 15% of urban area is used to produce working animals, crops, wine and vegetables for local supply primarily. The Cu release was estimated by multiplying normalized Austrian and German Cu inputs (Fricke and Höhl 2000; Umweltbundesamt 2001; Zethner et al. 2007; Berger et al. 2011) with agricultural area classified into viticulture production, fruit production, and agriculture & horticulture (Fitzthum 2009). In total about 2.2 t Cu/yr are released by fertilizer applications (FER ECOUPV).

In Taipei, there are barely agricultural activities; the agricultural area is 5.31 km^2, which is around 2% of total area of the city. Hence, estimation of Cu in fertilizer was not included for Taipei's case.

2.2.2.1.14 Technical Infrastructure (TECstock)

Cu is mainly used for electricity transmission and information transfer.

In Vienna, three types of networks are taken into account. First, the electricity grid covers ~3.700 km of overhead lines and ~ 22.300 km of underground cables (Wien Energie 2010). The stock was estimated based on voltage classes, cable lengths, cross sections and specific masses according to the Swiss network (Wittmer 2006). In total, about 89,200 t Cu provide the public energy supply. Second, Cu in the telecommunication network is estimated by a proxy based on the number of business units. Combining data from Australia and Sydney (van Beers and Graedel 2007) with Viennese business statistics resulted about 13,300 t Cu. Third, catenaries supply the 2nd largest tram network worldwide as wells as national and local railway tracks with electricity. The Cu stock of 1,000 t is estimated based on the track length, cross sections, Cu content, and lifetime data. Summarizing, the infrastructure covered about 103,000 t Cu. In reality, the stock is larger than estimated due to Cu cables in subway lines and power transfer stations which were not taken into account.

In Taipei, the electricity transmission system and telecommunication system were taken into account. The electricity grid included 2,100 km overhead power lines and 310 km underground power lines (Lin 2003). For the telecommunication network, estimation was made according to case of Australia and statistics of household and business number in Taipei which gave a result of 24,000 t Cu. Cu stock in infrastructure was aggregated as 32,000 t Cu. Since Cu stock in cables and wires of MRT (Mass Rapid Transit) was not included, the actual value stock maybe larger than the estimated one.

2.2.2.1.15 Economy and private households (ECOstock, PHHstock)

The process economy and private household covers stocks like roofs, water pipes, heating systems, telecommunication and electricity networks as well as consumer goods.

In Vienna, the building structure and housing technology is comparable to Swiss standards. Using per capita data from Switzerland (Wittmer 2006) result about 134,000 t Cu in buildings. The land use area is used as proxy to allocate 94,000 t Cu to private households and 40,000 to economy. Cu in consumer goods like washing machine, dryer, electronics equipment and minor items like keys and coins stands for 37,000 t Cu. They were allocated to private households.

In Taipei, we selected items based on products listed in recycling fund system to estimate Cu stock in consumer goods. Assumption was made that those Cu-contained products included in recycling fund system were those comparatively highly used in households in Taiwan. The lifespan of different products were presumed to be uniform distribution. These consumer goods were either electrical and electronic appliances (EEA) or computer appliances. Five items for Cu stock calculation were color TV, air conditioner, washing machine, laptop, and desktop computer. With numbers of hundred households in Taipei as 9,628.31 in 2009, Cu consumed annually in household appliances and computer appliances in Taipei City were 17,000 t Cu.
In economy sectors (ECO), building materials were assumed to be the stocks in it while consumer products were all input to private households (PHH). Electricity and telecommunication wire and cables were taken into account for Cu stock of buildings. In this study, the average length of in-use line in a case study in Tainan (Ou et al. 2007) and the total floor area in Taipei were integrated to do estimation. The Tainan case was reasonable and suitable for estimating because its structure and space usage are common around Taiwan, and hence the case could represent the general housing in Taiwan as well as in Taipei. We used ratio of land-use to allocate Cu stocks of buildings to ECO and PHH. The result shows that 200 t Cu existed in the form of building materials in ECO.

2.2.2.1.16 Vehicles (VEHstock)

The vehicle pool covers private and commercial wheelers as well as rolling stocks.

In Vienna, the calculation is based on the number of vehicles (Statistik Austria 2008; Wiener Linien 2009) and corresponding Cu contents from literature (Bertram et al. 2002; European Copper Institute (ECI) 2011; Hoock 2008; Struckl 2007). The private and public owned fleet consist registered motorized vehicles like cars, lorries and busses as well as rolling stock such as subways and trams. In total, the Cu stock in the transport sector covers 24.000 t Cu.

In Taipei, the estimation included the number of registered cars, buses and scooters in operation. 12,000 t Cu was estimated to be placed in vehicles.

2.2.2.1.17 Urban Soil (UPVstock)

Urban soil covers Cu, which compromises geogenic backgrounds and former anthropogenic inputs.

In Vienna, soil sampling data were reported by the City Authority. We printed a box plot including the detected Cu concentrations in parks and playgrounds. Therefore, about 46 mg Cu/kg soil were multiplied with the area of green space and a soil depth of 30 cm. This results about 4,000 t Cu in urban soil (UPVstock).

In Taipei, there is no local report of the soil sampling data, and hence estimation was made according to EPA public health declaration that Cu concentration in soil generally ranges from 2 to 250 ppm. With the area of green space of Taipei City and 30-cm depth of soil, Cu stock in urban pedosphere was obtained to be 830 t (UPVstock).

2.2.2.2 Vienna – List of mathematical model equations and input parameters

Table S3: Model Equations and flow results

	Flow Acronym	Description	Equation	Expected Value [t/yr]	Deviation [t/yr]
01	ADE PBLECO	Atmospheric Deposition ECO	Dep * A_{ECO}	1.1	0.19
02	ADE PBLPHH	Atmospheric Deposition PHH	Dep * A_{PHH}	1.5	0.26
03	ADE PBLTEC	Atmospheric Deposition TEC	Dep * A_{TEC}	0.9	0.15
04	ADE PBLUHY	Atmospheric Deposition UHY	Dep * A_{UHY}	0.3	0.05
05	ADE PBLUPV	Atmospheric deposition UPV	Dep * A_{UPV}	2.8	0.48
06	COM ANTUPV	Compost, applied on land	m_{comp} * w_{comp} * c_{comp}	1.4	0.24
07	CON ECOPHH	Construction Material	$\mathbf{Cons_{AUT}}$ * $\mathbf{Proxy_{Market\ Share}}$ * $Proxy_{Employees,\ bui}$ * $Proxy_{area}$	6,567	938
08	CON ECOTEC	Construction material	$\mathbf{Cons_{AUT}}$ * $\mathbf{Proxy_{Market\ Share}}$ * $Proxy_{Employees,\ bui}$	2,448	376
09	CON EXAECO	Imported construction material	$\mathbf{Cons_{AUT}}$ * $\mathbf{Proxy_{Market\ Share}}$ * $Proxy_{Employees,\ bui}$	11,817	1,390
10	CWA ECOCTS	Construction waste ECO	DEM+SCR+CAB	1,275	217
		Demolition waste (DEM)	DEM=$\mathbf{Dem}$*$\mathbf{c_{dem}}$ * (1-$Proxy_{area}$)	223	53
		Scrap metal (SCR)	SCR= Scrab*c_{scrabl} * $\mathbf{Proxy_{pre-products,\ scrap}}$ * (1-$Proxy_{area}$)	894	207
		Cables (Cab)	CAB=Cable*c_{cable} * $\mathbf{Proxy_{pre-products,\ cable}}$ * (1-$Proxy_{area}$)	158	37
11	CWA PHHCTS	Construction waste PHH	DEM+SCR+CAB	2,983	509
			DEM=$\mathbf{Dem}$*$\mathbf{c_{dem}}$ * $Proxy_{area}$	522	123
			SCR= Scrab*c_{scrabl} * $\mathbf{Proxy_{pre-products,\ scrap}}$ * $Proxy_{area}$	2,092	486
			CAB=Cable*c_{cable} * $\mathbf{Proxy_{pre-products,\ cable}}$ * $Proxy_{area}$	369	86
12	CWA TECCTS	Construction waste TEC	DEM+SCR+CAB	682	107
			DEM=$\mathbf{Dem}$*$\mathbf{c_{dem}}$	90	14
			SCR= Scrab*c_{scrabl} * $\mathbf{Proxy_{pre-products,\ scrap}}$	249	62
			CAB=Cable*c_{cable} * $\mathbf{Proxy_{pre-products,\ cable}}$	343	86
13	EFF WWSHYD	Effluents	$m_{cu,\ inflow}$ * $\mathbf{TC_{cu,\ WWTP}}$	1.6	0.12
14	FER ECOUPV	Fertilizer	$\mathbf{A_{agr}}$ * $\mathbf{m_{fertilizer}}$	2.2	0.3
15	HAR UPVECO	Harvest	$\mathbf{m_{uptake}}$ * $\mathbf{Proxy_{Land\ use,\ agriculture}}$	0.3	0.05
16	HHW PHHCTS	Household waste	MSW+BW+WEE	1,693	100
		Municipal Solid Waste (MSW)	MSW= m_{MSW} * c_{MSW}	1,123	79
		Bulky Waste (BW)	BW=m_{BW} * c_{BW}	170	34
		WEE	WEE=$\mathbf{m_{WEE,\ PHH}}$ * $\mathbf{c_{WEE}}$	400	52
17	PAE TECPBL	Particulate emissions	$\mathbf{l_{train,\ tram}}$ * $\mathbf{A_{CAT}}$ * 1/a_{CAT} * A_{loss} * $\mathbf{TC_{MOV,\ TEC}}$	0.6	0.2
18	PAE VEHPBL	Particulate emissions	LDV+ROL	5.0	2.5
		Low Duty Vehicles (LDV)	LDV=mil * $\mathbf{e_{LDV}}$ * $\mathbf{c_{brake}}$ * $\mathbf{TC_{MOV,\ TEC}}$	4.9	2.4
		Rolling Stock (ROL)	ROL=(l_{train} + l_{tram}) * e_{ROL} * $\mathbf{TC_{MOV,\ TEC}}$	0.1	0.3
19	PAE VEHTEC	Particulate emissions	LDV+ROL	2.2	1.2
			LDV=mil * $\mathbf{e_{LDV}}$ * $\mathbf{c_{brake}}$ * $\mathbf{TC_{MOV}}$	2	1
			ROL=(l_{train} + l_{tram}) * e_{ROL} * $\mathbf{TC_{MOV}}$	0.1	0.6
20	PES UANUPV	Pesticides	$\mathbf{A_{agr}}$* $\mathbf{p_{rate}}$	2.4	0.3
21	PRO ECOEXA	Exported products	VEH VEHECO + VEH UID + FG	2,574	746
		EOL vehicles for export (VEH VEHECO)	VEH VEHECO = car_{dereg_exp} * c_{car}	138	68
		Unidentified Vehicles (VEH UID)	VEH UID = see row 33	498	330
		Fabricated goods from production sector (FG)	FG=Imp_{good} - $Proxy_{exp\_good}$ * Exp_{good}	1,938	666
22	PRO ECOPHH	Consumer goods & food	EEA+FOO	1,484	205
		Electrical and Electronic Appliances (EEA)	EEA = $\mathbf{EEA_{PHH}}$ * $\mathbf{c_{EEA}}$	1,482	205
		Food (FOO)	FOO=$\mathbf{Food}$ * $\mathbf{c_{food}}$	2	0
23	PRO EXAECO	Imported products	CCG+EEA+VEH ECOVEH+FOO	8,827	728
		Cu containing goods (CCG)	CCG=$Cons_{AUT}$ * $\mathbf{Proxy_{Market\ Share}}$ * $Proxy_{Employees,\ ind}$	4,619	625
		Electrical and Electronic Appliances (EEA_{total})	EEA_{total}= $\mathbf{EEA_{PHH}}$ * $\mathbf{c_{EEA}}$ * $EEA_{I/E}$ / $\sum$ $\mathbf{EEA_{PHH}}$	1,969	325
		Vehicles (VEH ECOVEH)	VEH ECOVEH=$\mathbf{VEH}$ * $\mathbf{c_{veh}}$	2,237	185
		Food (FOO)	FOO=$\mathbf{Food}$ * $\mathbf{c_{food}}$	2	0
24	RES INCLDF	Residues from incineration	$m_{bottom\ ash}$ * $c_{bottom\ ash}$	986	170
25	SPW WWSHYD	Combined sewer overflow	$Ratio_{SPW}$ * $Ratio_{SST}$ * (SRO TECWWS + SRO PHHWWS + SRO ECOWWS)	3.8	0.8
26	SRO ECOWWS	Surface runoff ECO	ADE PBLECO + PAE ECOURS	2.2	0.5
		Atmospheric Deposition PHH (ADE PBLECO)	ADE PBLPHH=see row 01	1.1	0.19
		Roof runoff (PAE ECOURS)	PAE PHHURS= $Area_{Roof}$ * $Ratio_{Curoof}$ * r_{roof} * (100-$Proxy_{PHH,\ roof}$)	1.1	0.50

27	SRO PHHWWS	Surface runoff PHH<br>Atmospheric Deposition PHH (ADE PBLPHH)<br>Roof runoff (PAE PHHURS)	ADE PBLPHH + PAE PHHURS<br>ADE PBLPHH=see row 02<br>PAE PHHURS= $Area_{Roof}$ * $Ratio_{Curoof}$ * r_{roof} * $Proxy_{PHH, roof}$	4.0<br>1.5<br>2.5	1.3<br>0.26<br>1.26
28	SRO TECWWS	Surface runoff TEC<br>Atmospheric Deposition TEC<br>Brake & Tire wear on TEC (PAE VEHTEC)<br>Catenary wear on TEC (PAE TECTEC)<br>Street litter (WAS TECCTS)	ADE PBLTEC + PAE MOVTEC + PAE TECTEC - WAS TECCTS<br>ADE PBLTEC = see row 03<br>PAE VEHTEC= see row 19<br>PAE TECTEC= $\mathbf{l_{train,\ tram}}$ * $\mathbf{A_{CAT}}$ * 1/a_{CAT} * A_{loss}* $\mathbf{TC_{MOV,\ TEC}}$<br>WAS TECCTS= see row 40	3.3<br>0.9<br>2.2<br>2.0<br>1.8	1.5<br>0.2<br>1.2<br>0.9<br>0.05
29	SRO WMIHYD	Surface Runoff	SRO TECWWS * (1-$Ratio_{SST}$)	1.8	0.05
30	SSL WWSCTS	sewage sludge	$m_{cu,\ inflow}$ * $\mathbf{TC_{cu,\ WWTP}}$	13.6	1.1
31	TTR INCEXA	Exported APC residues	$m_{APC\ res}$ * $c_{APC\ res}$	16.4	1.3
32	VEH ECOVEH	Vehicles, consumed in Vienna	**Vehicles***$\mathbf{c_{vehicles}}$	2,237	185
33	VEH UID	Unidentified cars	$(Car_{dereg} + Car_{imp_{reused}} - Car_{rereg} - Car_{shred} - Car_{dereg\_exp}) * m_{cu,car}$	498	330
34	VEH VEHECO	EOL vehicles for export	Car_{dereg_exp} * ccar	138	68
35	WAS CTSCOM	Compostable waste (net addition)	m_{comp} * w_{comp} * c_{comp}	1.4	0.3
36	WAS CTSEXA	Exported Waste and Recyclables	$\mathbf{m_{WR}}$	11,135	729
37	WAS CTSINC	Thermal Treated Waste<br>Mixed waste (MW)<br>Sewage Sludge (SSL)	MW+SSL<br>MW=$\mathbf{m_{mw}}$ * $\mathbf{c_{mw}}$<br>SSL=see row 30	1,137<br>1,123<br>13.6	80<br>80<br>1.1
38	WAS ECOCTS	Solid waste ECO<br>Waste from industry and business (WIB)<br>WEE	MSW+WIB+BW+WEE<br>WIB=$\mathbf{m_{wib}}$ * $\mathbf{c_{wib}}$<br>WEE= $\mathbf{m_{WEE,\ PHH}}$ * $\mathbf{c_{WEE}}$ * $Proxy_{WEE,\ ECO}$	5,390<br>5,363<br>27	457<br>457<br>3
39	WAS TECCTS	street litter	m_{street} * c_{street}	1.8	0.05
40	WAS VEHCTS	Waste vehicles	Car_{shred} * ccar	235	39
42	WWA ECOWWS	Wastewater ECO<br>Unknown sources	$m_{cu,\ inflow}$ + SPW WWSHYD - SRO URSMSW – WWA PHHWWS	7.4	1.9
41	WWA PHHWWS	Wastewater PHH<br>Cu in tap water (TAB)<br>Cu in feces, urine, other products (FUO)	TAB + FUO<br>TAB=$m_{tabwater}$ * $Loss_{water}$ * $c_{cu,\ tabwater}$<br>FUO= Cap_{VIE} * (c_{ww} + c_{bf})	5.0<br>2.3<br>2.7	1.5<br>1.52<br>0.11

Note: t = metric tons per year

Table S4: Model Equations and stock results

Stock Acronym	Description	Equation	Expected Value [t]	Deviation [t]
LDFstock	Landfill	$\mathbf{waste_{LDF}}$ * $\mathbf{c_{LDF}}$	144,000	61,000
PHHstock	Private Households<br>Buildings (BUI_PHH)<br>Consumer Goods (CONS)	BUI+CONS<br>BUI_PHH= $stock_{bui}$ * Cap_{VIE} * $Proxy_{area}$<br>CONS=$cons_{cap}$ * Cap_{VIE}	131,000<br>94,000<br>37,000	35,000<br>32,000<br>13,000
RDAstock	River Danube	Not identified	-	-
TECstock	Transport / Energy / Communication Infrastructure<br>Catenary (CAT)<br>Electricity Grid (EG)<br>Telecommunication (TEL)	CAT+EG+TEL<br>CAT= $\mathbf{l_{train,\ tram}}$ * $\mathbf{A_{CAT}}$<br>EG= $\mathbf{grid_{lenth}}$ * $\mathbf{c_{grid}}$<br>TEL=**Unit** * $\mathbf{C_{unit}}$	103,000<br>1,000<br>89,000<br>13,000	11,000<br>50<br>10,100<br>4,100
USTstock	Underground Storage	Not identified	-	-
ECOstock	Urban Industry, Business, Services & Forestry /	BUI_ECO= $stock_{bui}$ * Cap_{VIE} * (1-$Proxy_{area}$)	40,000	14,000
VEHstock	Vehicles	VEH_{stock} * c_{veh_stock}	24,000	2,000
UPVstock	Urban Pedosphere	A_{UPV} * t_{soil} * d_{soil} * c_{soil}	4,000	1,000

Note: t = metric tons per year

Table S5: List of input parameter (non bold=single values, bold=matrices)

**Input Parameter**	**Description of data**	**Unit**	**Value**	**Uncertainty level**	**Reference**	**Compiled data**
Cap_{AUT}	Inhabitants Austria	#	8,347,341	1	(Statistik Austria 2012)	
Cap_{VIE}	Inhabitants Vienna	#	1,674,909	1	(Lebhart 2010)	
Dep	Deposition Rate	g Cu/ha/yr	148,7	2	(Spiegel 2003)	
A	Total City Area	ha	41,487	1	(Lebhart 2010)	
A_{UHY}	Urban Hydrosphere (Open Water Bodies)	ha	1,933	1	(Lebhart 2010)	
A_{TEC}	Traffic Surface	ha	5,981	1	(Lebhart 2010)	
A_{PHH}	Residential Area	ha	10,267	1	(Lebhart 2010)	
A_{ECO}	Public Facilities and production areas	ha	4,381	1	(Lebhart 2010)	
A_{UPV}	Green Space Area	ha	18,925	1	(Lebhart 2010)	
$Cons_{AUT}$	National Cu Consumption in 2006	t Cu/yr	∑=108,000	2	(Daxbeck et al. 2006)	Table S7

Input Parameter	Description of data	Unit	Value	Uncertainty level	Reference	Compiled data
$Proxy_{Market\ Share}$	Global Market Share of Cu use	%		-	(ICF 2001)	Table S8
$Proxy_{pre\text{-}products,\ scrap}$	Ratio of sector based pre-product consumption for Cu scrap	%		-		Table S8 Figure S4
$Proxy_{pre\text{-}products,\ Cable}$	Ratio of sector based pre-product consumption for cables	%		-		Table S8 Figure S5
$Proxy_{Employees,\ ind}$	Ratio of Employees in industries using metals such as Cu on Viennese and Austrian scale	-	0.12	1.5		Table S9
$Proxy_{Employees,\ bui}$	Ratio of Employees in building industry on Viennese and Austrian scale	-	0.17	1.5		Table S9
$EEA_{I/E}$	Difference of Viennese EEA imports and exports of EEA	t	43,600		(Magistratsabteilung 05 2011)	
EEA_{PHH}	EEA consumed by PHH in Vienna	t	32,813	1	(EAK 2009)	
c_{EEA}	Average Cu concentration in EEA	%	0.1-8.0	1.6-3.0	(Truttmann et al. 2005; Wittmer 2006; Hausmann 2005)	Table S11 12, 13
VEH	Number of new registered vehicles and new rolling stock in Vienna	#	80,445	1	(Statistik Austria 2008; Lebhart 2010)	Table S15
c_{veh}	Cu concentration of specific vehicle types	kg Cu/#	4-80	1.3-2.5	(Wittmer 2006; ICF 2000; European Copper Institute (ECI) 2011; Bertram et al. 2002; Struckl 2007; Graedel et al. 2002)	Table S14 Table S15
Food	Food imports to Vienna	t	1,236	1	(Magistratsabteilung 05 2011)	Table S16
C_{food}	Cu concentration in different food types	mg/100 g	0.002-0.900	2	(USDA 2011)	Table S16
Dem	Demolition waste (5 categories)	t	1,770,091			Table S21
c_{dem}	Cu concentrations in demolition waste (5 categories)	mg/kg	22-670		(Brunner and Stampfli 1993; Schnöller et al. 2010)	Table S21
$Proxy_{area}$	Area Ratio of residential land use in contrast to total built area	%	70	2	(Lebhart 2010)	Table S18
Scrab	Amount of Cu scrap	t	4,982		(Wiener Umweltschutzabteilung MA22 2011)	
c_{scrabl}	Cu concentration in cooper scrap	%	90-99.9			
Cable	Amount of cable waste	t	2,400		(Wiener Umweltschutzabteilung MA22 2011)	-
c_{Cable}	Cu content of cables from demolished buildings	%	37		(Skutan 2008)	-
Vehicles	Number of vehicles signed on in Vienna and new rolling stock	#	80,445			Table S15
$c_{vehicles}$	Vehicle specific Cu content	kg Cu/vehicle	5-69			Table S15
Car_{dereg}	Deregistered Cars in Austria	#	993,354			
$Car_{impreused}$	Imported second hand cars in Austria	#	28,696		(Wirtschaftskammer Österreich 2012)	
Car_{rereg}	Re-Registered Cars in Austria	#	738,690			
Car_{dereg_exp}	Deregistered and exported cars in Austria	#	37,629			
Car_{Shred}	Shredded cars in Austria	#	63,975		(BMLFUW 2011)	
r_{car}	Proxy representing Viennese and Austrian car ownership	%	0.1534	-		
c_{car}	Cu content in cars	kg Cu/car	24			
m_{hhw}	Mixed waste from households and similar institutions	t/yr	579,888			
c_{hhw}	Cu concentration in mixed waste	mg/kg	1,990			
m_{wib}	Five Waste fractions from industry and business	t/yr	15,216		(Wiener Umweltschutzabteilung MA22 2011)	Table S38
c_{wib}	Cu concentrations in specific waste flows	%	12-99,9		(Daxbeck et al. 2006)	Table S38
m_{BW}	Collected bulky waste flow	t/yr	69,422	1.5	(Wiener Umweltschutzabteilung MA22 2011)	

Input Parameter	Description of data	Unit	Value	Uncertainty level	Reference	Compiled data
c_{BW}	Average Cu concentration in bulky waste	mg/kg	2,437	2.07	(Skutan and Brunner 2006)	
$m_{WEE, PHH}$	Collected WEEE in PHH of Vienna	t/a	9,044	1	(EAK 2009)	Table S40
c_{WEE}	Cu concentrations of 4 different WEEE fractions	%	1-8	2-5,6	(Wittmer 2006)	Table S40
$Proxy_{WEE, ECO}$	Proxy for allocating WEEE to ECO	-	0.066		(EAK 2009)	Table S42
m_{uptake}	Cu uptake	kg Cu/ha/yr	0.01-0.045		(Berger et al. 2011)	Table S45
$Proxy_{Land\ use,\ agriculture}$	Agricultural area splitted into different land use categories	ha	∑=6,281		(Fitzthum 2009)	Table S45
p_{rate}	Pesticide rate	kg Cu/ha/yr	0-3.0	2	(Berger et al. 2011)	Table S46
A_{agr}	Agricultural area splitted into different land use categories and organic/traditional farming	ha	∑=6,281	1	(Fitzthum 2009)	Table S47
$m_{fertilizer}$	Fertilizer rate	kg Cu/ha/yr	0.03-0.55	2	(Fricke and Höhl 2000; Umweltbundesamt 2001; Zethner et al. 2007; Berger et al. 2011)	Table S47
mil	Mileage of low duty vehicles in Vienna	Veh-km	5,694,000,000	-	(Holzapfel and Riedel 2011)	
e_{LDV}	Total wear rate of brake lining in low duty vehicles	g /veh-km	12.5	-	(Winther and Slento 2010)	
c_{brake}	Cu content of brake linings in low duty vehicles	%	14.231	-	(Figi et al. 2010, own calculations to represent the Austrian vehicle fleet)	Table S26
$TC_{VEH, TEC}$	Transfer coefficients describing the ratio of particulate emissions entering individual sinks	%		-	(Müller et al. 2008)	Table S25
l_{train}	Track length of the train network, owned by National Railway Company ÖBB	track km	761	-	(ÖBB-Holding AG 2008)	
l_{tram}	Track length of the tram network, owned by Wiener Linien	track km	363	-	(Wiener Linien 2008)	
e_{ROL}	Cu abrasion rate of break and wheel wear in trams and trains	g/track km/yr	1,200	-	(Burkhardt et al. 2005)	
$l_{TRAM, TRAIN}$	Track length of train and tram network according to the catenary diameter	track km	∑=1,124	-	(ÖBB-Holding AG 2008; Wiener Linien 2008)	Table S28
A_{CAT}	Cross section area of individual catenaries	mm	65-120	-		Table S28
a_{CAT}	Average lifetime of a catenary	yr	40	-		
A_{loss}	Average cross section loss over the whole lifetime	%	20	-		
$Proxy_{exp\_good}$	Proxy for estimating physical exports based on reported flow data (headquarter approach)	%	50		Assumption	
Imp_{good}	Imported Cu for producing industries		4,619		see flow PRO EXAECO	
Exp_{good}	Cu waste from production		5,363		see flow WAS ECOCTS	
m_{WR}	Sum of seven Cu waste flows that contain potential recyclables (demolition material, Cu scrap, cables, waste from industry and business, bulky waste, WEEE, Recycled EOL vehicles)	t/yr	∑=11,071	-		Table S44
$m_{bottom\ ash}$	Amount of bottom ash and fly ash	t/yr	125,016	1	(Wiener Umweltschutzabteilung MA22 2011)	
$c_{bottom\ ash}$	Cu concentration in bottom ash	kg Cu/t	7,885	2	(Skutan and Rechberger 2007; Mitterbauer et al. 2009)	
$m_{APC\ res}$	Amount of APC residues	t/yr	18,189	1	(Wiener Umweltschutzabteilung MA22 2011)	
$c_{APC\ res}$	Cu concentration in APC residues	t Cu/t	0.0009011	1.009	(Skutan and Rechberger 2007; Taverna et al. 2011)	
$m_{cu,\ inflow}$	Cu Inflow to Waste Water Treatment Plant	t Cu/yr	16.2	1	(Kroiss et al. 2008)	
$TC_{cu,\ WWTP}$	Transfer Coefficients for Cu in Waste Water Treatment Plant	%	6-85	1	(Kroiss et al. 2008)	Table S32
$Area_{Roof}$	Total Roof Area	*m²*	52.000.000	3	(Kubu 2011)	
$Ratio_{Curoof}$	Percentage of Cu roofs	*%*	5	-	(Wocilka and Höfner	

Input Parameter	Description of data	Unit	Value	Uncertainty level	Reference	Compiled data
					2011)	
r_{roof}	Cu Runoff rate	*g/m²/yr*	1.365	0.654		Table S30
$Proxy_{PHH, roof}$	Allocation of roof runoff to PHH	*%*	70	-	(Lebhart 2010)	Table S31
$m_{tabwater}$	Revenue water	*m3/yr*	122.775.000	1	(Tomenendal 2011)	
$Loss_{water}$	Water Loss rate	*%*	10	-	(Daxbeck et al. 1996, adopted for 2008 data)	
$c_{cu, tabwater}$	Cu concentration in tap water at the network endpoints (geometric mean of five measurements in private households)	*µg/l*	20.675	3.26	(Magistratsabteilung 31 2008)	
c_{ww}	Average Cu disposal in waste water	*mg Cu/cap/yr*	843	1	See table	Table S33
c_{bf}	Cu in bodily fluids	*mg Cu/cap/yr*	760	1	See table	Table S34
m_{street}	Mass Flow of collected street litter	*t/yr*	39,495	1	(Wiener Umweltschutzabteilung MA22 2011)	
c_{street}	Cu concentration in street litter	*mg Cu/t*	46	2	(Boller 2002)	
m_{mw}	Six mixed waste fractions, thermally treated in Vienna	*t/yr*	∑=598,088	1	(Kronberger 2011)	Table S36
c_{mw}	Average Cu content in six mixed waste fractions	*mg Cu/t*	45-1990	0.97-2	(Skutan and Brunner 2006; Skutan and Rechberger 2007; Umweltbundesamt 2000; Boller 2002; Hausmann 2005; Arx 2006)	Table S37
m_{comp}	Applied compost on land	*t/yr*	33,000	1	(Weinmar 2011)	
w_{comp}	Water content of compost	*%*	41	2	(Umweltbundesamt 2000)	
c_{comp}	Legal limit of Cu concentration in compost, quality level A+	*mg Cu/kg*	70	2	(Republik Österreich 2001a)	
$Ratio_{SPW}$	Percentage of surface runoff ending up as combined sewer overflow	*%*	50	-	(Fenz 1999)	
$Ratio_{SST}$	Ratio surface water collected by mixed sewer system based on network lengths	*%*	81	-	(Lehmann 2011; I C Consultants Ltd 2001; Lampert et al. 1997)	
$stock_{bui}$	Cu stock in buildings	*kg Cu/cap*	80	2	(Wittmer 2006)	
$cons_{cap}$	Cu stock in consumer goods	*kg Cu/cap*	22	2	(Wittmer 2006)	
VEH_{stock}	Number of registered vehicles	*#*	∑=850,784	1	(Statistik Austria 2008; Wiener Linien 2009)	Table S50
c_{veh_stock}	Cu content of different vehicle types	*kg Cu/#*	4-1,500	0.6-12.47	(Bertram et al. 2002; European Copper Institute (ECI) 2011; Hoock 2008; Struckl 2007)	Table S50
$grid_{lenth}$	Cable lengths in the electricity grid	*km*	∑=22,477	1	(Wien Energie 2010)	Table S48
c_{grid}	Cu content of different cable types	*t Cu/km*	4-11	2	(Wittmer 2006)	Table S48
Unit	Number of different business units and private households	*#*	∑=859,397	1		Table S49
C_{unit}	Cu stock per business unit and private households	*kg Cu/#*	7-620	3		Table S49
t_{soil}	Soil depth	*m*	0.30	-	own assumption	
d_{soil}	Soil density	*t/m3*	1.7	-	own assumption	
c_{soil}	Average Cu concentration in Viennas top soil layer (land use category park and playgrounds)	*kg Cu/kg*	46		(Kreiner 2004, data analyis based on published data)	Table S53 Figure S10
$waste_{LDF}$	Mass of waste, disposed off in Viennas landfills and dumpsites	*t*	∑=81 Mio.	1	(Ableidinger et al. 2007)	Table S51 Table S52
c_{LDF}	Average Cu content in old dumped waste fractions	*mg/kg*	28-2600	2-4	(Brunner and Stampfli 1993; Woisetschlaeger et al. 2000)	Table S51 Table S52

Notes: # = quantity; t = metric ton; kg Cu/# = kilogram copper per quantity; ha = hectare; g Cu/ha/yr = gram copper per hectare per year; Veh-km = vehicle kilometer; track-km = track kilometer; g/m²/yr = gram per square meter per year; m² = square meter; µg/l = microgram per liter

2.2.2.3 Vienna - Background data

2.2.2.3.1 Products (PRO EXAECO)

2.2.2.3.1.1 Overview

Four groups of products are estimated to represent more than 90% of Cu in imported products:

1. Cu containing goods (Sheet metal, Wires, Cables, Pipes, Pre-Products etc.)
2. Electrical and electronic appliances
3. Vehicles
4. Food

Table S6: Imported products (PRO EXAECO)

Goods	Flux rate [t Cu/yr]	Deviation [t Cu/yr]	Allocation to ECO	Allocation to VEH	Allocation to PHH
Cu containing goods	4,619	625	4,619	-	
Electrical and Electronic Appliances	1,969	287	486	-	1,482
Vehicles	2,237	185	-	2,237	-
Food	2	0.25	-		2
Sum	8,827	341	5,105	2,237	1,484

Note: t Cu/yr = metric tons copper per year; ECO = Industry, business, services and forestry; VEH = Vehicles; PHH = Private Households

2.2.2.3.1.2 Cu containing goods

Table S7: Cu consumption in Austria (Daxbeck et al. 2006)

N°	Austrian Cu Consumption	Flux rate [t/yr]
1	Electrical conductors, cables	33,000
2	Pipes	26,000
3	Sheets	15,000
4	Wires, Cables, Ropes	12,000
5	Rest	22,000
	Sum of Cu consumed in Austria	108,000

Note: t/yr = metric tons per year

Table S8: Proxy: Cu Market share (ICF 2001)

Global Cu Consumption	Cables & Wires *[%]*	Pre-product Cu *[%]*	Pre-product Cu-Alloy *[%]*	Σ *[%]*
Building Sector [%]	20	12	8	40
Infrastructure [%]	13	1	1	15
Other Production Industries [%]	19	7	19	45
Total Product Share	52	20	28	100

Table S9: Proxy: Employees

	Austria	Vienna	Proxy	Reference
	[#]	*[#]*	*[]*	
Employed people in building industry	275,266	46,736	0.17	(Statistik Austria 2009c; Hauptverband der österreichischen Sozialversicherungsträger SV 2011)
Employed people in industries using metals such as Cu (ÖNACE 2008 C24-C30 and C33)	345,926	42,081	0.12	

Note: # = quantity

Table S10: Vienna Cu consumption by sector: Downscaling and allocation of Cu consumption

	Austria			Vienna					
	Cables & Wires [t Cu/yr] (1)+(4)	Pre-product [t Cu/yr] (2)+(3)+(5)	Sum [t Cu/yr]	Proxy: Employees	Cables and Wires [t/yr]	Pre-product [t Cu/yr]	Sum [t Cu/yr]	Allocation to flow	Note
Building Sector	17,308	37,800	55,108	0.17	2,942	6,426	9,368	CON EXAECO	Follow sec. 2.2.2.1.3
Infrastructure	11,250	3,150	14,400	0.17	1,913	536	2,449	CON EXAECO	Follow sec. 2.2.2.1.3
Other producing Industries	16,442	22,050	38,492	0.12	1,973	2,646	**4,619**	**PRO EXAECO**	-
SUM	45,000	63,000	108,000		6,828	9,608	16,436		

Note: t Cu/cap/yr = metric tons copper per capita per year;

2.2.2.3.1.3 Electrical and electronic appliances

Table S11: Cu in electrical and electronic appliances (EEA) and allocation to PHH and ECO

	Cu in electrical and electronic appliances (EEA)	Flux rate [t/yr]	Ratio [%]	Reference
$EEA_{I/E}$	Difference of import and export of EEA	43,586	100	(Magistratsabteilung 05 2011)
**$\sum EEA_{PHH}$**	EEA consumed by PHH in Vienna	**32,813**	**75.3**	(EAK 2009)
	Large EEA	14,508		
	Fridges & Freezers	4,465		
	Monitors	4,022		
	Small EEA	9,442		
	Lamps	376		
	EEA consumed by ECO	**10,774**	**24.7**	

Note: t/yr = metric tons per year;

Table S12: EEA consumed by Private Households in Vienna

EEA consumed by PHH in Vienna	Flux rate	Cu concentration (min)	Cu concentration (max)	Cu concentration (geometric mean)	Uncertainty Level	Flux rate	Deviation
	[t/yr]	[%]	[%]	[%]		*[t Cu/yr]*	*[t Cu/yr]*
Large EEA	14,508	3.90	8.00	5.586	2.249	810	181
Fridges & Freezers	4,465	1.00	4.00	2.000	3.008	89	20
Monitors	4,022	3.90	7.80	5.515	2.211	222	50
Small EEA	9,442	2.90	5.00	3.808	1.957	360	80
Lamps	376	0.10	0.15	0.122	1.657	0	0
Total ($\sum EEA_{PHH}$)	32,813					1,482	205
Total ($\sum EEA_{ECO}$)						486	134
Total						1,969	287

Note: t/yr = metric tons per year; t Cu/yr = metric tons copper per year;

Table S13: Cu content of EEA

Cu content of electrical and electronic appliances	Reference	Cu concentration	
		[kg/#]	*[%]*
EEA in general	(Truttmann et al. 2005)	-	3,4
	Hausmann, 2005	-	4,3
Fridges & Freezers	(Truttmann et al. 2005)	2	4,0
	Wittmer, 2006	-	1,0
	Oguchi, Murakami et al., 2011	-	3,4
Washing Machine	(Truttmann et al. 2005)	1,8	2,4
	Oguchi, Murakami et al., 2011	-	3,1
Dishwasher	(Truttmann et al. 2005)	1,3	2,5
	Wittmer, 2006	-	2,0
Microwave	Truttmann et al., 2003	0,9	3,9
TV Set	Truttmann et al., 2003	1,5	5,0
	Oguchi, Murakami et al., 2011	-	0,9 - 3,9
Monitor CRT	Truttmann et al., 2003	1,1	7,8
	Oguchi, Murakami et al., 2011	-	3,9
Computer excl. Monitor	Truttmann et al., 2003	0,5	4,8
	Wittmer, 2006	-	4,0
	Oguchi, Murakami et al., 2011	-	0,9 - 1,0
Video recorder	Truttmann et al., 2003	0,3	6,0
	Wittmer, 2006	-	4,0
	Oguchi, Murakami et al., 2011	-	2,9
Telephone	Oguchi, Murakami et al., 2011	-	10,3
Air conditioner	Oguchi, Murakami et al., 2011	-	17,8

Note: kg/# = kilogram per item

2.2.2.3.1.4 Vehicles (VEH ECOVEH)

Table S14: Cu contents of vehicles – literature overview

Vehicle type & Reference	Cu Content [Mass %]	Cu Content [kg/#]	Notes
Cars			
(Keoleian et al. 1997)	1,4	19	Cu and Brass in U.S.-Built Car 1994
(ICF 2000)	n.a	17	Wires and Cables in Cars
(Bertram et al. 2002)	1,4	14	PKW from 80's average weight: 1000 kg
(Graedel et al. 2002)	1,4	20	Car produced between 1990-1994 average weight 1395 kg
(Hoock 2008)	2	29	BMW 3 series
(European Copper Institute (ECI) 2011)	n.a.	25	
Busses/Trucks			
(Bertram et al. 2002)	0,5	60 - 80	0,5% assumed total Mass 12.000 kg or more
Locomotive			
(European Copper Institute (ECI) 2011)	n.a.	8000	
Airplane			
(European Copper Institute (ECI) 2011)	2	n.a.	
Motorbikes			
Assumption	2	n.a.	Same as in modern cars
Subway cars			
(Struckl 2007)	3	1.300 – 1.500	Average weight of subway car in Vienna: 42 t
Trams			
Calculation based on (Struckl 2007)	3	700 - 800	Average weight of tram car in Vienna 23 t

Note: kg/# = kilogram per item

Table S15: Import of vehicles and transportation items

Vehicles and transportation items	Number of new registered vehicles in Vienna and new rolling stock #	Uncertainty Level	Cu (min) *[kg Cu/#]*	Cu (max) *[kg Cu/#]*	Cu (geom.. mean) *[kg Cu/#]*	Uncertainty Level	Flux rate *[t Cu/a]*	Deviation
New registered vehicles								
Cars	63.473	1	20	29	24	1.570	1.529	176
Trucks	8.088	1	60	80	69	1.319	650	53
Motor Bikes	7.856	1	4	6	5	1.657	38	5
Others (mainly heavy machinery, busses etc.)	976	1	25	60	39	2.466	38	11
Change in stock of Wr. Linien 2007/2008 - public transport system								
Subway Cars (Triebwagen)	52	1	1273	1500	1382	0.783	72	5
**Total (VEH ECOVEH)**	80,445						**2,237**	**185**

Note: kg Cu/# = kilogram copper per item

2.2.2.3.1.5 Food

Table S16: Cu imports via food

	Food	c_{food}	Cu	Standard Deviation
	[t/yr]	*[mg/100g]*	*[kg Cu/yr]*	*[kg Cu/yr]*
Fische, Krebstiere, Weichtiere	5.74	0.062	4	
Fleisch, Innereien, genießbarer Schlachtanfall	21.07	0.060	13	
Früchte	33.47	0.057	19	
Gemüse, Wurzeln, Knollen	33.30	0.079	26	
Getränke, alkoholische Flüssigkeiten, Essig	133.25	0.007	9	
Getreide	220.31	0.490	1,080	
Kaffee, Tee, Mate, Gewürze	53.44	0.011	6	
Milch, Milcherzeugnisse, Vogeleier, Honig	42.23	0.013	5	
Müllereierzeugnisse, Malz, Stärke	6.64	0.270	18	
Ölsaaten, Samen, Stroh, Futter	106.12	0.900	955	
Tierische und pflanzliche Öle, Fette, Wachse	167.70	0.002	3	
Verschiedene eßbare Zubereitungen	50.56	0.206	104	
Zubereit.v. Fleisch, Fisch, Krebs u. Weichtieren	7.87	0.061	5	
Zubereit.v. Gemüse, Früchte, and. Pflanzenteilen	40.49	0.068	27	
Zubereitungen v. Getreide, Mehl, Stärke, Milch	34.40	0.490	169	
Zucker und Zuckerwaren	279.10	0.007	20	
**Total**	**1,236**		**2,463**	**252**

Note: t/yr = metric tons/year; kg Cu/yr = kilogram copper per year; mg/100g = milligram per 100 gram

2.2.2.3.2 Construction material (CON EXAECO, CON ECOPHH, CON ECOTEC)

Table S17: Cu in construction material

Acronym	Sector		Flux rate		Calculation routine
			Expected Value [t Cu/yr]	Deviation [t Cu/yr]	
	Building Sector		9,368	1,338	See Table S10
CON ECOPHH		Private Households	6,567	938	Allocation of construction material in the building sector to PHH: 9368 * 70%... = 6,567 t/yr
		Economy, Business	2,802	400	70% = $Proxy_{Area}$
CON ECO TEC	Infrastructure		2,449	376	See Table S10
CON EXAECO	SUM		11,817		

Note: t Cu/yr = metric tons per year

Table S18: Proxy area

N°	Land use category	Area [ha]	Allocation to process …	Reference
1	Residential Area	10,267	PHH	(Lebhart 2010)
2	Public facilities	1,888	ECO	(Lebhart 2010)
3	Industry and Business	2,492	ECO	(Lebhart 2010)
4	Construction Areas Total	14,647		
	Proxy = (1)/(4) *100	70%		

Notes: ha = hectare; PHH = Private Households; ECO = Industry, business, services and forestry

2.2.2.3.3 Construction waste (CWA ECOCTS, CWA PHHCTS, CWA TECCTS)

2.2.2.3.3.1 Overview

Table S19: Cu in construction materials

		Demolition material		Scrap metal		Cables		Sum	
Flow acronym	Waste generator	Expected Value [t Cu/yr]	Deviation [t Cu/yr]	Expected Value [t Cu/yr]	Deviation [t Cu/yr]	Expected Value [t Cu/yr]	Deviation [t Cu/yr]	Expected Value [t Cu/yr]	Deviation [t Cu/yr]
CWA PHHCTS	PHH	522	123	2,092	486	369	86	2,983	509
CWA ECOCTS	ECO	223	53	894	207	158	37	1,275	217
CWA TECCTS	TEC	90	14	249	62	343	86	682	107
	Total amount of Cu in construction material	835	135	3,235	532	870	127	4,940	563

Note: t Cu/yr = metric tons copper per year; PHH = Private Household; ECO = Industry, business, services and forestry; TEC = Transport, energy, and communication infrastructure

2.2.2.3.3.2 Demolition Material

Table S20: Cu in construction material

Cu content in construction materials	Cu content [mg/kg]	Reference
Total construction waste	670	(Brunner and Stampfli 1993)
Mineral fraction (Average)	22	(Schnöller et al. 2010)
Fine dust from construction waste	34	(Townsend et al. 2004)
Construction waste (literature research)	650 - 1950	(Arx 2006)
Construction timber	65	(Arx 2006)
Tar and Bitumen (Average)	80	(Schnöller et al. 2010)

Notes: mg/kg = milligram per kilogram

Table S21: Cu in construction waste

Construction waste in Vienna 2008	Attribution	Cu content [mg/kg]	Amount of Waste [t/yr]	Cu in Waste [t/yr]	$Proxy_{area}$	ECO	PHH	TEC
Construction waste	PHH&ECO	670	1.090.976	731	30% for ECO, 70% for PHH	219	512	
Concrete demolition	PHH&ECO	22-34	432.296	12	30% for ECO, 70% for PHH	4	8	
Road construction waste	TEC	670	121.182	80				80
Tar and Bitumen	TEC	80	83.543	10		-	-	10
Construction and demolition timber	PHH&ECO	65	42.094	3	30% for ECO, 70% for PHH	1	2	
Sum			1.770.091	~ 830		223	522	90

Notes: mg/kg = milligram per kilogram; t/yr = metric tons/year; PHH = Private Households; ECO = Industry, business, services and forestry; TEC = Transport, energy, and communication infrastructure

2.2.2.3.3.3 Scrap metal

Viennese wastes statistics report that 5,254 t Cu scrap with a Cu concentration of 90-99,9% was collected (Wiener Umweltschutzabteilung MA22 2011). That amounts 4,982 t Cu/yr. We allocated the flows to sectors based on the consumption ratio of “pre-products” first, and to the buildings in ECO and PHH with the land use area.

Figure S4: Allocation routine for Cu scrap (t/yr = metric tons per year; PHH = Private Households; ECO = Industry, business, services and forestry; TEC = Transport, energy, and communication infrastructure)

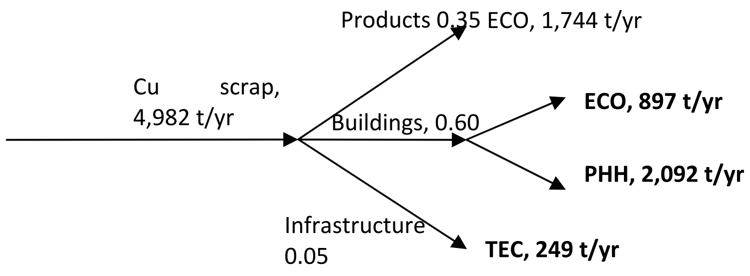

2.2.2.3.3.4 Cables

Figure S5: Allocation routine for cables (t/yr = metric tons per year; PHH = Private Households; ECO = Industry, business, services and forestry; TEC = Transport, energy, and communication infrastructure)

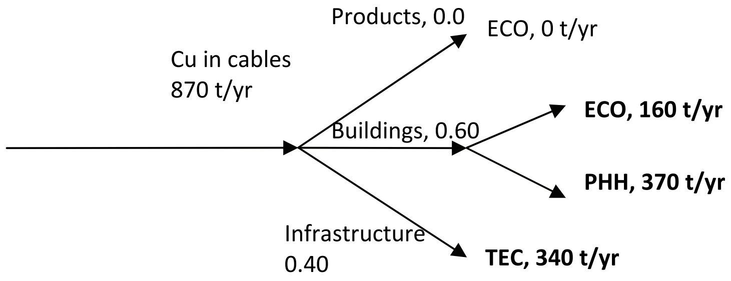

2.2.2.3.4 Vehicles

Figure S6: National car balances. Flows are given in number of cars per year, stocks are given in number of cars.

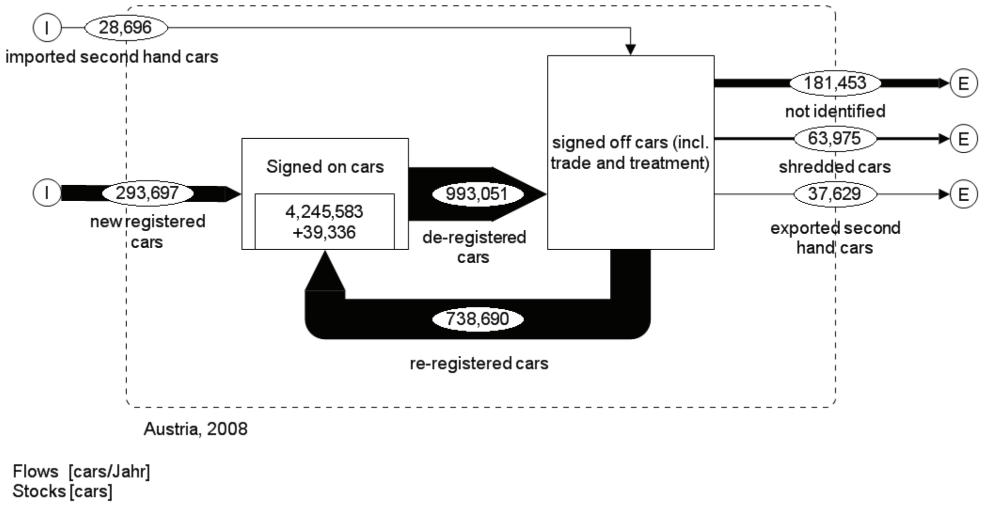

Table S22: National car balance

N°	Flow/stock name	#	Reference / Calculation routine
1	Imported second hand cars	28,696	(Wirtschaftskammer Österreich 2012)
2	New registered cars	293,697	(Statistik Austria 2009a)
3	De-registered cars	993,354	Calculated based on mass balance principle
4	Re-registered cars	738,690	(Statistik Austria 2009b)
5	Stock of signed on cars (31.12.2007)	4,245,583	(Statistik Austria 2008)
6	Stock of signed on cars (31.12.2008)	4,284,919	(Statistik Austria 2008)
7	Stock alteration of signed on cars	39,336	(6)-(5)
8	Shredded cars	63,975	(BMLFUW 2011)
9	Exported second hand cars	37,629	(Wirtschaftskammer Österreich 2012)
10	Not identified	181,756	Calculated based on mass balance principle

Note: # = number of items

Table S23: Downscaling of car numbers from the national to regional scale

Flow acronym	Flow name	Austria	Vienna	Cu [t/yr]	Uncertainty Factor	Deviation [t/yr]
WAS VEHCTS	Shredded cars	63,975	9,788	235	2	39
VEH VEHECO	Exported cars	37,629	5,757	138	3	68
VEH UID	Unidentified cars	181,756	27,809	667	3	330
TOTAL				1,040		339

Note: t/yr = metric tons per year

2.2.2.3.5 Particulate Emissions

2.2.2.3.6 Overview

Table S24: Compiled Results of particulate emissions and the whereabouts

sink / source	PBL	TEC	Deposition on vehicle	deposition on track	sum
	[t Cu/yr]	[t Cu/yr]	[t Cu/yr]	[t Cu/yr]	[t Cu/yr]
**Process VEH**					
Low duty vehicles	4.9±2.4	2±1	3.1	-	*10.0*
Rolling stock	0.1±0.3	0.2±0.6	0.2	0.9	*1.4*
Sum	*5±2.5*	*2.2±1.2*			
Flow Acronym	*PAE VEHPBL*	*PAE VEHTEC*	*3.3*	*0.9*	*11.4*
**Process TEC**					
Catenary Wiener Linien	0.2	0.2	1.4	-	*1.9*
Catenary ÖBB	0.4	1.8	0.3	0.6	*3.1*
Sum	*0.6±0.2*	*2.0±0.9*			
Flow Acronym	*PAE TECPBL*	*PAE TECTEC*	*1.7*	*0.6*	*5*

Note: t Cu/yr = metric tons copper per year; VEH = Vehicles; TEC = Transport, energy, and communication infrastructure

Table S25: Transfer coefficients

process	Low duty vehicles, brake wear	Train & trams, brake and wheel wear	Train catenary, ÖBB	Tram catenary, Wiener Linien
Ambient air	0.49	0.05	0.14	0.14
Deposition on surroundings (road debris, urban surfaces)	0.20	0.1	0.57	0.76
Deposition on vehicle	0.31	0.15	0.10	0.10
Deposition on track	-	0.7	0.19	0.00
Reference	(Oonk et al. 2005)	(Müller et al. 2008)	(Müller et al. 2008, modified)	(Müller et al. 2008, modified)

Notes: The transfer coefficients are adopted for urban areas

2.2.2.3.7 Brake wear

Table S26: Cu concentration in brake pads (low duty vehicles)

Market share of Car brands in Austria		Cu in brake pads (Figi et al. 2010)	Weighed average Cu content in brake pads of Austrian fleet
	[%]	[µg Cu / g break pad]	[µg Cu / g brake pad]
VW	20.4	138,935	28,343
Opel	8.2	169,080	13,865
Audi	6.6	106,080	7,001
Ford	6.2	151,420	9,388
Renault	5.6	139,980	7,839
Mercedes	5.4	190,240	10,273
BMW	4.9	93,140	4,564
Peugeot	4.5	135,375	6,092
Toyota	4.4	156,040	6,866
Mazda	4.3	142,254	6,117
Others	29.5	142,254	41,965
	100		142,312

Notes: [µg Cu / g brake pad = microgram copper per gram brake pad

Figure S7: Particulate emissions from vehicles and the whereabouts. Flows are given in metric tons per year.

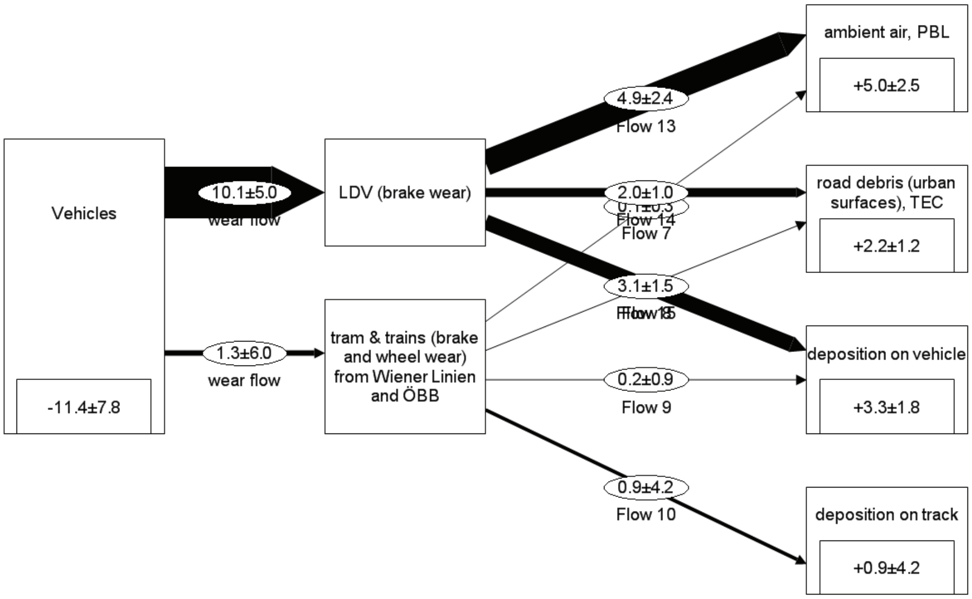

Table S27: Cu emissions from vehicles

Source	Flux Rate [t Cu/yr]	Uncertainty Level	Uncertainty Factor	Uncertainty
low duty vehicles	10.1	3	1.991	5.0
tram & trains	1.3	3	1.991	0.6

Note: t Cu/yr = tons copper per year

2.2.2.3.8 Catenary wear

The National Railway Company ÖBB operates 761 km electrified track with varying catenary diameters. The catenary material is made up from 99,5 – 99,9% Cu and 0,1-0,5% Ag or Cd. The catenary is replaced after 40 years; the remaining cross section is about 80%. The urban Public Transport Provider Wiener Linien operates 363 km electrified tram tracks (main tracks + station tracks). The cross section is assumed with 120mm2. Lifetime data are equally to ÖBB.

Table S28: Catenary wear in Vienna

Catenary stock & wear	Length	Cross section	Cu stock	Catenary wear
	[km]	[mm²]	[t]	[t/yr]
ÖBB, Section 1	272	65	158	0,1
ÖBB, Section 2	345	100	307	0,4
ÖBB, Section 3	144	120	154	0,8
ÖBB, Sum	761		619	3,1
Wiener Linien	363	120	389	1,9
Total	1.124		1.008	5,0

Note: km = kilometer; mm2 = square millimeter; t = metric tons; t/yr = metric tons per year

Figure S8: Catenary wear and the whereabouts. Flows are given in metric tons per year.

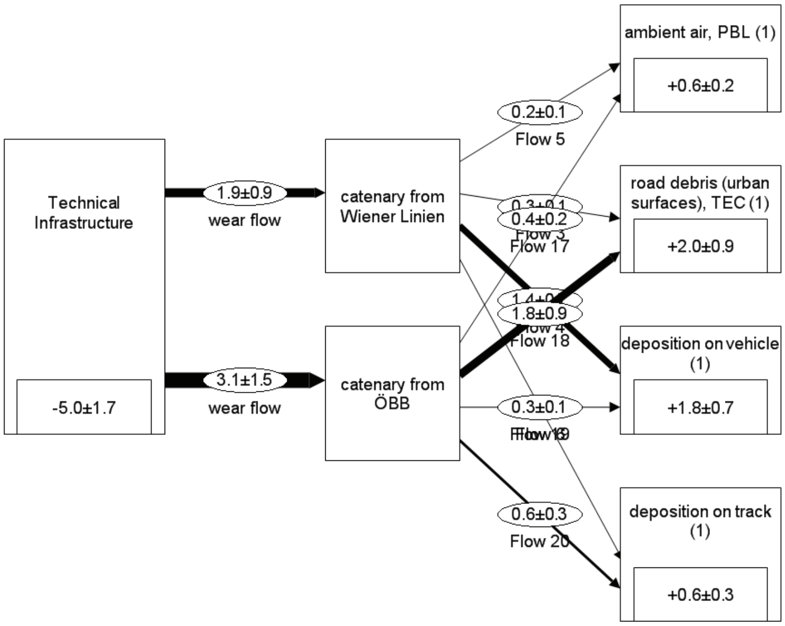

Table S29: Cu emissions from rolling stock

Source, catenary	Flux Rate [t Cu/yr]	Uncertainty Level	Uncertainty Factor	Uncertainty
Wiener Linien	1.9	3	1.991	0.9
ÖBB	3.1	3	1.991	1.5

Note: t Cu/yr = tons copper per year

2.2.2.3.9 Roof runoff

$$R = (0.37\mathrm{SO}_2^{0.5} + 0.96\ \mathrm{rain}10^{-0.62\ \mathrm{pH}})\left(\frac{\cos(\theta)}{\cos(45°)}\right)$$

Formula 1. Formula for the calculation of R, the Cu run-off rate from roofs (Odnevall Wallinder et al. 2007).

Table S30: Climate and air comparison Stockholm / Vienna

Corrosion rate of city surfaces	Year	pH rain [-]	Surface Inclination [°]	SO_2 [µg/m³]	O_3 [µg/m³]	Precipitation [mm]	Corrosion rate [g/m²/yr]
Stockholm	1995/1998	-	-	3,8 - 4,1	53 - 63	540	1,1 – 1,7
Vienna	2009	4,9-5,1	26	3	50	669	1.3-1.5

Notes: pH = pH value; µg/m³ = microgram per cubic meter; mm = millimeter; g/m²/yr = gram per square meter per year

Table S31: Land use as proxy for allocating roof runoff

Land use	Area [ha]	Proxy	Related to process
Residential Area (Wohngebiete)	10,267	0.70	PHH
Public Institution (Öffentliche Einrichtungen)	1,888	0.13	ECO
Commercial Area (Betriebsbaugebiete)	2,492	0.17	ECO
Total	14,647		

Notes: ha = hectare; PHH = Private Households; ECO = Industry, business, services and forestry;

2.2.2.3.10 Waste water flows

Table S32: Transfer Coefficients for Cu in WWTP

Material flow	Transfer Coefficients [%]	Flux Rate [t Cu/yr]
Grill Material	6.0	1.0
Sewage Sludge	84.2	13.6
Effluents	9.8	1.6
Total	100	16.2

Notes: t Cu/yr = metric tons copper per year

Table S33: Cu in waste water coming from anthropogenic activities in PHH

Source	Flux Rate [mg/cap/yr]	Reference
Cu from food preparation	70	(Baccini et al. 1993)
Cu from washing dishes	25	(Baccini et al. 1993)
Cu in toilet paper (from recycling paper)	64	(Baccini et al. 1993)
Cu from product use	30	(I C Consultants Ltd 2001)
Cu from washing clothes	218	(Baccini et al. 1993)
Cu from cleaning living areas	436	(Baccini et al. 1993)
Total	843	

Note: mg/cap/yr = milligram per capita per year

Table S34: Cu in waste water coming from humans

Source	Flux Rate [mg/cap/yr]	Reference
Cu in feces	708	(I C Consultants Ltd 2001)
Cu in urin	5	(Lampert et al. 1997)
Cu in skin particles	47	(Lampert et al. 1997)
Cu in human off-flows	760	

Notes: mg/cap/yr = milligram per capita per year

Figure S9: Cu in waterflows. Flows are given in metric tons copper per year.

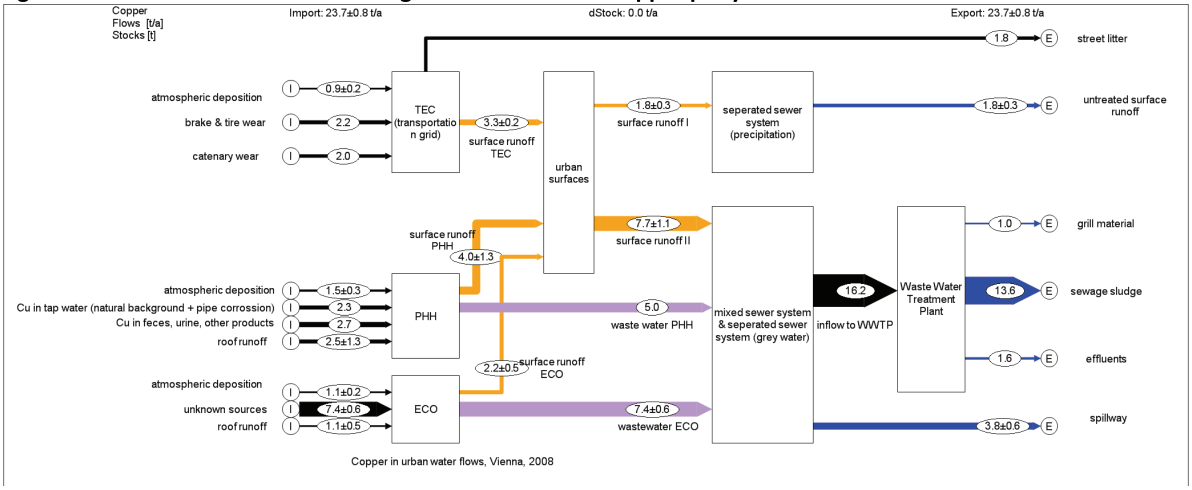

2.2.2.3.11 Solid Waste

2.2.2.3.11.1 Overview

Table S35: Compiled solid waste flow data

	PHH (HHW PHHCTS)		ECO (WAS ECOCTS)		total	
Composition of Solid Waste flow	Expected Value [t Cu/yr]	Deviation [t Cu/yr]	Expected Value [t Cu/yr]	Deviation [t Cu/yr]	Expected Value [t Cu/yr]	Deviation [t Cu/yr]
Municipal Solid Waste	1,123	79	-		1,123	79
Waste from industry and business	-	-	5,363	457	5,237	457
Bulky Waste	170	34	-		170	34
WEEE	400	52	27	3	427	52
Total	1,693	100	5,390	457	6,957	

Notes: t Cu/yr = metric tons copper per year; PHH = Private Households; ECO = Industry, business, services and forestry;

2.2.2.3.11.2 Municipal Solid Waste

Table S36: Cu flows in mixed waste

Cu waste input for incineration	Flux Rate [t]	Lower level [mg Cu/kg dm]	Upper level [mg Cu/kg dm]	Expected Value [mg/kg dm]	Cu Flux [t Cu/yr]	Deviation [t Cu/yr]
Municipal Solid Waste	525,982	1800	2200	1989.975	1,046.7	79.3
Organic waste collection	4,548			76	0.3	0.1
Plant waste collection	1,576			104.1	0.2	0.0
Bulky waste (Sperrmüll)	37,164	1800	2200	1989.975	74.0	5.6
Street litter	27,136			46	1.2	0.2
Paper & Plastic	1,682			65	0.1	0.0
total	598,088				1,122.5	79.5

Notes: t = metric tons; mg Cu/kg ds = milligram copper per kilogram dry matter; t Cu/yr = metric tons copper per year

Table S37: Cu concentrations is various mixed waste fractions

Cu content in waste	Cu content *[mg/kg]*	Reference
Municipal solid waste	2000	(Skutan and Rechberger 2007, p. 82; Skutan and Brunner 2006, p. 236)
Average Organic Waste Collection (Biotonne)	76	(Umweltbundesamt 2000)
Average Plant Waste Collection (Grünschnitt)	104	(Umweltbundesamt 2000)
Cu concentration in street litter	46	(Boller 2002)
Cu concentration in bulky waste	2.000	(Hausmann 2005)
Cu concentration in paper and plastic	65	(Arx 2006)

Note: mg/kg = milligram per kilogram

2.2.2.3.11.3 Waste from industry and business

Table S38: Waste from industry and business

key N°	Waste from industry and business	Amount of Waste in Vienna *[t/yr]*	Cu Content *[%]*	Cu in waste flow *Expected Value [t Cu/yr]*	*Deviation [t Cu/yr]*	Note
35301	Waste from punching, blanking (is recycled)	8,831	12.5%	1,104	191	
35315	Non-ferrous metal scrap (is recycled)	2,993	55%	1,646	285	
51104	Electroplating sludge	142	15-12%	37	14	
51530	Cu chloride	1,506	47-65%	832	86	
35310	Cu scrap from production industry	1,839	90-99,9%	1,744	290	See sec. 2.2.2.3.3
	Total			5,363	457	

Notes: t/yr = metric tons per year; t Cu/yr = metric tons copper per year; key N° = key number according to the Austrian waste management directive

2.2.2.3.11.4 Bulky waste

Table S39: Allocation of bulky waste flows

Flow	Collected bulky waste in Vienna, from PHH and ECO [t/yr]	Cu concentration Lower range [mg/kg]	Cu concentration Upper range [mg/kg]	Geometric mean [mg/kg]	Cu Flux [t Cu/yr]
Bulky Waste	69,422	1800.00	3300.00	2437.212	169

Notes: t/yr = metric tons per year; mg/kg = milligram per kilogram; t Cu/yr = metric tons copper per year;

2.2.2.3.12 WEEE

Table S40: WEEE from PHH

WEEE from PHH	Flow of goods, expected value [t]	Cu concentration Lower range [%]	Cu concentration Upper range [%]	Geometric mean [%]	Cu Flux rate [t Cu/yr]	Deviation [t Cu / yr]
Large WEEE	2,983	3.90	8.00	5.586	167	37
Fridges & Freezers	2,103	1.00	4.00	2.000	42	21
Monitors	2,387	3.90	7.80	5.515	132	28
Small WEEE	1,572	2.90	5.00	3.808	60	10
Sum Waste PHH - WEEE	9,044				400	52

Notes: t/yr = metric tons per year; t Cu/yr = metric tons copper per year;

Table S41: WEEE from ECO

WEEE from ECO	Flow of goods, expected value [t]	Cu concentration Lower range [%]	Cu concentration Upper range [%]	Geometric mean [%]	Cu Flux rate [t Cu/yr]	Deviation [t Cu / yr]
Small WEEE	198	3.90	8.00	5.586	11	2
Cu in WEEE Waste	139	1.00	4.00	2.000	3	1
Large WEEE	158	3.90	7.80	5.515	9	2
Fridges & Freezers	104	2.90	5.00	3.808	4	1
Sum Waste PHH - WEEE	599				27	3

Notes: t = metric tons; t Cu/yr = metric tons copper per year;

Table S42: Allocation of WEEE flows

Description	value	unit
Collected from private households	61,390,135	t/yr
Collected from economy	4,067,869	t/yr
Ratio = Proxy ECO/PHH	0,07	

Note: t/yr = metric tons per year; PHH = Private Households; ECO = Industry, business, services and forestry;

2.2.2.3.13 Exported products (PRO ANTEXA)

Table S43: Compiled data for exported flows

Flow acronym	Flow Name	Flux rate [t Cu/yr]	Deviation [t Cu/yr]	Calculation procedure and references
VEH VEHECO	Exported vehicles	138	68	See flow “Exported Vehicles”, sec. 2.2.2.3.4
-	Products from producing industry	950		After data are not available, we assumed that 50% of the difference from imported Cu for producing industries (see flow PRO EXAECO) and the waste from production (see flow WAS ECOCTS). 0.5*(4,600-2,700)=950
PRO ANTEXA		1,088		

Note: t Cu/yr = metric tons copper per year;

2.2.2.3.14 Exported waste including recyclables (REC CTSECO)

To estimate the recycling flow, we used the recycling potential of six generated waste flows, combined them with individual information regarding the whereabouts in recycling facilities.

Table S44: Cu waste including potential recyclables

Recyclables and waste	Potential recyclables in waste flow: Expected Value [t Cu/yr]	Deviation [t Cu/yr]	Calculation procedure and references
Demolition Material (construction waste, concrete demolition, Construction and demolition timber)	835<br>PHH (522)<br>ECO (223)<br>TEC (90)	135<br>(123)<br>(53)<br>(14)	See flow "Construction material", sec. 2.2.2.3.2. 835 t Cu/yr is estimated for demolition material. Even 75% (Ableidinger et al. 2007) of demolition material was sent out of the city, we assumed that 100% of Cu is sent out to recycling facilities out of Vienna.
Cu scrap	3,235<br>PHH (2,092)<br>ECO (894)<br>TEC (249)	532<br>(486)<br>(207)<br>(62)	See flow "Construction material", sec. 2.2.2.3.2. Cu scrap was reported in the Viennese waste statistics with an amount of 5,254 t/yr. An average Cu content of 90-99.5% results 4,982 t Cu/yr of which 3,235 are related to construction waste that goes to recycling facilities out of Vienna.
Cables	870<br>PHH (369<br>ECO (158)<br>TEC (343)	127<br>(86)<br>(37)<br>(86)	See flow "Construction material" sec. 2.2.2.3.2. The Viennese waste statistics accounted 2,400 t cables [42]; measurements reveal a Cu content of 37% in cables within demolition material [47]. Combining the two factors results 870 t Cu/a.
Subtotal, construction waste	4,940	563	
Waste from industry and business	5,363	457	See flow "solid waste", sec. 2.2.2.3.11
Bulky Waste	170	34	See flow "solid waste", sec. 2.2.2.3.11
WEEE	427	52	See flow "solid waste", sec. 2.2.2.3.11 WEEE is collected and partly dismantled in Vienna but separation to the metal fractions occurs in various specialized treatment plants outside the city (Spitzbart 2012). The total amount of Cu in WEEE collected in Vienna is ~430 t/yr.
Recycled End-of-Live Vehicles	235	39	See flow "EOL vehicles", sec. 2.2.2.3.4
Total	11,135	729	

Note: t Cu/yr = metric tons copper per year

2.2.2.3.15 Harvest

Plant uptake of Cu from soil range are estimated to be 43 g/(ha*a) in farmland to 51 g/(ha*a)in grassland (Zethner et al. 2007; Kühnen and Goldbach 2004) . If the plants are further harvested, this amount of Cu is actively removed from the soil. With an agricultural area of ~ 6.000 ha, the Cu flow in harvest is estimated to be ~ 0,3 t/yr. To test this values probability, detailed harvest information (Fitzthum 2009) combined with Cu take-up values for Crops, Wine and Fruit production which was published in an Austrian study (Berger et al. 2011). The Sum of Cu take-up in harvest is therefore estimated to be 0,3 t/yr (uncertainty factor: 1,34).

Table S45: Agricultural production

Cu in harvest products	Harvest 2008 [ha]	Cu take-up [kg Cu/ha/yr]	Cu [kg]
Agriculture & Horticulture	5,600	0,045	252
Wine production	600	0.01	6
Fruit production	70	0.03	2
Sum	6,270		~ 260

Notes: ha = hectare; kg Cu/ha/yr = kilogram copper per hectare per year; kg = kilogram;

2.2.2.3.16 Pesticides

Table S46: Pesticide inputs on soil

Pesticide Input	Area [ha]	Pesticide rates [kg Cu/ha/yr]	Cu Flux rate [t/yr]
Viticulture organic	109	3.000	0.327
Fruit Production organic	12	3.000	0.036
Agriculture & Horticulture organic	1,009	1.400	1.413
Viticulture	497	1.000	0.497
Fruit Production	55	1.500	0.082
Agriculture & Horticulture	4,599	0.000	0.000
Total	6,281		2.356

Notes: ha = hectare; kg Cu/ha/yr = kilogram copper per hectare per year; t = metric tons;

2.2.2.3.17 Fertilizer

Table S47: Fertilizer inputs on soil

Fertilizer Input	Area [ha]	Fertilizer rate [kg Cu/(ha/a)]	Cu flux rate [t/yr]
Viticulture organic	109	0.200	0.022
Fruit Production organic	12	0.200	0.002
Agriculture & Horticulture organic	1,009	0.550	0.555
Viticulture	497	0.030	0.015
Fruit Production	55	0.030	0.002
Agriculture & Horticulture	4,599	0.350	1.609
Total	6,281		2.205

Notes: ha = hectare; kg Cu/ha/yr = kilogram copper per hectare per year; t/yr = metric tons per year;

2.2.2.3.18 Cu stock in technical infrastructure (TECstock)

Table S48: Cu stocks in electricity grid

Electricity grid	Length of network [km]	Cu content in [t/km]		Amount of Cu [t]	
		min	max	min	max
Highest voltage	54	10.72		583	583
High voltage	375	10.72		4,015	4,015
Medium voltage	6,844	5.09		34,837	34,837
Low voltage	11,255	4.07		45,809	45,809
Overhead power lines	3,747	0.75	1.34	2,807	5,012
Sum	22,276			88,052	90,257
				89,154	

Notes: km = kilometer; t/km = metric tons per kilometer; min = minimum value; max = maximum value

Table S49: Cu stocks in telecommunication grid

Unit	Number of Units [#]	Cu in telecommunication [kg/#]	Cu stock [t]
Vienna businesses <5 employees	64,631	12	776
Vienna businesses >100 employees	1,142	620	708
Vienna businesses 5-99 employees	21,918	294	6,444
Households in Vienna	77,1706	7	5,402
Total			13,329

Notes: # = number of items; kg/# = kilogram per item; t = metric tons

2.2.2.3.19 Cu stock in Vehicles (VEHstock)

The total stock of Cu in transportation utilities (e.g. cars, busses, trucks, subway, trams etc.) is calculated to be ~24.000 t Cu (uncertainty: 17%) or ~ 15 kg/cap. This value fits well with the above described literature. The calculation is based on data on the number of registered vehicles with the city of Vienna and data on public transportation vehicles such as the number of subways and trams (Wiener Linien 2009; Statistik Austria 2008) uncertainty level 1,0 and corresponding Cu contents from literature (Struckl 2007; Bertram et al. 2002; Hoock 2008; European Copper Institute (ECI) 2011). Used literature values can be found at flow calculations VEH ECOVEH. Uncertainty factors of Cu content were calculated by using the range found in literature.

Table S50: Cu stock in vehicles

Item	Number of vehicles [#]	Cu content Min [kg/#]	Max [kg/#]	Geomittel [kg/#]	Cu Flux Rate Expected Value [t Cu/yr]	Deviation [t Cu/yr]
Cars	657192	20.00	29.00	24.0831892	15,827	1,616
Trucks	61488	60.00	80.00	69.2820323	4,260	330
Motor Bikes	73938	4.00	6.00	4.89897949	362	41
Others (mainly heavy machinery, busses incl. Wr. Linien etc.)	56857	25.00	60.00	38.7298335	2,202	605
Trams	832	700.00	800.00	748.331477	623	21
Subway Cars (Triebwagen)	477	1272.78	1500.00	1381.72655	659	28
					23,933	1,757

Notes: # = number of items, kg/# = kilogram per number of item; t Cu/yr = metric tons copper per year

2.2.2.3.20 Cu stock in Landfills (LDFstock)

In 2005, there were six operated landfills located in Vienna (Ableidinger et al. 2007). Another 36 (excluding remediation sites) old unofficial dumps and landfills, filled with household or construction waste can be found in official datasets (Umweltbundesamt Österreich 2012). The total amount of Cu stocks in Viennese landfills is estimated to be ~ 144.000 t of Cu (uncertainty factor 1,84). This value fits well with the first estimation of 100.000 t Cu in landfills (without unofficial waste dumps) quite well.

Table S51: Cu stock in landfills

		Amount of Cu		Uncertainty factor
		[t] Min	*[t] Max*	
Excavated Soil (1)+(2)+(3)+(4)	6,845,900	200	400	1,49
Construction Waste (5)	11,300,000	900	8.000	2,90
Treated municipal solid waste	-	27.000	71.000	1,62
Slag concrete as construction material (6)+(7)+(8)	27,240,000	30.000	35.000	3,24
Waste dumps (unofficial)	35,690,400	24.000	93.000	2,02
Total sum of Cu stock in landfills		82.000	210.000	1,84
Mean Value			144.000	1,84

Notes: t = metric tons

Table S52: Landfills in Vienna

Landfill	Landfill Type	Approved volume [m3]	Available capacity [m3]	Deposited waste volume [m3]	Density*) [t/m3]	Mass [t]	N°
Deponie Max	Excavated soil	97.000	0	97.000	1,7	164.900	(1)
Nassbaggerung Transportbeton	Excavated soil	2.900.000	300.000	2.600.000	1,7	4.420.000	(2)
Nassbaggerung Readymix	Excavated soil	1.600.000	350.000	1.250.000	1,7	2.125.000	(3)
Deponie Rendezvousberg (Kleedorfer)	Excavated soil	250.000	170.000	80.000	1,7	136.000	(4)
Deponie Langes Feld (3 Kompartimente)	Construction w.	7.400.000	1.750.000	5.650.000	2	11.300.000	(5)
	Mass waste	2.000.000	1.650.000	350.000	2,4	840.000	(6)
	Municipal solid w.	1.600.000	1.500.000	100.000	2,8	280.000	(7)
Deponie Rautenweg	Mass waste	14.000.000	3.000.000	11.000.000	2,4	26.400.000	(8)
total		29.847.000	8.720.000	21.127.000		45.665.900	

Note: * = own estimation; m3 = cubic meter; t/m3 = metric tons per cubic meter; t = metric tons

Deposited wastes include excavated soil (usually from construction sites), construction waste and treated municipal waste according to Austrian landfill regulations and furthermore some municipal solid waste is stored temporarily. Using Cu concentrations from earlier calculations for excavated soil (28–61 mg/kg), construction waste (80-670 mg/kg) and municipal solid waste (1000-2600 mg/kg) the total Cu stock was calculated. Especially for the landfills "Langes Feld" and "Rautenweg" calculations are difficult. Due to their age, it is difficult to determine a Cu content of the former input. For example, "Langes Feld" was originally built to receive construction and demolition waste from World War II. Additionally to the type of landfill, slags which are used as building material in Viennese landfills and their input since 1964 (when the first MSW incineration plan in Vienna was established) were estimated. Here, the input of 2008, calculated with ~ 1.100 t/yr as well as capacities of incineration plants since 1964 were used as a base value.

Additionally to official landfills, waste dumps or suspected waste dumps with a total Volume of ~ 30 Mio m^3 were included into calculations (Umweltbundesamt Österreich 2012). With an average density of 1,0–1,2 t/m^3 (Fellner 2012) and contents (partly suspected, partly tested) as a mixture of untreated municipal solid waste and construction waste (with unknown percentages), Cu stocks are estimated to be between 24.000 and 90.000 t.

2.2.2.3.21 Cu stock in urban soil (UPVstock)

Figure S10: Substance concentration in parks with playgrounds, given in milligram per kilogram (mg/kg). The box is defined by the 10% and 90% percentile and the median. The red and blue bars indicate orientation values.

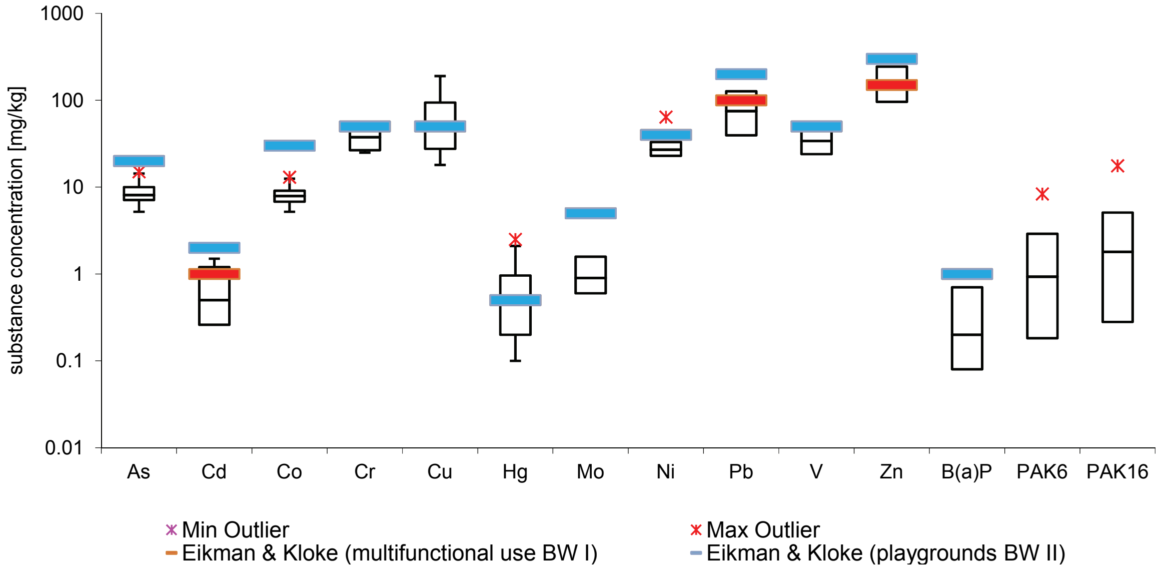

Table S53: Median concentrations in parks with playgrounds given in milligram per kilogram (mg/kg)

	As	Cd	Co	Cr	Cu	Hg	Mo	Ni	Pb	V	Zn	B(a)P	PAK6	PAK16
median	8,1	0,5	7,9	37,5	46,0	0,5	0,9	27,0	75,0	34,0	158,0	0,2	0,9	1,8
Eikman & Kloke playgrounds, BW2	20	2	30	50	50	0,5	5	40	200	50	300	1	-	-

2.2.2.4 Taipei – Background data

2.2.2.4.1 Atmospheric Deposition (ADE PBLUHY, ADE PBLUPV, ADE PBLECO, ADE PBLPHH, ADE PBLTEC)

In Huang (2004), metals in atmosphere within Taipei City were sampled and analyzed in the form of particulate matters and precipitation. Both wet and dry depositions of Cu were measured, as the rate of 3.4 $mg/m^2/yr$ and 3.8 $mg/m^2/yr$ respectively. Total Cu dry deposition rate was estimated by multiplying total area of Taipei, which is 0.92 t/yr. Also, atmospheric deposition of Cu through precipitation was calculated as 1.03 t/yr.

2.2.2.4.2 Products (PRO EXAECO, PRO ECOPHH)

Table S44: Ratio of Taipei and Taiwan for Imported Goods Estimation

Cu Application Category	Consumer Goods Total Retail Sales (NTD)	Industrial Equipment Areas of Factories (m^2)
Taipei	1,282,965,935,000	24,052,521
Taiwan	3,081,553,000,000	164,959,000
Ratio	0.42	0.15
Ratio in Taiwan	18%	21%
Ratio in Taipei	38.8%	15.9%

Notes: NTD = New Taiwan Dollar; m2 = square meter

Figure S15: Mechanism of Recycling Fund in Taiwan (Recycling Fund Management Board Taiwan 2010b)

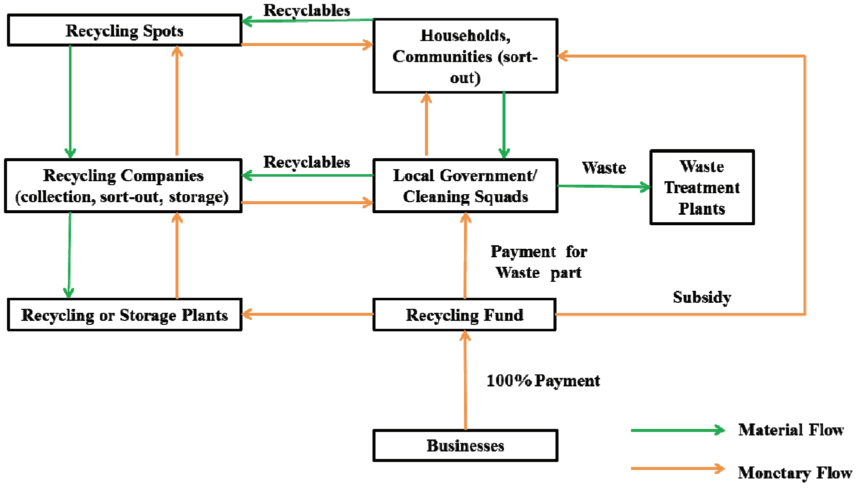

Table S45: Estimation of Household Appliances Number Consumed in Taiwan (2009)

WEEE	Recycling fund (2010): $ 928,883,523 NTD					
Item	Recycling Fee (NTD/#) (EPA 2004)	Amount of Audited Recyclables (unit) (Recycling Fund Management Board Taiwan 2010a)	Estimated Recycling income (NTD)	Ratio in WEEE Recycling Fund	Fund Distribution(NTD)	Estimated Amount of Products (#)
TV		504,978	123,467,121	28 %	257,518,274	1,053,244
CRT TV						
Over 25”	371					
Under 25”	247					
LCD, PDP TV						
Over 25”	233					

Under 25”	127					
Air Conditioner	248	326,283	80,918,184	18%	168,772,957	680,536
Washing Machine	317	292,000	92,564,000	21%	193,062,908	609,031
Refrigerator		293,869	148,403,845	33%	309,529,384	612,929
Over 250 L	606					
Under 250 L	404					

Note:WEEE = waste electrical and electronic equipment; NTD = New Taiwan Dollar; # = number of items; NTD/# = New Taiwan Dollar per item

Table S46: Estimation of Cu Mass in Household Appliances in Taiwan

Item	Conversion Rate (kg/#)	Conversion Weight (t)	Cu Content (Truttmann et al. 2005) (%)
TV	25.0	26,331.11	
CRT TV			3.9
LCD, PDP TV			0.8~1.2
Air Conditioner	60.0	40,832.17	17.8
Washing Machine	40.0	24,361.25	3.1
Refrigerator	50.0	30,646.47	3.4
Sum			

Note: kg/# = kilogram per item; # = number of items; t = metric tons;

Table S47: Estimation of Computer Appliances Number Consumed in Taiwan (2009)

WCA	Recycling fund (2010): $ 775,608,054 NTD				
	Notebook	Sales volume in Taiwan (2008): 1,042,772 units			
		Recycling Fee (NTD/unit): 39			
		Estimated recycling income: $ 40,668,108 NTD			
	Recycling Fund without Notebook (NTD): 734,939,946				
Item	Recycling Fee (NTD/#) (EPA 2004)	Amount of Audited Recyclables (unit) (Recycling Fund Management Board Taiwan 2010a)	Ratio in WCA Recycling Amount w/o Notebook	Estimated Recycling income (NTD)	Estimated Amount of Products (#)
Monitor		822,353	37%	269,264,506	1,658,713
CRT Monitor	127				
LCD Monitor					
over 25”	233				
under 25”	127				
Desktop PC	8.2, 49.2	839,120	37%	274,754,554	9,573,329
Printer		583,086	26%	190,920,886	1,552,202
Inkjet Printer	81				
Laser Printer	137				
Dot-matrix Printer	151				
Sum		2,244,559			

Notes: WCA = waste computer appliances; kg/# = kilogram per item; # = number of items; t = metric tons;

Table S48: Estimation of Cu in Computer Appliances in Taiwan

Item	Conversion (kg/#)	Rate	Conversion Weight (t)	Cu Content (%)
Notebook	4.0		4,171.09	1.00% (Oguchi et al. 2011)
Monitor	12.0		19,904.56	7.83% (Truttmann et al. 2005)
Desktop PC	12.0		114,879.95	0.90%[a]
Printer	8.5		13,193.72	3.20%[a]
Sum				

Notes: kg/# = kilogram per item; t = metric tons;

2.2.2.4.3 Construction Material (CON EXAECO, CON ECOTEC, CONECOPHH, CWA ECOCTS, CWA TECCTS, CWA PHHCTS)

Table S49: Estimation of Cu in Construction Waste

Taipei	Value	Unit
Total demolished floor area	25,000	m^2
Waste from C&D waste (Huang 1998)	1~1.28	t
Cu content in C&D waste (Huang 1998)	670~2,587	mg/kg

Notes: m2 = square meter; t = metric tons; mg/kg = milligram per kilogram

Table S50: Results of Cu in Construction Waste from PHH, CTS, and ECO

Land Use in Taipei	Area (ha)	Proportion
Urbanized Area	12,993.11	100%
**PHH**		41%
Residential District	3,793.06	
Commercial District	866.36	
Administration District	80.97	
Cultural District	73	
Special District	190	
Others	260.45	
**ECO**		3%
Industrial District	419.49	
**TEC**		56%
Public Facilities	7,309.78	

Notes: ha = hectare

2.2.2.4.4 Vehicles (VEH ECOVEH, WAS VEHCTS, VEH UID, VEH VEHECO)

Table S51: Estimation of Sales Volume of Vehicles in Taipei 2009

	Newly Registered Number	Sales Volume	Average weighted weights per unit (kg) (Liu 2009)	Mass of Sales Volume (t/yr)
Taiwan				
Cars	6,769,845	294,423		
Scooters/Motorcycles	14,604,330	478,268		
Taipei				
Cars	721,326	31,371	1,034	32,437,332.57
Scooters/Motorcycles	1,092,788	35,787	90.85	3,251,251.27
Sum				35,688,583.84

Notes: kg = kilogram; t/yr = metric tons per year

Table S52: Estimation of Recycled Vehicles in Taipei

	Newly Registered Number [#]	Average weighted weights per unit (kg) (Liu 2009)	Recycled Mass (t)
Taiwan			
Cars	6,769,845	1,034	130,791
Scooters/Motorcycles	14,604,330	90.85	21,994
Taipei			
Cars	721,326	1,034	195
Scooters/Motorcycles	1,092,788	90.85	23
Sum			

Notes: t = metric tons; # = number of items

Table S53: Estimation of Exported Vehicles

	Newly Registered Number [#]	Change of Registration (2009-2008)
Taiwan		
Cars	6,769,845	42,929
Scooters/Motorcycles	14,604,330	238,888
Taipei		
Cars	721,326	4,574
Scooters/Motorcycles	1,092,788	17,875
Sum		

Note: # = Number of items

2.2.2.4.5 Solid waste (HWW PHHCTS, WAS ECOCTS)

Table S54: Result of Cu Mass in Household Waste

Source of Waste	Cu Content	Amount (t/yr)
By Cleaning Squads	184.92~577.13 mg/kg	368,253.67
By People Themselves		13,974.27
Recyclables	(%)	
TV	0.8~3.9	5,302.27
Air Conditioner	17.8	8,222.33
Washing Machine	3.1	4,906
Refrigerator	3.4	6,171.25
Laptop	1	75.29
Monitor	7.83	4,144.66
Motherboard	0.9	4,229.16
Printer	3.2	2,081.62
Sum		

Note: t/yr = metric tons per year

2.2.2.4.6 Incineration: Mixed waste, exported residues for underground storage, residues Disposable waste (WAS CTSINC, TTR INCEXA, RES INCLDF)

Table S55: Residues of Incinerators

	Amount (t)	Cu Content (mg/kg) (Chen 2008)
Fly Ash	20,000	1028~1220
Bottom Ash	110,000	889~3136

Note: t = metric ton; mg/kg = milligram per kilogram

2.2.2.4.7 Composting: Compostable waste, residues, compost as fertilizer (WAS CTSCOM, RES COMCTS, COM COMUPV)

Table S56: Estimation of Compost Generated in Taipei City

Data source	Uncooked food waste (t/yr)	Compost (t/yr)	Cu content (mg/kg)
Muzha Incinerator	16,625.3	221.15	0.69
Waste in Taipei City	59,371.23 (Department of Environmental Protection of Taipei City Government 2009)	789.76	0.69

Note: t/yr = metric tons per year; mg/kg = milligram per kilogram

2.2.2.4.8 Stocks in ECO, PHH

Table S57: Estimation of the In-Use Household Appliances

Item	Average Number Per Hundred Households in Taipei (#)	Estimated Amount of In-use Stock (#)	Conversion Rate (kg/#)	Conversion Weight (t)	Cu Content (%) (Oguchi et al. 2011)
TV	155.87	1,500,764.68	25.0	37,519.12	0.8~3.9
Air Conditioner	224.39	2,160,496.48	60.0	129,629.79	17.8
Washing Machine	99.26	955,706.05	40.0	38,228.24	3.1
Laptop	123.03 (IDC Taiwan 2008)	1,184,570.98	4.0	4,738.28	1.0
Desktop	123.03	1,184,570.98	12.0	14,214.85	0.9

Note: # = number of items; kg/# = kilogram per item; t = metric ton;

Table S58: Information of the Tainan Case (construction completed in 1997)

Structure	Floors		Usage
Aboveground	13	1F	Shop
		2F~13F	Congregate Housing
Underground	3	B1~B3	Parking Lot

Table S59: Estimation of Wire and Cable Usage in Buildings

The Tainan Case	Amount	Unit
Wire and Cable Used	17,763.84	kg
Total Floor Area	1.79	kg/m^2
Total Floor Area in Taipei	171,185,000	m^2
Estimated Wire and Cable Used	306,372,155	kg
Cu Content	40~60 (Ruhrberg 2006; Spatari et al. 2002)	%

Note: kg = kilogram; kg/m2 = kilogram per square meter;

2.2.2.4.9 Stocks in VEH

Table S60: Estimation of Cu Stock in Transportation

Vehicles	Number of Registration/In Operation (Environmental Protection Administration - Executive Yuan 2010)	Average Weighted Weight Per Unit (kg) (Liu 2009)	Cu Content
Cars	721,326	1,034	1.4%
Scooters	1,092,788	90.85	1.4 %
Buses	2,628	--	60~80 kg/#

2.3 Stock and flow results

Table S54 shows the model inputs for each city in column (3), (4), (7), (8) and the balanced results in column (5), (6), (9), (10).

Table S54: Flow list.

		TAIPEI				VIENNA			
Flow	Flow name	Mass flow [t/yr]	± Mass flow [t/yr]	Mass flow (calculated) [t/yr]	± Mass flow (calculated) [t/yr]	Mass flow [t/yr]	± Mass flow [t/yr]	Mass flow (calculated) [t/yr]	± Mass flow (calculated) [t/yr]
(1)	(2)	(3)	(4)	(5)	(6)	(7)	(8)	(9)	(10)
ADE PBLECO	Atmospheric Deposition ECO	0.02	0.00	0.02	0.00	1.10	0.19	1.09	0.19
ADE PBLPHH	Atmospheric Deposition PHH	0.19	0.01	0.19	0.01	1.50	0.26	1.49	0.26
ADE PBLTEC	Atmospheric Deposition TEC	0.28	0.02	0.28	0.02	0.90	0.15	0.90	0.15
ADE PBLUHY	Atmospheric Deposition UHY	0.07	0.01	0.07	0.00	0.30	0.10	0.30	0.10
ADE PBLUPV	Atmospheric deposition PV	0.29	0.02	0.29	0.02	2.80	0.50	2.76	0.49
COM ANTUPV	Compost	0.04	0.01	0.04	0.00	1.40	0.30	1.40	0.21
CON ECOPHH	Construction Material	8,164.59	1,420.10	8,164.59	1,420.10	6,567.00	838.00	6,567.00	838.00
CON ECOTEC	Construction material	4,224.21	734.73	4224.21	734.73	2,448.00	376.00	2,448.00	376.00
CON EXAECO	Imported construction material	13,073.32	2,273.89	13,073.32	2,273.89	11,817.00	1,390.00	11,817.00	1,390.00
CWA ECOCTS	Construction waste ECO	2.79	1.36	2.82	1.36	1,275.00	217.00	1,266.04	212.19
CWA PHHCTS	Construction waste PHH	150.86	73.44	223.07	69.99	2,983.00	509.00	2,933.70	443.42
CWA TECCTS	Construction waste TEC	48.66	23.64	56.15	23.53	862.00	107.00	859.82	106.43
EFF WWSHYD	Effluents	2.85	0.22	2.73	0.22	1.60	0.10	1.60	0.10
FER ECOUPV	Fertilizer	0.00		0.00		2.20	0.30	2.20	0.30
HAR UPVECO	Harvest	0.00		0.00		0.30	0.10	0.30	0.10
HHW PHHCTS	Household waste	1,672.34	54.77	1,712.52	53.36	1,693.00	100.00	1,691.10	99.53
PAE TECPBL	Particulate emissions	0.00		0.00		0.60	0.20	0.61	0.20
PAE VEHPBL	Particulate emissions	6.10	2.67	0.84	0.03	5.00	2.50	5.94	0.63
PAE VEHTEC	Particulate emissions	0.00		0.00		2.20	1.20	2.20	1.20
PES UANUPV	Pesticides	0.00		0.00		2.40	0.30	2.40	0.30
PRO ECOEXA	Exported Products	7,944.63	1,359.73	7,944.63	1,359.73	2,574.00	746.00	2,574.00	746.00
PRO ECOPHH	Consumer goods & food	5,286.44	1,610.12	5,286.44	1,610.12	1,484.00	205.00	1,484.00	205.00
PRO EXAECO	Imported products	15,773.73	2,743.58	15,773.73	2,743.58	8,827.00	728.00	8,827.00	728.00
RES INCLDF	Residues	209.08	81.53	164.72	55.91	986.00	170.00	1,097.19	72.22
SPW WWSHYD	Combined sewer overflow	0.00		0.00		3.80	0.80	3.86	0.78
SRO ECOWWS	Surface runoff	0.02	0.01	0.02	0.01	2.20	0.50	2.18	0.49
SRO PHHWWS	Surface runoff PHH	0.51	0.25	0.66	0.25	4.00	1.30	3.84	1.20
SRO TECWWS	Surface runoff	0.35	0.18	0.43	0.18	3.30	1.50	3.09	1.35
SRO WMIHYD	Surface Runoff	0.90	0.40	0.51	0.40	1.80	0.05	1.80	0.05
SSL WWSCTS	sewage sludge	58.28	4.51	9.08	0.92	13.60	1.10	13.71	1.04
TTR INCEXA	Exported residues for underground	0.00		0.00		16.40	1.30	16.41	1.30
VEH ECOVEH	vehicles	499.64	250.02	499.64	250.02	2,237.00	185.00	2,237.00	185.00
VEH UID	Unidentified cars	0.00		0.00		498.00	330.00	498.00	330.00
VEH VEHECO	EOL vehilces for export	88.95	44.42	88.95	44.42	138.00	68.00	138.00	68.00

WAS CTSCOM	Compostable waste	0.04	0.01	0.04	0.00	1.40	0.30	1.40	0.21
WAS CTSEXA	Exported waste incl. recyclables	2,738.32	184.80	2,281.03	119.63	11,135.00	729.00	11,236.14	518.30
WAS CTSINC	waste	209.08	81.26	164.72	55.91	1,137.00	80.00	1,113.59	72.21
WAS ECOCTS	Solid waste ECO	58.42	22.64	65.28	22.54	5,390.00	457.00	5,350.26	410.20
WAS TECCTS	street litter	0.00		0.00		1.80	0.05	1.80	0.05
WAS VEHCTS	Waste vehicles	218.14	108.88	376.88	97.28	235.00	39.00	234.71	38.97
WWA ECOWWS	Wastewater ECO	0.00		0.00		7.40	1.90	7.07	1.59
WWA PHHWWS	Wastewater PHH	9.80	0.76	11.21	0.75	5.00	1.50	4.79	1.35

Note: t/yr = metric tons per year

Table S55: Stock list

		Taipei		Vienna	
Process	Process name	Mass [t]	± Mass [t]	Mass [t]	± Mass [t]
EXA	External Anthroposphere (input related)				
EXAO	External Anthroposphere (output related				
LDF	Landfill			144,000	61,000
PHH	Private Households	27,795	11,579	131,000	35,000
DSR	Dansuie/Danube River				
TEC	Transport / Energy / Communication Infrastructure	32,027	8,325	103,000	11,000
UST	Underground Storage				
UHY	Urban Hydrosphere				
ECO	Urban Industry, Business, Services & Forestry /	215	27	40,000	14,000
UPV	Urban Pedosphere & Vegetation	831	145	4,000	1,000
VEH	Vehicles	12,014	5,911	24,000	2,000

Notes: t = metric tons

Figure S11: Cu flow system of Vienna on 1st level: Snapshot for the year 2008. The stocks are given in gram per capita (g/cap) and the flows in gram per capita per year (g/cap/yr). The stock values as well as the alteration of stocks are shown in the boxes; the flows are represented as Sankey arrows proportional to the flux rate.

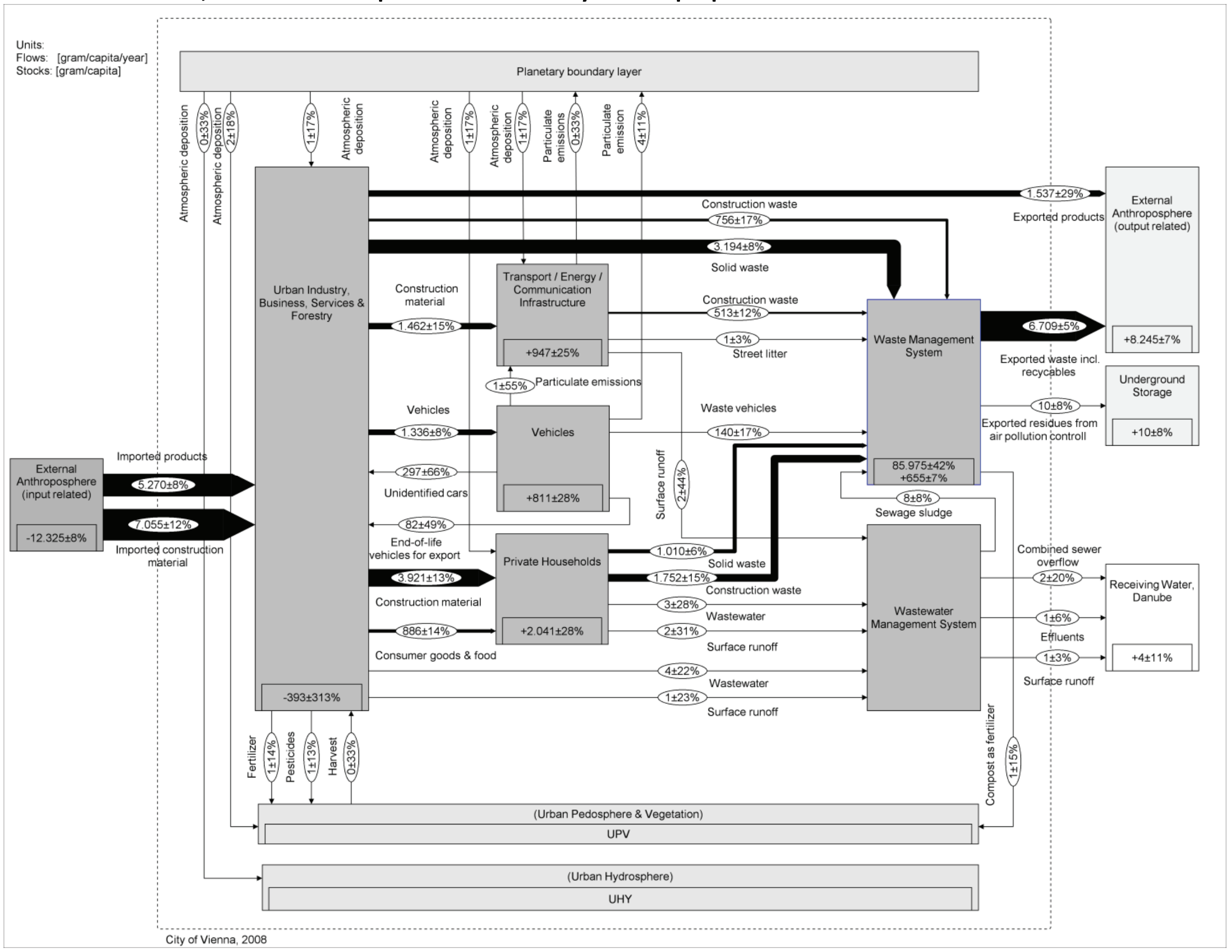

Figure S12: Cu flow system of Vienna on 2nd level: Snapshot for the year 2008. The stocks are given in gram per capita (g/cap) and the flows in gram per capita per year (g/cap/yr). The stock values as well as the alteration of stocks are shown in the boxes; the flows are represented as Sankey arrows proportional to the flux rate.

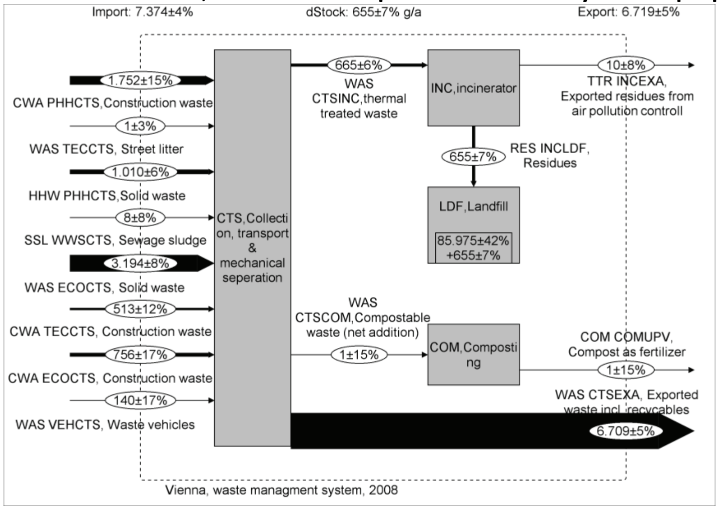

Figure S13: Cu flow system of Taipei on 1st level: Snapshot for the year 2009. The stocks are given in gram per capita (g/cap) and the flows in gram per capita per year (g/cap/yr). The stock values as well as the alteration of stocks are shown in the boxes; the flows are represented as Sankey arrows proportional to the flux rate.

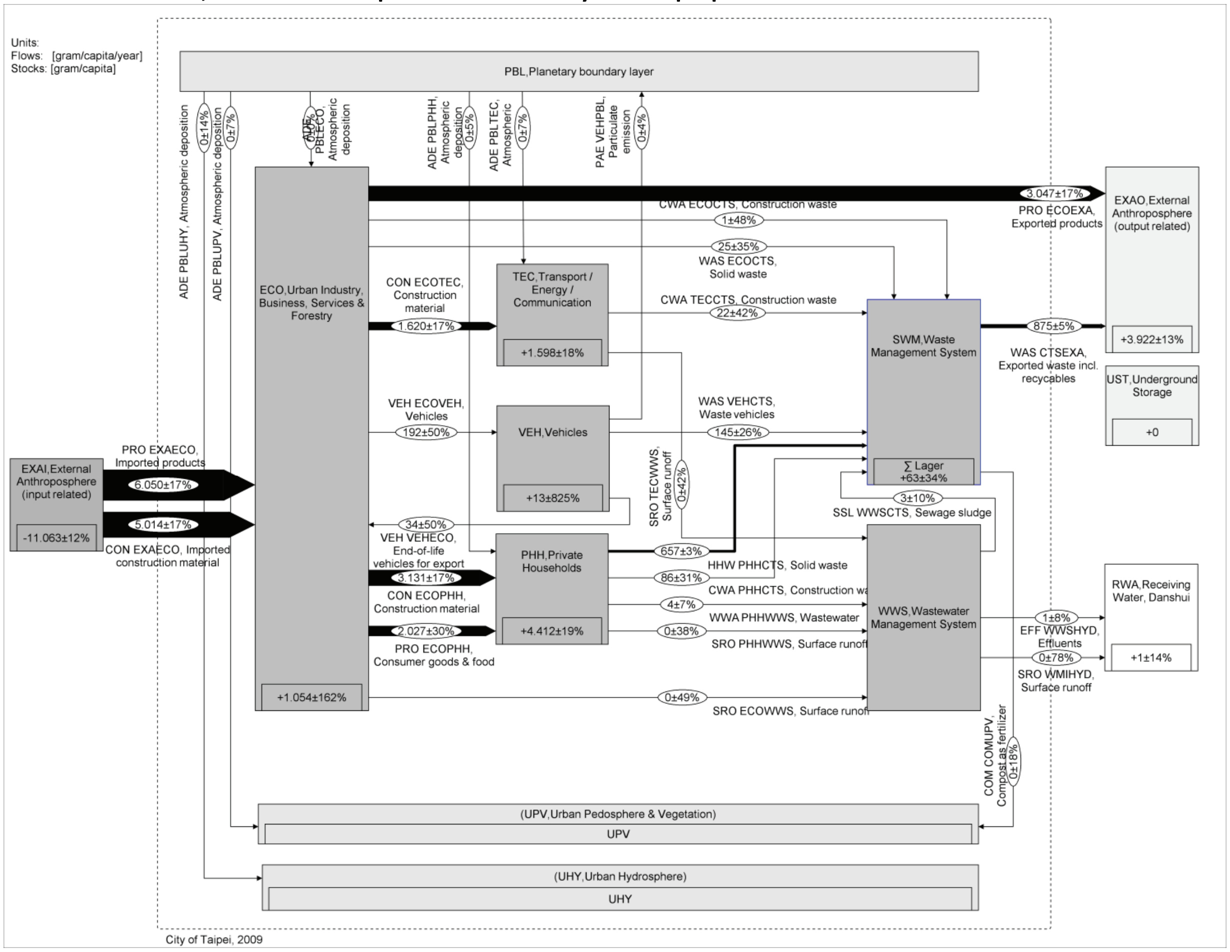

Figure S14: Cu flow system of Taipei on 2nd level: Snapshot for the year 2009. The stocks are given in gram per capita (g/cap) and the flows in gram per capita per year (g/cap/yr). The stock values as well as the alteration of stocks are shown in the boxes; the flows are represented as Sankey arrows proportional to the flux rate.

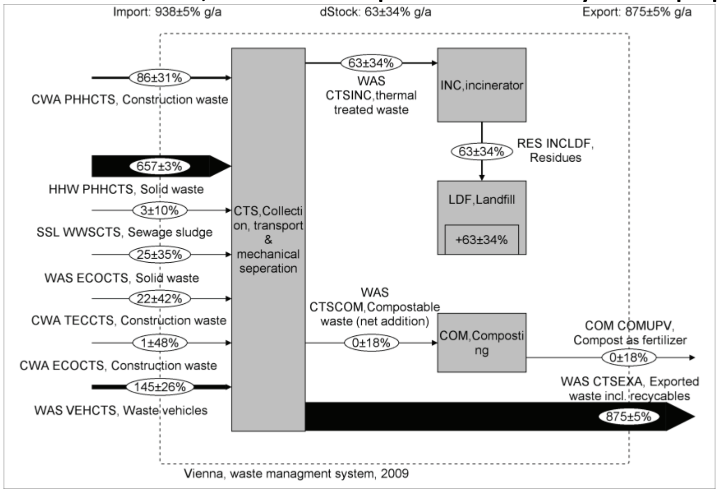

3. References

Ableidinger, M., K. Arbter, W. Hauer, S. Sciri, and U. Volk. 2007. *Wiener Abfallwirtschaftskonzept 2007: Ist-Zustand der Wiener Abfallwirtschaft*. [Viennas Waste Managment Concept 2007: Status-Quo.] Wien: Stadt Wien.

Arx, U. 2006. *Kupfer: Verbrauch, Umwelteinträge und -vorkommen*. [Copper: Consumption, environmental impacts and availability.] Bern: Bundesamt für Umwelt.

Augustyn, R., P. Riess, and R. Bachl. 2010. *Jahresbericht 2009 - Luftgütemessungen der Umweltschutzabteilung der Stadt Wien gemäß Immissionsschutzgesetz - Luft*. [Annual Report 2009 - Air Quality Measurements according to Anti-Pollution act conducted by the city of Vienna.] Wien: Magistratsabteilung 22, Stadt Wien.

Baccini, P., H. Daxbeck, E. Glenck, and G. Henseler. 1993. *Metapolis - Güterumsatz und Stoffwechselprozesse in den Privathaushalten einer Stadt*. [Metabolis - Material turnover and metabolic processes in urban private households.] Zürich: Schweizerischer Nationalfond zur Förderung der Wissenschaft.

Bergbäck, B., K. Johansson, and U. Mohlander. 2001. Urban Metal Flows – A Case Study of Stockholm. Review and Conclusions. *Water, Air, and Soil Pollution: Focus* 1(3): 3-24.

Berger, E., G. Dersch, A. Dellantonio, K. Manner, B. Möbes-Hansen, and M. Stemmer. 2011. *Kupfer als Pflanzenschutmittel - Strategie für einen nachhaltigen und umweltschoneneden Einsatz. 2. Zwischenbericht*. [Copper as pesticide - Strategies for sustainable and environmental friendly appliance.] 2. Zwischenbericht Wien: AGES Österreichische Agentur für Gesundheit und Ernährungssicherheit GmbH.

Bertram, M., T. E. Graedel, H. Rechberger, and S. Spatari. 2002. The contemporary European copper cycle: waste management subsystem. *Ecological Economics* 42(1-2): 43-57.

BMLFUW. 2011. *Bundes-Abfallwirtschaftsplan 2011*. [Federal Waste Management Plan 2011.] Wien: Bundesministerium für Land- u. Forstwirtschaft, Umwelt und Wasserwirtschaft.

Boller, M. 2002. Charakterisierung von Straßenabwasser - Emissionen und Immissionen: VSA-Fortbildungskurs 2002 - Straßenentwässerung der Zukunft.

Brunner, P. H. and D. M. Stampfli. 1993. Material Balance of a Construction Waste Sorting Plant. *Waste Management & Research* 11(1): 27-48.

Burkhardt, M., L. Rossi, N. Chevre, M. Boller, L. Steidle, J. Abrecht, F. Gächter, S. Knabl, and H. Kuppelwieser. 2005. *Gewässerschutz bei Bahnanlagen - Emittierte Stoffe im Eisenbahnbetrieb der SBB und Grundlagen zu deren Umweltverhalten*. [Water Protection along Railway Tracks - Substance Release by Railway Operation of Swiss Federal Railways (SBB) and Fundamentals of their Environmental Behaviour.] Dübendorf, Schweiz: EAWAG.

Chen, Y. G. 2008. Statistical Model of Total Metal Concentration in Bottom Ash. Master Thesis thesis, National Taiwan University, Taipei, Taiwan.

Daxbeck, H., M. Stockinger, and B. Brand. 2006. *Beitrag der Abfallwirtschaft zum Kupferhaushalt Österreich*. [Role of waste management in the Austrian copper metabolism.] Wien: Ressourcen Management Agentur.

Daxbeck, H., C. Lampert, L. S. Morf, R. Obernosterer, H. Rechberger, I. S. Reiner, and P. H. Brunner. 1996. *Der anthropogene Stoffhaushalt der Stadt Wien - N, C und Pb*. [The antropogenic metabolism of Vienna - Nitrogen, Carbon and Lead.] Wien: Technische Universität Wien.

Department of Environmental Protection of Taipei City Government. 2009. Statistics of Waste Management in Taipei.

www.dep.taipei.gov.tw/ct.asp?xItem=14974217&ctNode=39418&mp=110001. Accessed 12 December 2012.

Department of Statistics. 2007. *Estimation of Number of Vehicles-Km Statistics in Taiwan.* Taipei, Taiwan: Ministry of Transportation and Communication.

Dimitrova, D., J. Rauch, R. Gordon, and T. Graedel. 2007. *Flow and Fates of Discarded Copper in Sofia, Bulgaria, and New Haven CT, USA. Working Paper Number 12*. Yale: Yale School of Forestry & Environmental Studies.

EAK. 2009. *Tätigkeitsbericht 2008*. [Annual Report 2008.] Wien: Elektroaltgeräte Koordinierungsstelle Austria GmbH (EAK).

Environmental Protection Administration - Executive Yuan. 2010. *Yearbook of Environmental Protection Statistics, 2010*. www.epa.gov.tw/ch/DocList.aspx?unit=24&clsone=501&clstwo=178&clsthree=168&busin=4177&path=9548. Accessed 12 December 2012.

EPA. 2004. *Official Announcement of Fee Rates for Article and Container Recycling, Clearance, and Disposal (EPA Fei-Tzu No. 0930097607C)*. Taipei, Taiwan: Executive Yuan - Environmental Protection Administration (EPA).

European Copper Institute (ECI). 2011. Transport. www.eurocopper.org/copper/copper-transport.html. Accessed 7 December 2011.

Fellner, J. 2012. Personal Communication with Fellner, J., Dichte von Altablagerungen. [Waste density in old landfills.].

Fenz, R. 1999. Gewässerschutz bei Entlastungsbauwerken der Mischkanalisation. [Water protection of buildings regarding combined sewer systems.] Ph.D. thesis, Technische Universität Wien, Wien.

Figi, R., O. Nagel, M. Tuchschmid, P. Lienemann, U. Gfeller, and N. Bukowiecki. 2010. Quantitative analysis of heavy metals in automotive brake linings: A comparison between wet-chemistry based analysis and in-situ screening with a handheld X-ray fluorescence spectrometer. *Analytica Chimica Acta* 676(1-2): 46-52.

Fitzthum, R. 2009. *Wiener Landwirtschaftsbericht 2008*. [Annual agriculture report 2008.] Wien: Landwirtschaftskammer Wien.

Fricke, M. and H. U. Höhl. 2000. Schadstoffeintrag in Böden. In *Müll und Abfall*. München: Erich Schmidt Verlag.

Graedel, T. E., M. Bertram, K. Fuse, R. B. Gordon, R. Lifset, H. Rechberger, and S. Spatari. 2002. The contemporary European copper cycle: The characterization of technological copper cycles. *Ecological Economics* 42(1-2): 9-26.

Hauptverband der österreichischen Sozialversicherungsträger SV. 2011. Standardbeschäftigung in Wien nach Wirtschaftsklassen seit 2008. Wien: Magistratsabteilung 05 - Statistik.

Hausmann, M. 2005. Potential thermischer Verfahren zur stofflichen Abfallverwertung. [Potential of thermal treatment for waste treatment.] Master thesis, Institut für Wassergüte, Ressourcenmanagement und Abfallwirtschaft, Technische Universität Wien, Wien.

Holzapfel, P. and R. Riedel. 2011. Personal Communication with Holzapfel, P. and R. Riedel, Verkehrsmodell Wien. [Traffic model Vienna.], 4 December 2011.

Hoock, R. 2008. Kupfer im Automobilbau. [Copper in car industry.] *Metall* 62. Jahrgang(10): 613-617.

Huang, R. Y. 1998. *The Status Quo and Investigation Framework of Pollution and Waste Generated from Demolition*. New Taipei City: Architecture and Building Research Institute, Ministry of The Interior.

Hulskotte, J. H. J., M. Schaap, and A. Visschedijk. 2006. Brake wear from vehicles as an important source of diffuse copper pollution. *10th Int. Specialised Conference on Diffuse Pollution ans Sustainable Basin Management*, 18-22. September 2006, Instanbul. International Water Association.

I C Consultants Ltd. 2001. Pollutants in Urban Waste Water and Sewage Sludge. Luxembourg: European Communities.

ICF. 2000. *Wire in Automobiles: A one Million Tonne Market*. Newsletter Issue 32 ICF International Cablemakers Federation.

ICF. 2001. *Copper - The Strategic Material (Newsletter Issue 35)*. ICF International Cablemakers Federation.

IDC Taiwan. 2008. Sales Volume of PC in Taiwan 2008. www.idc.com.tw/about/archivenews.jsp. Accessed 11 December 2011.

Jien, S. H., C. C. Tsai, Z. Y. Hseu, and Z. S. Chen. 2011. Baseline Concentrations of Toxic Elements in Metropolitan Park Soils of Taiwan. *Terrestrial and Aquatic Environmental Toxicology* 5(1): 1-7.

Kalina, M., K. Leder, S. Kramer, and H. Puxbaum. 2000. *Nasse Deposition im Land Wien Oktober 99 - September 00*. [Wet deposition in Vienna, Oktober 99 - September 00.] Vienna: Technische Univeristät Wien. Institut für Analytische Chemie. Abteilung für Umweltanalytik.

Keoleian, G., K. Kar, M. M. Manion, and J. W. Bulkley. 1997. Industrial Ecology of the Automobile: A Life Cycle Perspective. Warrendale: Society of Automotive Engineers, Inc.

Kreiner, P. 2004. *Wiener Bodenbericht 2003: Untersuchung des Wiener Bodens auf Schwermetalle und polyaromatische Kohlenwasserstoffe.* [Viennese soil report 2003: Investigation heavy metals and hydrocarbons in Viennese soils.] Wien: Stadt Wien.

Kroiss, H., L. S. Morf, C. Lampert, and M. Zessner. 2008. *Optimiertes Stoffflussmonitoring für die Abwasserentsorgung Wiens*. [Optimized substance flow monitoring of the waste water treatment plant in Vienna.] Wien: Technische Universität Wien.

Kronberger, R. 2011. Personal Communication with Kronberger, R., MVA Inputs 2008. Wien, 21 November 2011.

Krook, J., A. Carlsson, M. Eklund, P. Frändegård, and N. Svensson. 2011. Urban mining: hibernating copper stocks in local power grids. *Journal of Cleaner Production* 19(9–10): 1052-1056.

Kubu, S. 2011. Personal Communication with Kubu, S., Roof surfaces and inclination of roofs in Vienna. 6 December 2011.

Kühnen, V. and H. E. Goldbach. 2004. *Schwermetallbilanzen verschiedener Betriebstypen: Eintragswege, Flüsse, Minderungspotential.* [Heavy metal balances of different retail formats: Inputs, pathways, reduction potential.] www.usl.uni-bonn.de/pdf/Forschungsbericht%20118.pdf. Accessed 25 November 2011. Bonn: Ministerium für umwelt und Naturschutz, Landwirtschaft und Verbrauherschutz Nordrhein-Westfalen.

Lampert, C., W. Stark, R. Kernbeis, and P. H. Brunner. 1997. *Stofffflussanalyse der Siedlungsentwässerung der beiden Regionen "Gresten" und "Grafenwörth"*. [Susbtance flow analysis of regional water discharge.] Wien: Technische Universität Wien.

Lebhart, G. 2010. *Statistisches Jahrbuch der Stadt Wien 2010*. [Statistical Yearbook of Vienna 2010.] Vienna: Magistrat der Stadt Wien, Magistratsabteilung 5 - Finanzwesen.

Lehmann, T. 2011. Personal Communication with Lehmann, T., Wastewater input into Viennas waste water system, ratio of mixed/separated sewer system in Vienna. Vienna, 20 July 2011.

Lin, J. U. 2003. A Study on the Operation Performance for the Transmission Line System of Taichung Power Supply Branch of Taiwan Power Company Master Thesis thesis, Taichung Healthcare and Management University, Taichung.

Liu, J. C. 2009. *The Project of Recycling System Review and Reducing, Recycling Investigation for wasted car (EPA-97-HA14-03-A003)*. Taipei, Taiwan: Institute of Environment and Resources.

Magistratsabteilung 05. 2011. Personal Communication with Magistratsabteilung 05, Aussenhandelsstatistik für Wien 2008. [Trade Statistic for Vienna 2008, restricted for public access.] Vienna.

Magistratsabteilung 31. 2008. *Überwachungsberichte über Abnahmebeschwerden*. [Monitoring Reports for Water Quality Assessment, restricted for public access.] Wien: Stadt Wien.

Magistratsabteilung 48. 2009. *Leistungsbericht 2009*. [Business Report 2009.] Wien: Stadt Wien.

Mitterbauer, G., S. Skutan, and H. Rechberger. 2009. *Charakterisierung der Rostasche der MVA Dürnrohr im Hinblick auf die Rückgewinnung von Metallen, Projektbericht ChaRo*. [Characterization of bottom ash in the Waste to Energy Plant Dürnrohr with a focus on metal recovery, project report ChaRo.] Wien: Technische Universität Wien.

Morf, L. S. and R. Taverna. 2006. *Monitoringkonzept zur Ermittlung von Ursachen für Veränderungen der Schwermetallgehalte im Wiener Restmüll*. [Monitoring concept to investigate the change in heavy metal concentrations in residual waste of Vienna.] Zürich: GEO Partner AG Umweltmanagement.

Morf, L. S., R. Gloor, O. Haag, M. Haupt, S. Skutan, F. D. Lorenzo, and D. Böni. 2013. Precious metals and rare earth elements in municipal solid waste – Sources and fate in a Swiss incineration plant. *Waste Management* 33(3): 634-644.

Müller, B., G. Schöller, G. Rebernig, and P. H. Brunner. 2008. *Analyse der Quellen von Kupfereinträgen in den Gleisschotter, im Auftrag der ÖBB Bau AG*. [Identification of copper sources and pathways entering ballast.] Wien: Technische Universität Wien.

ÖBB-Holding AG. 2008. *Zahlen, Daten, Fakten 2008*. [Data and Figures 2008.] Wien: ÖBB Österreichische Bundesbahn.

Odnevall Wallinder, I., B. Bahar, C. Leygraf, and J. Tidblad. 2007. Modelling and mapping of copper runoff for Europe. *Journal of Environmental Monitoring* 9: 66-73.

Odnevall Wallinder, I., B. Bahar, C. Leygraf, and J. Tidblad. 2007. Modelling and mapping of copper runoff for Europe. *Journal of Environmental Monitoring* 9(1): 66-73.

Oguchi, M., S. Murakami, H. Sakanakura, A. Kida, and T. Kameya. 2011. A preliminary categorization of end-of-life electrical and electronic equipment as secondary metal resources. *Waste Management* 31(9-10): 2150-2160.

Oonk, H., J. Hulskotte, J. Roovaart, and N. v. Duynhoven. 2005. *Emissions of brake pads, emission estimations of diffuse sources (in Dutch, version march 2005)*.

Ou, W. S., P. Y. Kuo, Y. C. Chao, and H. T. Lin. 2007. Research of Exhaust Carbon Dioxide of the Water and Electricity Pipeline in Life Cycle of Taiwan Buildings. *Chia-Nan Annual Bulletin* 33: 187-197.

Pfleiderer, S. 2011. Umweltgeochemie Stadtgebiet Wien. [Environmental geochemistry of the city of Vienna, restricted for public access.] *Seminar Ressourcenmanagement und Abfallwirtschaft*, 4 June 2011, Wien.

Purves, D. 1966. Contamination of Urban Garden Soils with Copper and Baron. *Nature* 210(5040): 1077-1077.

Rebernig, G. 2007. Methode zur Analyse und Bewertung der Stoffflüsse von Oberflächen einer Stadt. [A method to analyse and evaluate substance flows resulting from urban surfaces.]

Ph.D. thesis, Institut for Water Quality, Resource and Waste Management, Vienna University of Technology, Vienna.

Recycling Fund Management Board Taiwan. 2010a. Statistics of Certified Resource Recycling Volume. http://recycle.epa.gov.tw/Recycle/index2.aspx. Accessed 25 November 2011.

Recycling Fund Management Board Taiwan. 2010b. R-Paper in July 2010. http://recycle.epa.gov.tw/epa/rpaper/9907/1_project_01.html. Accessed.

Reimann, C., T. E. Finne, O. Nordulen, A. Arnuldussen, and P. Englmaier. 2011. The Scale of an Urban Contamination Footprint: Resuls from a Transect through Oslo, Norway. *Mapping the Chmical Environment of Urban Areas*, edited by C. Johnson, et al. Chichester, United Kingdom: John Wiley & Sons, Ltd.

Republik Österreich. 2001a. Verordnung über Qualitätsanforderungen an Komposte aus Abfällen (Kompostverordnung, BGBL. II Nr. 292/2001). Wien.

Republik Österreich. 2001b. Kompostverordnung (BGBl. II Nr. 292/2001), edited by Bundesministerium für Land- und Forstwirtschaft Umwelt und Wasserwirtschaft.

Ruhrberg, M. 2006. Assessing the recycling efficiency of copper from end-of-life products in Western Europe. *Resources, Conservation and Recycling* 48(2): 141-165.

Rule, K. L., S. D. W. Comber, D. Ross, A. Thornton, C. K. Makropoulos, and R. Rautiu. 2006. Diffuse sources of heavy metals entering an urban wastewater catchment. *Chemosphere* 63(1): 64-72.

Schnöller, J., K. Hammer, D. Clement, and P. H. Brunner. 2010. Konzept zur nachhaltigen Nutzung von Baurestmassen basierend auf der thematischen Strategie für Abfallvermeidung und Abfallrecycling der EU. Wien: Technische Universität Wien, Ressourcen Management Agentur (RMA).

Shih, F. S. 2007. Evaluation of method for dewatered sewage for waste water treatment plant – An example of Taipei city. Master Thesis thesis, National Taipei University of Technology, Taipei.

Skutan, S. 2008. Personal Communication with Skutan, S., Copper content of cables from demolished buildings. Vienna, 6 May 2011.

Skutan, S. and P. H. Brunner. 2006. *Stoffbilanzen mechanisch-biologischer Anlagen zur Behandlung von Restmüll*. [Substance balances of mechanical-biological treatment plants for residuel waste.] Wien: Technische Universität Wien.

Skutan, S. and H. Rechberger. 2007. *Bestimmung von Stoffbilanzen und Transferkoeffizienten für die Linie II der MVA Wels*. [Determination of substance balances and transfer coefficents of the incinerator in the city of Wels.] Wien: Technische Universität Wien.

Sörme, L. and R. Lagerkvist. 2002. Sources of heavy metals in urban wastewater in Stockholm. *The Science of The Total Environment* 298(1-3): 131-145.

Sörme, L., B. Bergbäck, and U. Lohm. 2001a. Century Perspective of Heavy Metal Use in Urban Areas. A Case Study in Stockholm. *Water, Air and Soil Pollution: Focus* 1(3-4): 197-211.

Sörme, L., B. Bergbäck, and U. Lohm. 2001b. Goods in the Anthroposphere as a Metal Emission Source A Case Study of Stockholm, Sweden. *Water, Air, and Soil Pollution: Focus* 1(3): 213-227.

Spatari, S., M. Bertram, K. Fuse, T. E. Graedel, and H. Rechberger. 2002. The contemporary European copper cycle: 1 year stocks and flows. *Ecological Economics* 42(1-2): 27-42.

Spiegel, H. 2003. Atmospheric deposition of heavy metals onto arable land in Austria. *Concerted Action AROMIS*, Banz - Germany. Austrian Agency for Health and Food Safety.

Spitzbart, M. 2012. Personal Communication with Spitzbart, M., WEEE Recycling in Vienna. Vienna.

Statistik Austria. 2008. Kraftfahrzeuge - Bestand. [Number of registered vehicles.] www.statistik.at/web_de/statistiken/verkehr/strasse/kraftfahrzeuge_-_bestand/index.html. Accessed 3 August 2011.

Statistik Austria. 2009a. Kfz-Neuzulassungen Jänner bis Dezember 2008 [Number of new registered vehicles.] www.statistik.at. Accessed 8 February 2012.

Statistik Austria. 2009b. Kfz-Gebrauchtzulassungen 2008. [Number of re-registred cars 2008.] www.statistik.at. Accessed 1 March 2012.

Statistik Austria. 2009c. Hauptergebnisse der Leistungs- und Strukturstatistik 2008 im Produzierenden Bereich nach Abteilungen. Wien.

Statistik Austria. 2012. Bevölkerung Österreichs 2001-2050 nach Haushaltsgröße. [Austrian Inhabitants 2001-2050.] www.statistik.at/web_de/statistiken/bevoelkerung/demographische_prognosen/haushalts_und_familienprognosen/023535.html. Accessed 1 May 2012.

Struckl, W. M. 2007. Green Line - Umweltgerechte Produktentwicklungsstrategien für Schienenfahrzeuge auf Basis der Lebenszyklusanalyse des Metrofahrzeuges Oslo. [Green Line - Sustainbale production strategies for rolling stock based on life cycle assessement of subways in Oslo.] PhD thesis, Institut für Konstruktionswissenschaften und Technische Logistik (E 307), Technischen Universität Wien, Wien.

Taverna, R., C. Rolland, C. Böker, and A. Kirchner. 2011. *Routinemäßiges Stoffflussmonitoring auf der MVA Spittelau - Messperiode 1.5.09-30.4.10*. [Routine monitoring measaures of the incinerator Spittelau in Vienna - monitoring period: 1.5.09-30.4.10.] Zürich: GEO Partner AG, Magistratsabteilungen 22 und 48 der Stadt Wien, Fernwärme Wien GmbH.

Tomenendal, A. 2011. Personal Communication with Tomenendal, A., Freshwater Supply Vienna. 19 July 2011.

Townsend, T., T. Tolaymat, K. Leo, and J. Jambeck. 2004. Heavy metals in recovered fines from construction and demolition debris recycling facilities in Florida. *Science of The Total Environment* 332(1-3): 1-11.

Truttmann, N., O. Cencic, J. Fellner, and H. Rechberger. 2005. *Technisch-naturwissenschatliche Grundlagen zur Auswahl von Bewirtschafuntsszenarien für Elektroaltgeräte (TABEA)*. [Fundamentals for operation-scenarios of WEEE.] Wien: Technische Universität Wien.

Umweltbundesamt. 2000. *Qualität von Komposten aus der getrennten Sammlung – Ergebnisse der Kompostanalysen*. [Qualtity of compost from seperated collection.] Umweltbundesamt Österreich.

Umweltbundesamt. 2001. *Begrenzung von Schadstoffeinträgen in landbaulich genutzten Böden*. [Limitation of substance inputs in agricultural soils.] UBA-Texte 59/01 Berlin: Umweltbundesamt.

Umweltbundesamt Österreich, A. A. 2012. Altablagerungen >25.000 m³ in Wien (Dataset), edited by S. Granzin. Wien.

USDA. 2011. National Nutrient Database. http://www.nal.usda.gov/fnic/foodcomp/search/. Accessed

van Beers, D. and T. Graedel. 2003. The magnitude and spatial distribution of in-use copper stocks in Cape Town, South Africa. *South African Journal of Science* Volume 99(Issue 1 & 2): 61 - 69

van Beers, D. and T. E. Graedel. 2007. Spatial characterisation of multi-level in-use copper and zinc stocks in Australia. *Journal of Cleaner Production* 15(8-9): 849-861.

Weinmar, K. 2011. Personal Communication with Weinmar, K., Kompostproduktion und -verbrauch in Wien. [Compost production and consumption in Vienna.], August 2010.

Wien Energie. 2010. *Geschäftsbericht 2009/10*. [Business Report 2009/10.] Wien: Wiener Stadtwerke.

Wiener Linien. 2008. *Gleistabelle*. [Network lenghts - Tram.] Wien: Wiener Linien.

Wiener Linien. 2009. *Betriebsangaben 2009*. [Operational data 2009.] www.wienerlinien.at/media/files/2011/wl_betriebsangaben_2009_19482.pdf. Accessed Wien:

Wiener Umweltschutzabteilung MA22. 2011. Personal Communication with Wiener Umweltschutzabteilung MA22, Abfallstatistik Wien 2008 (Waste statistic for Vienna 2008). Wien, 30 June 2011.

Winther, M. and E. Slento. 2010. *Heavy metal emissions for danish road transport (NERI Technical Report no. 780)*. Aarhus, Denmark: Aarhus University.

Wirtschaftskammer Österreich. 2012. Rahmendaten - Importe und Exporte gebrauchter Fahrzeuge. [Data about imports and exports of second life cars.] http://wko.at/fahrzeuge/main_frame/statistik/JB/Seite5.11%202012.pdf. Accessed 9 August 2012.

Wittmer, D. M. A. G. 2006. Kupfer im regionalen Ressourcenhaushalt - Ein methodischer Beitrag zur Exploration urbaner Lagerstätten. [Copper in the regional metabolism - a methodological contribution for explorating urban resource stocks.] Ph.D. thesis, ETH Zürich, Zürich.

Wocilka, H. and J. Höfner. 2011. Personal Communication with Wocilka, H. and J. Höfner, Kupferdächer in Wien. [Copper roofs in Vienna.], 30 September 2011.

Woisetschlaeger, G., S. Musser, A. Lindlbauer, and W. Wruss. 2000. *Bestandsaufnahme der Bodenbelastung in Wien PAK, KW, Schwermetalle (Gesamtgehalte)*. [Assessement of soil contamination in Vienna.] Wien: Magistratsabeilung 22.

Zethner, G., R. Sattelberger, and A. Hanus-Illnar. 2007. *Kupfer und Zink im Wirtschaftsdünger von Schweine und Geflügelmastbetrieben*. [Copper and Zinc in manure from pigs and poultries.] Wien: Umweltbundesamt Österreich.

Zhang, L., Z. Yuan, and J. Bi. 2012. Estimation of Copper In-use Stocks in Nanjing, China. *Journal of Industrial Ecology* 16(2): 191-202.

Zuo, X., D. Fu, and H. Li. 2012. Speciation distribution and mass balance of copper and zinc in urban rain, sediments, and road runoff. *Environ Sci Pollut Res Int* 19: 4042-4048.
